# Design of a minimal, allosteric, and ATPase-like machine using mechanical linkages

**Published:** 2025-12-19

**Authors:** Tosan Omabegho

## Abstract

ATPases cyclically convert chemical energy in the form of ATP gradients into directed motion inside cells. To function, ATPases rely on allosteric communication between at least two binding sites—an internal signaling mechanism that is not well understood. Here, we model an ATPase-like machine by using a system of mechanical linkages to recreate negative allosteric coupling between two binding sites and generate cycles in which the sites alternate occupancy. The ATPase analog has two mechanical degrees of freedom and two discretized binding sites: one for the ATP, Pi and ADP analogs, and one for an allosteric effector analog. The geometry of the ATPase analog allows stepwise binding reactions at each site to capture the two degrees of freedom in a mutually exclusive way. Consequently, the enzyme interconverts between multiple rigid and partially rigid forms, such that neither site can be fully bound when both sites are occupied. Two mechanisms work together to generate an enzymatic cycle: one, in which the tighter-binding ATP analog can bind and displace the effector from the enzyme; and a second, in which flexibility introduced by splitting the ATP analog into two pieces (catalysis) allows the effector to rebind and displace the products (ADP analog). We show that cleavage (forward catalysis) and ligation (reverse catalysis) alter the rigidity of the enzyme complex equivalently to binding and dissociation, respectively, but must do so more slowly for effective cycling to take place. Simple designs for synthetic systems that mimic ATPase monomers can be derived from this work.

## Introduction

ATPases are enzymes that split ATP into Pi and ADP in order to carry out various transport functions. ATPases include cytoskeletal motors [1], most membrane pumps [2], translocases and packaging motors [3] and other families of motors. A long-term goal is to create synthetic molecular machines that mimic the abilities of ATPases. However, ATPases, like many other proteins, rely on allosteric communication to operate [4, 5], and developing mechanisms and concepts to mimic allostery is a difficult problem [6].

Allostery is intramolecular communication between at least two non-overlapping reactive sites such that binding or catalysis of a ligand at one site affects ligand affinity at the other site [7, 8]. The sites and the ligands that bind to these sites are said to be allosterically “coupled”. ATPases mediate coupling between ATP (and ADP and Pi) and a chemically different ligand, or allosteric effector [4, 5], which is not consumed in the enzymatic process, as is ATP. For example, for myosin, the nonconsumed, coupled ligand is actin; for kinesin and dynein, it is microtubule; and for helicases, it is DNA. The variety of ATP and ligand pairings that exist for ATPases and the variety of structures that have evolved to mediate allostery between them suggest a common language of allosteric communication is at work, a language that can possibly be mimicked if not yet fully understood.

Recently, a number of works have used a mechanical linkage (framework) abstraction to design synthetic allosteric structures [9–19]. In the framework abstraction, molecules are viewed as networks of rigid bars or modules connected by flexible hinges, which change shape in response to a signal [20, 21]. These works combine the abstraction with different materials, making use of elastic networks [12–14], synthetic DNA [9–11, 16, 18], or synthetic proteins [17, 19]. Most of the experimental works demonstrate allosteric structures that can convert a binding reaction at one site into a chemical signal at a non-overlapping site. For example, in several works they demonstrate how ligand binding can trigger dissociation of an internal bond or of another ligand [9–11, 16, 17, 19]. And in one of these works [17], they show how ligand binding in one subunit can increase ligand affinity in repeated subunits. By contrast, the elastic network studies focus on computationally evolving networks to transmit conformational change between non-overlapping sites. In place of reactions with abstracted ligands, strain is used to represent chemical reactivity.

The behavior in all these works can be characterized as switchlike, where in response to some external stimuli, the frameworks change from one conformational state to another to reach some new equilibrium or energy minimum. The initial concept and models of allostery were developed to explain switchlike behavior in signaling proteins [22, 23], most famously hemoglobin [7, 24]. The linkage works explicitly or implicitly take inspiration from these early models, in particular, from the idea that two states, at a minimum [23], can describe allosteric switching in a protein subunit.

However, to reproduce the autonomous and cyclic activity that characterizes ATPases, one-way allosteric switching is not sufficient. To operate cyclically, at a fixed thermodynamic potential of ATP, ATPases go through two phases of allosteric switching in which intermediate states, most importantly the product-bound state, play a critical role. The phases result in the exchange of both nucleotide and effector, where each binds and releases over the course of the cycle, and each acts as an exchange factor for the other in a mutually dependent (reciprocal) manner [25–27]. Synthetic systems that emulate ATPases and their ability to autonomously cycle by switching allosterically and reciprocally have not been described.

**Fig. 1.**
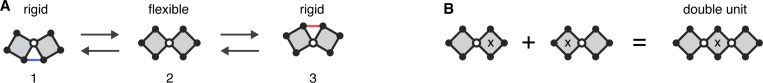
Three-state switch. **A,** A single unit. The single unit consists of two rigid squares connected at a flexible node (white). Three conformations are possible: a flexible one (2), in which no additional bars are connected; a rigid one (1), in which a bar (blue) connects the two bottom nodes to form a rigid triangle; and another rigid one (3), in which a bar (red) connects the two top nodes to form a rigid triangle. **B,** A double unit. A double unit is formed by merging one square (marked with ‘x’) of two single units. The double unit has four completely rigid conformational states, and four partially rigid conformational states, as described later in more detail.

Here, we use linkages to model an ATPase-like machine. We explore a number of additional concepts previously explored by others: discretized binding pockets [28–30]; enzyme flexibility in relation to allostery [30] and hydrolysis [31–34]; timescale separation [35–37]; spontaneous dissociation from weak bonds [32, 36–39]; facilitated dissociation [36, 37, 39]; and floppy mechanical modes [40, 41]. The building block of the model is an allosteric switch first described by Koshland to model competitive inhibition [30], which we convert into a linkage (Fig. 1). The switch has a single mechanical degree of freedom and three conformational states: two rigid states and one flexible intermediate. Nodes on the top and bottom form two discretized binding sites. The ATPase analog is two connected switch units (Fig. 1B). The geometry gives it two mechanical degrees of freedom. Four additional linkage reactants, which are simple chains of bars, complete the ATPase system. These linkages are analogs for ATP, Pi, ADP, and an allosteric effector. The analogs for ATP, Pi, and ADP bind to one site on the ATPase analog, and the effector binds to the other.

We make two basic assumptions that govern how the linkages interact with one another in the model. First, we assume that the ATPase analog (enzyme) and the complexes it forms are mechanically equilibrated and free of elastic bias. This assumption has been made in some models of biomolecular machines [42, 43], and in the design of mechanical networks with floppy modes [40, 41]. Consequently, a conformational change in the enzyme linkage—to a more flexible or a more rigid state—results only from a change in the connectivity of the network, meaning the removal or addition of bars, rather than from an application of strain. Second, we use the stepwise addition and removal of bars [44–46], which, respectively, create rigidity and flexibility, as analogs for thermally driven bond making and bond breaking. Motivated by an earlier version of this paper, reference [47] used this bonding abstraction to develop a formal model of interacting linkages. They also showed that complex behavior can be achieved in an idealized topological model, which considers solely the graph connectivity of the linkages.

To mimic negative allosteric coupling, the four reactants are designed to have bar lengths that prevent both sites on the enzyme from being fully bound at the same time. The arrangement creates a dynamic situation in which the enzyme interconverts between multiple rigid and partially rigid states when both sites are occupied. By tuning the off rates at each node, the arrangement allows one reactant to bind and displace another from the enzyme in a stepwise and stochastic manner. We refer to the mechanism as an *allosteric displacement*.

To mimic hydrolysis, the ATP analog is allowed to be cleaved in two at a non-binding moiety (node) after it binds. The design is inspired by the use of DNA enzymes in synthetic systems [32]. Cleaving introduces flexibility and generates the Pi and ADP analogs. Importantly, cleaving does not affect binding nodes, and thus the Pi and ADP analogs bind to the same nodes as does the ATP analog. Using stochastic simulations, we show how the hydrolysis mimic enables continuous cycles of allosteric displacement to take place between the system of five linkages. During each cycle, the ATP analog displaces the effector, the ATP analog is cleaved, and the effector displaces the ADP analog to reset the cycle. We also characterize the multiple ways in which the system can go through futile cycles.

The main result is showing how an autonomous and allosterically operating enzymatic cycle can be constructed with a consistent set of reaction rates for catalysis and for binding to and dissociating from a set of weak bonds. These rates remain the same over the course of the cycle—they are not altered depending on the stage of the cycle the enzyme is in or the identity of the reactant active at that stage. Additionally, we give a simple interpretation for what reversible catalysis accomplishes. Forward catalysis frees a mechanical degree of freedom but must do so slowly enough for the bound effector to be displaced first and not compete for the degree of freedom. Conversely, reverse catalysis traps the degree of freedom but must do so slowly enough for a new effector to bind and capture the degree of freedom.

## Results

### Basic description of the system

This section describes, in the following order, the chemical cycle of the system, the linkage reactants, the rules that govern complex formation, and the construction of the reaction network.

The chemical cycle of the system is derived from the cycle of a myosin or dynein monomer (see SI 1.1). There are five reactants: the ATPase analog (the enzyme); the ATP analog (S, for ‘substrate’); the Pi analog (P1, for ‘product 1’); the ADP analog (P2, for ‘product 2’); and the effector analog (L, for ‘ligand’). With these five reactants, the cycle can be stated this way (note that because every state is a bound state of the enzyme, the enzyme is not listed):

(1) $$\overset{\text{S displaces L}}{\overset{︷}{\left\{\text{L}\}\overset{1}{\to }\{\text{S}/\text{L}\}\overset{2}{\to }\{\text{S}\right\}}}\overset{✂,3}{\to }\{\text{P}1,\text{P}2\}\overset{4}{\to }\overset{\text{L displaces P2}}{\overset{︷}{\left\{\text{P}2\}\overset{5}{\to }\{\text{P}2/\text{L}\}\overset{6}{\to }\{\text{L}\right\}}}--\to$$

There are six reactions: 1, S binds; 2, L dissociates; 3, S is cleaved; 4, P1 dissociates; 5, L binds, and 6, P2 dissociates. Each reaction connects two states, with six states in total (in curly braces). The backslash (/) in each state separates the reactants that bind at the two binding sites ({substrate site/ligand site}). No backslash indicates that only one site is occupied. Thus, in the first state, {L}, the ligand site is occupied by a single molecule of ligand, denoted by L, and the substrate site is empty. Each displacement sequence (S displaces L, and L displaces P2) includes a binding reaction, followed by a dissociation. The reaction sequence thus says, substrate displaces ligand (1 & 2), substrate is cleaved (3), P1 spontaneously dissociates (4), and finally ligand displaces P2 (5 & 6).

**Fig. 2.**
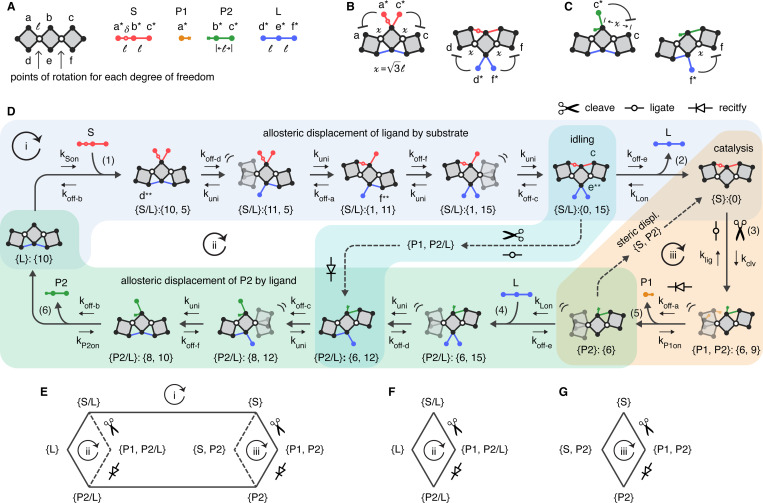
The linkage system. **A,** Five molecules of the linkage system. The enzyme consists of three rigid squares of edge length ℓ, connected at two points of rotation. Nodes a, b and c form the binding site for the substrate, P1 and P2. Nodes d, e and f from the binding site for the ligand. Complimentary nodes on S, P1, P2 and L, are denoted by the same letter with the superscipt “*”. S and L consist of three nodes connected by two bars of length ℓ, where the center nodes on each are points of flexibility. S is split into P1 and P2 at the special node δ. **B,** Left, L blocks S. When L is bound at all three nodes, it bends the enzyme down, placing the two outer nodes out of reach for S. The increased distance between S’s nodes is $x=\sqrt{3}\ell$. Right, symmetric blocking of L by S. **C,** Left, L blocks P2. Right, symmetric blocking of L by P2. **D,** Target cycle and two futile pathways. The target cycle (cycle i; outside black path going clockwise) takes place in thirteen reversible reactions. Each state name (e.g. {S/L}:{10,5}) indicates its composition ({S/L}) and unique linkage state ({10,5})(see SI 1.2). Forward and reverse rates governing each reaction are labeled on each edge (e.g. between {S/L}:{10,5} and {S/L}:{11,5}, the forward rate is ${\text{k}}_{\text{off-d}}$, and the reverse rate is ${\text{k}}_{\text{uni}}$). Reactions numbered 1–6 mark the same changes in composition that take place Eq. 1. Starting at {L}, the allosteric displacement of L by S (light blue bubble) takes place in four intramolecular steps between 1 and 2, and completes with the enzyme bound to only S ({S}). Subsequently (orange bubble), S is cleaved into P1 and P2 (3, ✂). Directly following cleavage, or rounds of ligation (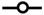) and cleavage, P1 spontaneously dissociates (4), which rectifies catalysis and leaves the enzyme bound to only P2 ({P2}). After L binds (5), the allosteric displacement of P2 by L (green bubble) takes place in three intramolecular steps between 5 and 6, and returns the enzyme to the start of the cycle and bound to only L {L}. The two futile pathways are *idling* (blue bubble and dashed line) and *steric displacement* (orange bubble and dashed line). Both pathways are abbreviated, and shown without figures. The change in the bound state along each path is stated: {P1, P2/L} in idling; and {S, P2} in steric displacement. Combined with states along the target path, idling is cycle ii, and steric displacement is cycle iii. **E,** Simplified network version of the system. Each node here represents a set of states with different intramolecular conformations. **F,** Idling cycle. **G,** Steric displacement cycle.

The linkage system generates the hidden microstates and reactions required to explain the displacement reactions and explain how the displacements are linked together by catalysis (Fig. 2). These microstates and reactions lie within the {S/L} and {P2/L} states in Eq. 1. They reflect *intra*molecular changes that take place when both multivalent binding sites are occupied. In the complete cycle, {S/L} and {P2/L} are both expanded into a series of microstates. Each microstate in each series has the same reactants but a different multivalent configuration. Microstates are constructed from a set of basis states, where each basis state reflects a unique connection between reactant and enzyme (see SI 1.2.1).

As described in the introduction, the enzyme consists of two connected switch units. The arrangement creates a chain of three equivalent rigid squares of edge length ℓ that are flexibly connected at two hinge nodes (Fig. 2A). The resulting linkage has two mechanical degrees of freedom. Chemical specificity is added by defining six nodes on the enzyme to be unique monovalent binding sites for six complementary nodes on the four reactants. The three nodes on top (a, b, and c; red) form the multivalent binding site for substrate, P1, and P2, and the three nodes on the bottom (d, e, and f; blue) form the binding site for the ligand. Chemical specificity means that node binding is specific. Consequently, substrate, P1, and P2 cannot bind to the ligand site, and ligand cannot bind to the substrate site. In addition to its three binding nodes, the substrate contains a catalytic moiety, named δ, where it is split into P1 and P2 when bound to the enzyme. The split takes place on the left side of the enzyme, between the gray-colored squares.

Any single node interaction between the enzyme and a reactant forms a flexible connection. However, two adjacent node interactions create rigidity, as each divalent connection forms a triangle when bracing a hinge (Figs. 2B & 2C). Three adjacent node interactions brace both hinges and thus make the whole enzyme rigid. The different rigid complexes bend one or both sides of the enzyme linkage up, down, or up and down, relative to the center square. These opposing bends create the basis for negative allosteric coupling in the system. When the ligand is fully bound to all three nodes and bends the enzyme down, the nodes of the substrate’s binding site are spread out, allowing the substrate to bind only a single node at a time (Fig. 2B, left). Likewise, trivalent substrate binding bends the enzyme upwards and geometrically inhibits ligand binding (Fig. 2B, right). The same negative relationship holds for ligand and P2 binding, but only on the right side, where P2 can bind two nodes (Fig. 2C). The full set of restrictions is described in SI 1.2.2.

The geometric restrictions were used to enumerate all states in the system and connect them together to form a reaction network. Any two states connected in the network differ only by one node connection and thus can interconvert to one another (see SI 1.2). There are six possible interconversions, or linkage reactions (named before arrow), each of which maps to a chemical reaction (named after arrow):

- connect linkages → intermolecular binding
- increase the connection → intramolecular binding
- decrease the connection → intramolecular dissociation
- disconnect linkages → intermolecular dissociation
- split substrate → cleavage
- make substrate → ligation

We assigned reaction rates (Table 1) to the six reaction types in the following way. Intramolecular binding (II: k_uni_) was assigned to be the fastest rate by several orders of magnitude and serves as the speed limit for all other parameters. This assignment reflects the idea that once a reactant makes an initial connection to an enzyme, additional interactions should take place on a relatively rapid timescale because of the small distances involved, which in turn favors multivalent binding (ref!). Intermolecular binding $\left(\text{I}:{\text{k}}_{\text{Son}},{\text{k}}_{\text{Lon}},{\text{k}}_{\text{P}1\text{on}},{\text{k}}_{\text{P}2\text{on}}\right)$ ranges from fast to slow, depending on the “concentration” of the reactant binding (denoted by the subscript), where molar concentrations were converted to numbers for the stochastic simulations (see SI 1.3). Intramolecular dissociation (III) and intermolecular dissociation (IV) are both defined as “off” rates for the nodes $\left({\text{k}}_{\text{off-a}},{\text{k}}_{\text{off-b}},{\text{k}}_{\text{off-c}},{\text{k}}_{\text{off-d}},{\text{k}}_{\text{off-e}},{\text{k}}_{\text{off-f}}\right)$. These rates represent intramolecular dissociation if the reactant is bound by more than one node and represent intermolecular dissociation if it is bound by a single node. The off rates were assigned to be much slower than ${\text{k}}_{\text{uni}}$, and all except ${\text{k}}_{\text{off-b}}$ are faster than cleavage and ligation. Finally, cleavage (V: ${\text{k}}_{\text{clv}}$) and ligation (VI: ${\text{k}}_{\text{lig}}$) were assigned to be slow, which helps the system oscillate between substrate and ligand binding and is discussed in detail later.

To control which molecule wins in each displacement competition, the off rates were assigned to establish a hierarchy in which substrate binds the tightest, followed by ligand, P2, and finally P1. Because the log of each off rate corresponds to a binding energy, ε, for each node, where $\varepsilon_{\text{node}}\sim \ln\left({\text{k}}_{\text{off-node}}\right)$ (see SI 1.4), the hierarchy constrains the sums of the binding energies for each binding site or portion of the site, such that they obey the following inequality:

(2) $$\overset{\varepsilon_{\text{S}}}{\overset{︷}{\varepsilon_{\text{a}}+\varepsilon_{\text{b}}+\varepsilon_{\text{c}}}}<\overset{\varepsilon_{\text{L}}}{\overset{︷}{\varepsilon_{\text{d}}+\varepsilon_{\text{e}}+\varepsilon_{\text{f}}}}<\overset{\varepsilon_{\text{P2}}}{\overset{︷}{\varepsilon_{\text{b}}+\varepsilon_{\text{c}}}}<\overset{\varepsilon_{\text{P1}}}{\overset{︷}{\varepsilon_{\text{a}}}}$$

where the energies are negative. This hierarchy applies to situations in which two molecules are pitted against each other and compete for binding. The two competing molecules can be substrate and ligand, substrate and P2, or ligand and P2. The hierarchy means that substrate will win both competitions, and ligand will win against P2. When substrate displaces P2, we call this a *steric displacement*. Its regulation is discussed in more detail below, in the context of the cycle and Fig. 2D.

### Operation of the machine

Fig. 2D shows the stepwise operation of the machine along the target cycle (cycle i, outside) and along two futile cycles (cycles ii and iii, inside)(see SI 1.5 for a description of all possible pathways). The target cycle (Movies 1 & 2, SI 1.6) follows the same six intermolecular steps defined in Eq. 1 but includes the hidden microstates that take place between them and mechanistically explains the displacement reactions. In this completed cycle, state {S/L} is extended to five microstates and {P2/L} to four, which extends the sequence in Eq. 1 from six to thirteen states. Each microstate has a macrostate designation (e.g. {S/L}), which is the set of bound reactants, and a microstate designation (e.g. {5, 10}), which is the set of basis states from which it is composed. The rate for each forward (clockwise) and reverse (counterclockwise) reaction is given along each edge that connects the states together.

Displacement sequences can be viewed as a competition for the two hinges or the available degrees of freedom. During the displacement of ligand by substrate (Fig. 2D; light blue bubble and clockwise direction), substrate captures the two hinges from ligand. Conversely, in the reverse direction (counterclockwise), ligand captures them from substrate. In both directions of the sequence, the enzyme starts and ends in a completely rigid state. The forward direction is biased by the rate assignments, which allow substrate to hang on to each of its nodes longer than ligand and maintain possession of the hinges. Or, equivalently said, ligand releases its nodes sooner than substrate, freeing up the hinges for capture by substrate. The same competitive process allows ligand to displace P2 (green bubble), but in this case, ligand immediately takes control of the hinge on the left, which is unoccupied when ligand binds (reaction 4). Graphically, it is important to understand that along the target cycle in Fig. 2 (cycle i), the ligand that displaces P2 is a different ligand than the ligand displaced by substrate.

The cleavage reaction (Fig. 2D, orange bubble; reaction 3, ✂) lies between the two displacement reactions and connects them together. Like intramolecular dissociation from a node, cleaving also releases a hinge and creates flexibility. The flexibility it introduces allows ligand to displace P2, recapture the enzyme’s degrees of freedom, and reset the cycle. After cleavage takes place, forward progress is biased by assigning that ligation take place more slowly than P1 dissociation $\left({\text{k}}_{\text{lig}}<{\text{k}}_{\text{off}-\text{a}}\right)$. Whereas ligation reintroduces rigidity in the reverse direction, P1 dissociation rectifies the flexibility created by cleavage in the forward direction (reaction 5, 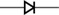). With flexibility rectified on the left side of the enzyme, ligand can easily bind and displace P2, resetting the cycle.

The two futile pathways depicted—*idling* and *steric displacement*—diverge from the target path just before catalysis begins and right after catalysis ends, respectively. Idling (Fig. 2D, blue bubble) occurs when ligand stays bound through catalysis, instead of dissociating, and is a natural consequence of the design. Idling forms a cycle (cycle ii) that bridges the very end of the ligand displacement sequence ({S/L}:{0,15}) to the middle of the P2 displacement sequence ({P2/L}:{6,12}). Graphically, it is important to understand that the same ligand remains bound along the idling cycle. It does not change as it does along the target cycle. Idling is defined as futile because no exchange of ligand takes place, even though one substrate is consumed in the cycle. Evidence suggests idling takes place in myosin [48, 49]. To inhibit idling, the cleavage rate was assigned to be slower than the slowest rate at which ligand dissociates from its nodes $\left({\text{k}}_{\text{clv}}<{\text{k}}_{\text{off}-\text{e}}\right)$. Ligand is depicted dissociating from node e in Fig. 2D, but any of the three is possible. While the ligand meant to be displaced by substrate cannot idle, the second ligand that binds, which displaces P2, can idle if it binds before cleavage. If this second ligand idles, we define this as a special case of a productive cycle (see SI 1.5, pci). It is productive because ligand exchange still takes place.

Steric displacement (Fig. 2D, dashed orange path in orange bubble) is a more extreme case of futile behavior in which turnover occurs without any involvement of ligand. Steric displacement occurs if, after P1 dissociates, substrate binds and displaces P2 before ligand displaces P2. Steric displacement forms a futile cycle (cycle iii) with catalysis and rectification. Steric displacement is mechanistically related to facilitated dissociation [36, 37] and DNA strand displacement [50, 51]. Its definition as futile follows from how ATPases operate. In many ATPases, ATP turnover (ATP binding and Pi and ADP exhaust) drives another binding process by also depending upon that process, often to perform nucleotide exchange (product exhaust) [25–27, 52, 53]. Along the steric displacement cycle, substrate rather than ligand accomplishes product exhaust, so substrate turnover no longer depends on ligand binding.

To inhibit steric displacement, we assigned node b—the central node at the substrate binding site and one of the two nodes to which P2 binds—to have the slowest off rate $\left({\text{k}}_{\text{off-b}}\right)$. This assignment allows P2 to inhibit invading substrate from binding two adjacent nodes on the enzyme. After P1 dissociates, substrate can begin a steric displacement of P2 by binding at node a. To make a second node connection, substrate must wait for P2 to dissociate from b. However, because substrate’s association with node a is weaker than P2’s with b, it is more probable for any single substrate that binds to a to spontaneously dissociate before P2 dissociates from node b. This arrangement favors the allosteric displacement of P2 by ligand over steric displacement by substrate. In contrast to substrate, ligand can immediately make two node associations with the enzyme (at d and e) after the dissociation of P1 rectifies catalysis. And once bound, ligand can complete the displacement of P2 either by P2 dissociating from node b or node c.

The two different ways in which the futile cycles are futile—idling, which makes substrate turnover dependent upon a single ligand, and steric displacement, which makes substrate turnover completely independent of ligand—highlight how productive cycling is designed to be a controlled oscillation between ligand turnover and substrate turnover. The futile cycles destroy the oscillation.

Finally, Figs. 2E, 2F, and 2G are graph representations of the target and futile cycles. They chart only intermolecular changes and catalysis and are used in place of the full depiction in the remaining figures of the main text. The target cycle (cycle i), as defined in Eq. 1, can be followed clockwise around the outside path of Fig. 2E (blue path).

### Basic simulations

Stochastic simulations were done on the system’s reaction network, which includes all possible states the enzyme can make with its four reactants. Simulations were performed using the next reaction method [54] in StochPy [55]. The simulations represent the behavior of a single molecule of enzyme in solution with multiple copies of the four reactants, where the copy numbers of the reactants are interpreted as concentrations (SI 1.3). In the current analysis, we focused on characterizing and counting occurrences of the different turnover pathways (target and futile), to confirm particular sequences of operation. We did not analyze dwell times (time spent bound to the enzyme by each reactant) or cycle times.

**Fig. 3.**
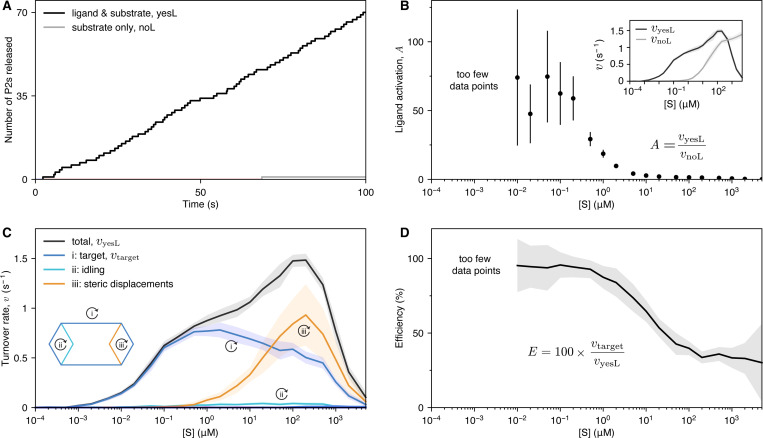
Plots showing behavior of the system. **A,** Time traces of two stochastic simulations (done with StochPy) comparing the number of P2’s released when ligand and substrate are present (blue) versus substrate only (red), where [S] = [L] = 100 *μ*M. **B,** Ligand activation (*A*) plotted over a range of substrate concentrations. Because of no, or low activity for simulations done without ligand $\left(v_{\text{noL}}\right)$ at low [S] (< 0.1 *μ*M), the error in A is too large, and these data are left out. **C,** Turnover rates $\left(v\right)$ for the most relevant cycles plotted over a range of substrate concentrations. The total rate (black; $v_{\text{total}}$) is the sum of the separated pathways (in color). The target cycle (i, blue; $v_{\text{target}}$) peaks around () and then is overtaken by steric displacements (ii, orange) until the system saturates at high [S]. **D,** Efficiency (E) plotted over a range of substrate concentrations. Efficiency is highest where incidence of the target cycle is highest (compare with **C**, above).

Substrate turnover was measured by tracking the release of P2 from the enzyme. Although we compare the turnover rate across different pathways in the main analysis, the initial aim was to confirm that the presence of ligand stimulated (increased) the rate of turnover, consistent with the operation of a myosin monomer. Simulations performed with and without ligand showed that ligand stimulates the turnover of substrate (Fig. 3A; and Movie 3, SI 1.6). The stimulatory behavior, which is named *ligand activation (A)*, can be quantified:

(3) $$A=\frac{v_{\text{yesL}}}{v_{\text{noL}}}$$

where $v_{\text{yesL}}$ is the turnover rate with ligand and $v_{\text{noL}}$ is the turnover rate without ligand. Measured over a range of substrate concentrations, ligand activation peaked at lower substrate concentrations and tended to zero at high substrate concentrations (Fig. 3B). The shape of the curve follows from the individual contributions of $v_{\text{yesL}}$ and $v_{\text{noL}}$ (inset of Fig. 3B). The turnover rate with ligand $\left(v_{\text{yesL}}\right)$ peaks and then tends toward zero at the highest concentration of substrate, and the turnover rate without ligand $\left(v_{\text{noL}}\right)$ increases and begins to saturate at high [S].

To verify that ligand activation resulted from incidence of the target cycle, we wrote routines to track each P2 released and bin them by path (Fig. 3C). The binned data shows that incidence of the target cycle (Movies 1 & 2) dominated in the same concentration range that ligand activation was highest. At higher concentrations of substrate, steric displacements dominated. Steric displacements dominate at high substrate concentrations because substrate binds more rapidly at high concentrations, allowing substrate to saturate node a after P1 dissociates. When node a saturates with substrate, additional substrate is more likely to bind node b when P2 dissociates from node b and is thus more likely to displace P2 (Movie 4, see SI 1.6). In contrast to steric displacements, incidence of idling (Movie 5, see SI 1.6) remained low over the entire range of substrate concentrations tested. Other futile pathways occurred at an even lower incidence and thus are not visible in Fig. 3C, but they do minimally contribute to the overall turnover count (see SI 1.7).

At the highest concentrations of substrate, all enzymatic activity tended toward zero. The mechanism by which this occurs is inhibitory saturation, where a complex containing multiple substrates and one ligand is repeatedly formed (Movie 6, see SI 1.6). Inhibitory saturation is explained in detail in SI, but briefly, it takes place when substrate binds to the enzyme faster than bound ligand dissociates from its nodes. In simulations performed without ligand, substrate saturation alone did not cause inhibition (Fig. 3B inset, $v_{\text{noL}}$). Although we did not test it, we believe that inhibitory saturation would be eliminated if the reaction network was restricted to states in which only one substrate is bound at a time.

Finally, the efficiency (E) of the target cycle was quantified:

(4) $$E=100\times \frac{v_{\text{target}}}{v_{\text{yesL}}}$$

where $v_{\text{target}}$ is the turnover rate of the target cycle. Higher efficiency overlaps with occurrences of the target cycle and higher ligand activation (Fig. 3D).

### Siloing simulations

**Fig. 4.**
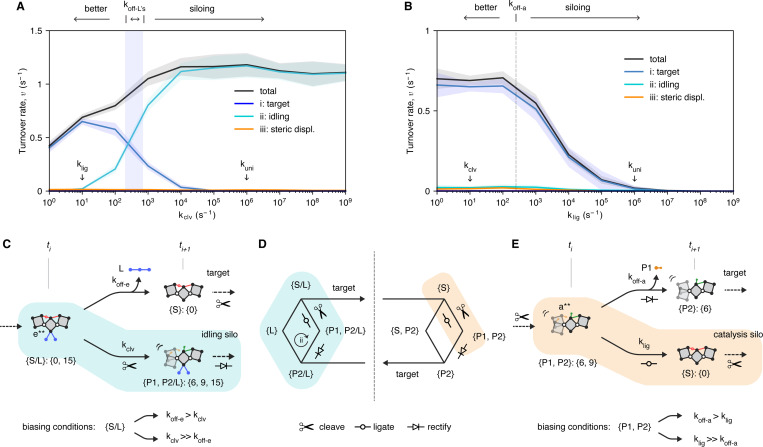
Idling and catalysis siloing. **A,** Plot of idling silo. While keeping the ligation rate, ${\text{k}}_{\text{lig}}$, constant at 10 s^−1^, idling becomes the dominant pathway taken by the system as the cleavage rate, ${\text{k}}_{\text{clv}}$, is increased. The crossover point lies where ${\text{k}}_{\text{clv}}$ surpasses the ligand’s off rates (between 10^2^ and 10^3^ s^−1^; vertical blue band). Before this point, in particular where ${\text{k}}_{\text{clv}}$ equals ${\text{k}}_{\text{lig}}$ (at 10s^−1^), the behavior is optimal, and the target cycle dominates. **B,** Plot of catalysis silo. While keeping the cleavage rate constant at 10s^−1^, the turnover rate decreases and approaches zero as the ligation rate, ${\text{k}}_{\text{lig}}$, approaches the intramolecular binding rate, ${\text{k}}_{\text{uni}}$. **C,** Idling silo pathway. Two time points along the target trajectory (top) versus idling silo trajectory (bottom) are shown. Starting at {S/L}:{0, 15} at $t_{i}$ (in blue circle), siloing dominates when ${\text{k}}_{\text{clv}}$ is much greater than the ligand off rates (here, ${\text{k}}_{\text{off-e}}$), which biases cleavage to take place before L dissociates, and a transition to {P1, P2/L}: {6, 9, 15} at $t_{i+1}$ (see SI Fig. 7N for full sequence of idling). By contrast, when ${\text{k}}_{\text{off-e}}$ is greater than ${\text{k}}_{\text{clv}}$, L is biased to dissociate before cleavage (top path), and the system transition to {S}:{0} instead, and stays on the target path. The rate conditions governing the top vs bottom paths are shown below. **D,** Left, Idling silo on the mini-network. Right, Catalysis silo on the mini-network. The starting state for each silo is circled: {S/L} for idling; and {P1, P2} for catalysis. Unlike the idling silo, the catalysis silo is not a cycle, but rather interconversion between {S} and {P1, P2} without turnover. **E,** Catalysis silo pathway. Two time points along the target trajectory (top) versus catalysis silo (bottom) are shown. Starting at {P1, P2}: {6, 9}, siloing dominates when ${\text{k}}_{\text{lig}}$ is much greater ${\text{k}}_{\text{off-a}}$, allowing ligation (bottom path) to take place before P1 dissociates (top path). The rate conditions governing the top vs bottom paths are shown below.

In this last set of results, we discuss simulations that justify the values selected for cleavage and ligation and that help to clarify the role that catalysis plays in the system. These simulations involved varying the rates for cleavage or ligation while keeping the other parameters the same (Fig. 4).

Varying the cleavage rate showed how the cleavage reaction controls entry into the idling pathway (Fig. 4A). Faster cleavage increases the overall turnover rate but has the negative effect of funneling turnover events into the idling cycle (Fig. 4A). This funneling, or siloing, increases as the cleavage rate exceeds the off rates for the ligand’s nodes. In this regime, the cleavage of substrate is biased to take place before the displacement of ligand by substrate completes, which biases idling over the target cycle (Figs. 4A, right; and 4B, bottom path in blue bubble, between $t_{i}$ and $t_{i+1}$). Slower cleavage, conversely, decreases the overall turnover rate but has the positive effect of increasing the efficiency of the target cycle by biasing ligand displacement to complete before cleavage takes place (Figs. 4A, left; and 4B, top path between $t_{i}$ and $t_{i+1}$). The ideal range lies where the cleavage rate is slower than that of the ligand ${\text{k}}_{\text{off}}$’s, but not too slow.

Varying the ligation rate while keeping the cleavage rate at an ideal constant showed that ligation controls when the substrate-bound enzyme can escape from reversible catalysis. No escape means that rectification cannot occur, and thus turnover cannot occur (Fig. 4C). Rectification, or dissociation of P1, after cleavage, prevents substrate from reforming by ligation and thus liberates a degree of freedom for ligand binding. Slow ligation biases rectification by giving P1 more time to dissociate after cleavage and maximizes entry into the target cycle (Fig. 4C, left; and 4B, top path between $t_{i+2}$ and $t_{i+3}$). Faster ligation, by contrast, inhibits enzymatic turnover and eventually stops it altogether (Fig. 4C, far right).

The inhibition of turnover begins as the ligation rate approaches and then exceeds ${\text{k}}_{\text{off-a}}$, the rate at which P1 dissociates (Fig. 4C, middle region). At this point, substrate begins to reform faster, through ligation, than P1 can dissociate. Beyond this threshold, the system becomes increasingly trseed, or siloed, in a stable but catalytically active complex of product and substrate that cannot be dismantled by ligand binding and that permanently sequesters the enzyme’s degrees of freedom (Fig. 4B, bottom path in orange bubble, between $t_{i+2}$ and $t_{i+3}$). In Fig. 4D, the graph representation is used to highlight states populated by siloing from fast ligation (orange) and those populated by siloing from fast cleavage (blue).

Because cleavage transiently liberates one degree of freedom for ligand binding and dissociation of P1 rectifies this liberation by preventing ligation, the rate of P1 dissociation, ${\text{k}}_{\text{off-a}}$, is a good upper bound (speed limit) for the rate of ligation, meaning that ligation should be slower than ${\text{k}}_{\text{off-a}}$ so that rectification can take place before ligation. However, in a system in which P1 does not dissociate spontaneously and must be displaced as is P2, we believe that the intramolecular binding rate, ${\text{k}}_{\text{uni}}$, is a more fundamental speed limit that the ligation rate must be well below. ${\text{k}}_{\text{uni}}$ is the speed limit for ligation in this hypothetical system because ligand binding will have to stop ligation, either by displacing a product molecule or by first directly competing for the mechanical degree of freedom that cleavage releases. For one of these two things to take place, ligation should not outcompete the ligand’s ability to bind internally, or ${\text{k}}_{\text{lig}}$ should be less than ${\text{k}}_{\text{uni}}$.

## Discussion

### The purpose of catalysis

Due to the design of the linkage system, splitting substrate into two pieces has the same geometric effect on the substrate-enzyme complex as substrate dissociating from a node, which is to introduce flexibility. Based on this mechanical equivalence, we defined the hydrolysis mimic. The hydrolysis mimic allows substrate to be split (cleaved) or joined (ligated) back together at a special “catalytic” node, unique to substrate. The uniqueness allows cleavage to introduce flexibility into the substrate-enzyme complex via a different path and timescale (or “off” rate) than that of normal bond dissociation. In reverse, ligation can remove the introduced flexibility with a different rate than the intramolecular binding rate $\left({\text{k}}_{\text{uni}}\right)$, and thus determine the duration of the flexibility. In this way, substrate binding can be made weaker than ligand binding through catalysis without destroying substrate’s ability to displace ligand.

By varying the rates of catalysis, we showed that when cleavage was too fast (flexibility was introduced too soon), a single ligand remained bound through many turnover events. Conversely, when ligation was too fast (flexibility was introduced for too short a time), a single substrate remained bound and inhibited turnover. Only when the flexibility introduced by catalysis existed for the right amount of time did the system oscillate between substrate and ligand. Hence, catalysis (bond splitting), as interpreted here, provides a pathway by which a tight-binding fuel can cede control of a single mechanical degree of freedom in the enzyme complex to its weaker-binding ligand or allosteric partner. This pathway must operate in parallel to normal stochastic binding and dissociation but on a slower timescale.

### Relevance to engineering molecular motors

The chain-like linkage geometry used here is simple compared to the complex three-dimensional geometries of proteins and ribonucleoproteins. Hence, only a small space of allosteric behavior is explored, which is negative allosteric cooperativity between two binding sites. Even in longer versions of these chains, events occurring at any single link in the chain can only affect adjacent links, similar to how DNA strand displacement progressively takes place [50, 51]. This attribute places limitations on the complexity of allosteric signaling that can be achieved beyond that between two sites, which is a large space [56–58].

For example, what kind of linkage could incorporate a lever-arm-like structure that moves in response to the sequential displacement of two product molecules? In myosin, actin binding triggers the release of both products, and both releases are coupled to the motion of the lever arm. How could a third binding site be incorporated into a linkage that positively or negatively affected allosteric coupling between two other sites? How can self-binding be modeled? In this system, all interactions are between the enzyme and external reactants, whereas an ATPase like kinesin also self-binds with its neck-linker domain to modulate external specificity to ATP and microtubule.

Designing more complex allosteric behaviors probably requires extending the model to two- or three-dimensional networks [12–14, 59, 60] or origami-inspired structures [61, 62]. These systems can have more independent degrees of freedom and more ways to arrange mechanical coupling between them. However, rational design of complex mechanical networks is a challenge. Most previous studies begin with networks that are algorithmically pruned to send signals between target sites. While powerful, extracting design principles from algorithmic solutions is not easy. Whether future work on mechanical network models of molecules involves rational design, algorithmic tuning, or some combination of strategies, it seems important to incorporate mechanisms that allow transduction. In this context, transduction means mechanisms that allow internal signals to be converted into external signals, and vice versa. Here, we showed that simple mechanical networks can transduce signals in ways that mimic chemistry. To do so, we assumed that conformational changes can be made without strain and can be stochastically controlled by the stepwise addition and removal of bars in the network.

A major challenge in studying biomolecular machines is determining which structures are essential for the functions they demonstrate and which may be nonessential [63–65]. Developing allosteric models that can produce the internal steps required to achieve various intermolecular outcomes should make it easier to emulate various biomolecular machines through interpolation, without the requirement of knowing every detail of how the machines may operate. Such allosteric models will also make it easier to use known experimental methods, like DNA or RNA nanotechnology [66–71], rotaxane/catenane chemistry [72], or protein engineering [17, 19, 73], to construct more capable synthetic molecular machines and motors. The linkage model is intended to be a contribution towards creating such methodologies.

## Supplementary Material

Supplement 1

## Figures and Tables

**Table 1:** Rates used for the simulations. These rates are represented in Fig. 2. ${\text{k}}_{\text{reactant-on}}$ is either ${\text{k}}_{\text{Son}}$, ${\text{k}}_{\text{Lon}}$, ${\text{k}}_{\text{P}1\text{on}}$, or ${\text{k}}_{\text{P}2\text{on}}$.

rate	value (s^−1^)	reaction type
${\text{k}}_{\text{reactant-on}}$	various	I
${\text{k}}_{\text{off-a}}$	250	III, or IV
${\text{k}}_{\text{off-b}}$	3	III, or IV
${\text{k}}_{\text{off-c}}$	680	III, or IV
${\text{k}}_{\text{off-d}}$	680	III, or IV
${\text{k}}_{\text{off-e}}$	200	III, or IV
${\text{k}}_{\text{off-f}}$	680	III, or IV
${\text{k}}_{\text{uni}}$	1 × 10^6^	II
${\text{k}}_{\text{clv}}$	10	V
${\text{k}}_{\text{lig}}$	10	VI

## Data Availability

The movies and trajectory data are available at https://doi.org/10.5281/zenodo.17969328.
