# Supplementary material for: Design of a minimal, allosteric, and ATPase-like machine using mechanical linkages
Source: ArXiv. 2025 Dec 19:arXiv:2512.17597v1. Preprint. [Version 1] (PMC12723784)
Supplement: Supplement 1 [file NIHPP2512.17597v1-supplement-1.pdf]

Supplemental

1.1 Defining a generic ATPase cycle

In this study, we define a chemical cycle that is based on the cycle of a myosin and dynein monomer. Below, we first give two reasons for why myosin and dynein were chosen as starting points and then describe how the simplified cyle is derived from the myosin and dynein cycles.

Why choose to emulate myosin and dynein monomer cycles?

The first reason is that myosin and dynein monomers can be viewed as having the same chemical cycle if their allosteric effectors—actin for myosin, and microtubule for dynein—are equated to one another by function and order of reactivity. Actin and microtubule both function as nucleotide release factors, and bind in the same order in their respective cycles. If actin and microtubule are given the same generic name, the myosin cycle (SI Eq. 5) and dynein cycle (SI Eq. 6) become equivalent statements (SI Eq. 7). This generic chemical cycle can thus define a fundamental form of ATPase behavior—behavior that can be satisfied by different structures that bind different effectors.

The second reason is that the chemical behavior defined by myosin and dynein monomers is simple compared to that of other ATPases, even kinesin. In kinesin—the third type of cytoskeletal motor—specificity to nucleotide and microtubule is also conferred by the state of the neck linker. The neck linker connects two monomers together and essentially acts as a third category of reactant. By contrast, myosin and dynein have two categories of reactants, which are nucleotide (ATP, Pi and ADP), and nucleotide release factor (effector).

Defining the simplified cycle.

The chemical cycle of myosin monomer is a series of six states [1, 2]. When thinking about the chemical cycle abstractly, it is important to keep in mind that all structural and mechanical features are forgotten, which is difficult to do given all that is currently known about myosin. In the chemical cycle, a myosin monomer is essentially an allosteric enzyme that has two binding sites, one which binds nucleotide, and one which binds actin:

$$\overbrace{\{\text{actin}\} \xrightarrow{1} \{\text{ATP/actin}\} \xrightarrow{2} \{\text{ATP}\}}^{\text{ATP displaces actin}} \xrightarrow{3} \{\text{Pi, ADP}\} \xrightarrow{4} \overbrace{\{\text{Pi, ADP/actin}\} \xrightarrow{5} \{\text{ADP/actin}\} \xrightarrow{6} \{\text{actin}\}}^{\text{actin displaces Pi, then ADP}} \dashrightarrow \tag{5}$$

Starting with the actin bound state, this cycle says that ATP binds (1) and displaces actin (2), atp is cleaved (3), actin rebinds (4) and displaces Pi (5), and then ADP (6). A dynein monomer follows the same sequence, except that actin is replaced with microtubule ('MT'):

$$\overbrace{\{\text{MT}\} \xrightarrow{1} \{\text{ATP/MT}\} \xrightarrow{2} \{\text{ATP}\}}^{\text{ATP displaces MT}} \xrightarrow{3} \{\text{Pi, ADP}\} \xrightarrow{4} \overbrace{\{\text{Pi, ADP/MT}\} \xrightarrow{5} \{\text{ADP/MT}\} \xrightarrow{6} \{\text{MT}\}}^{\text{MT displaces Pi, then ADP}} \dashrightarrow \tag{6}$$

We formally equate and abstract these two cycles by renaming the reactants:

i. actin = microtubule = ligand (L)

ii. ATP = substrate (S)

iii. Pi = product 1 (P1)

iv. ADP = product 2 (P2)

With these changes, both cycles can be represented by the following cycle:

$$\overbrace{\{\text{L}\} \xrightarrow{1} \{\text{S/L}\} \xrightarrow{2} \{\text{S}\}}^{\text{S displaces L}} \xrightarrow{3} \{\text{P1, P2}\} \xrightarrow{4} \overbrace{\{\text{P1, P2/L}\} \xrightarrow{5} \{\text{P2/L}\} \xrightarrow{6} \{\text{L}\}}^{\text{L displaces P1, then P2}} \dashrightarrow \tag{7}$$

In the final simplification of the cycle, P1 is allowed to dissociate spontaneously, and thus ligand binding is responsible only for the displacement of P2, where it is assumed that P1 spontaneously dissociates before P2 is displaced:

$$\overbrace{\{L\} \xrightarrow{1} \{S/L\} \xrightarrow{2} \{S\}}^{\text{S displaces L}} \xrightarrow{3} \underbrace{\{P1, P2\} \xrightarrow{4} \{P2\}}_{\text{P1 dissoc. spontaneously}} \overbrace{\xrightarrow{5} \{P2/L\} \xrightarrow{6} \{L\}}^{\text{L displaces P2}} \dashrightarrow \tag{8}$$

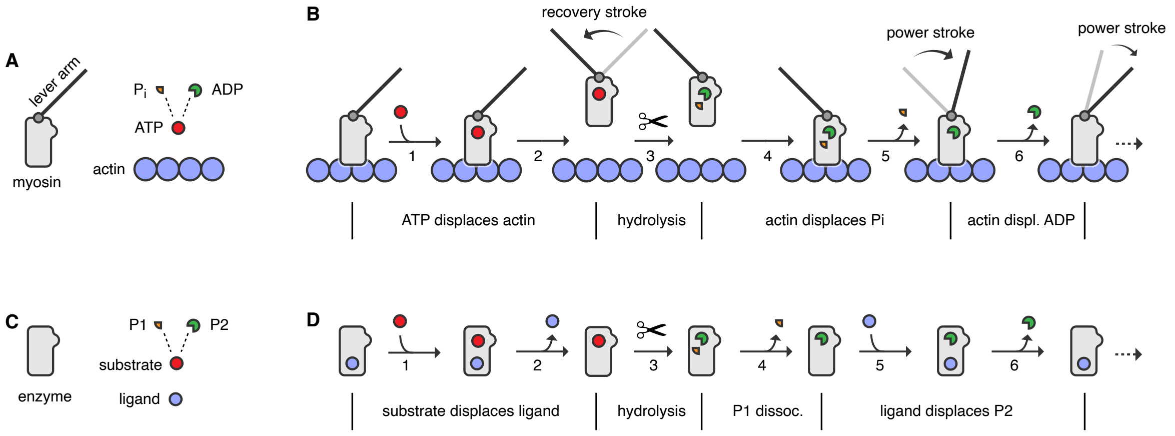

Fig. 1. Myosin chemomechanical cyle vs simplified chemical cycle. A. Five reactants of a myosin monomer: myosin with a lever arm; polymeric actin; ATP; Pi; and ADP. **B.** Chemomechanical cycle of a myosin monomer. Myosin goes through six chemical steps and two major mechanical changes of the lever arm, which are the recovery stroke, and the power stroke, where the power stroke is often depicted taking place in two stages. **C.** Five reactants of the generic ATPase-like machine: the enzyme; the ligand; substrate; P1; and P2. **D.** Simplified chemical cycle of the generic ATPase-like machine. In this cycle, P1 (the Pi analog) dissociates before ligand (the actin analog) binds.

1.2 Constructing the reaction networks

The reaction networks were constructed in these four steps:

1. Generate basis states.
2. Map exclusion rules between basis states.
3. Generate complete set of states.
4. Connect states to one another.

Each of these four steps are described in detail below:

1. Generate basis states.

The basis states were created by enumerating all the ways the single reactants can bind to the enzyme. There are seventeen basis states including the empty state (SI Fig. 2). The TRV basis states excludes two geometrically possible states by invoking an ad hoc principle, named the *adjacency rule*, that only adjacent multivalent bonds can form (SI Fig. 3).

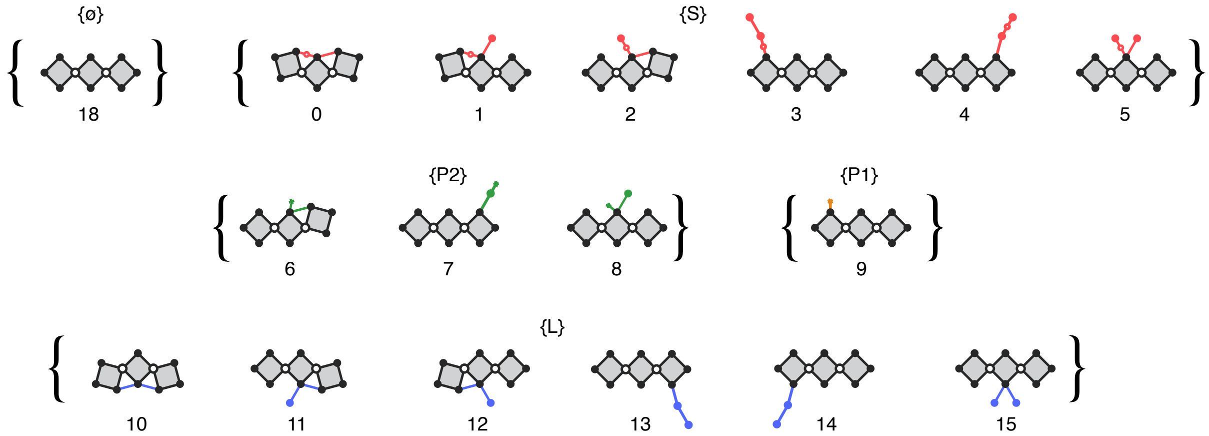

Fig. 2. Basis states. Subset of seventeen states that are used to generate and name the complete set of 449 states. In each of these states only one molecule of S, P1, P2 or L is bound, and the subset of states enumerates all the different ways these four molecules can bind to the enzyme, barring the two states eliminated by the adjacency rule.

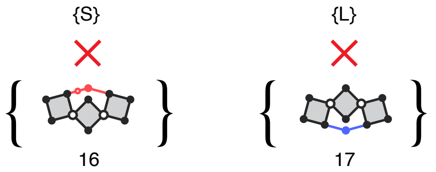

Fig. 3. Adjacency rule. Only adjacent bonds are allowed to form or dissociate between two interacting linkages. Left, disallowed substrate state in which the two outer nodes are bound, and the central node is dissociated. Right, disallowed ligand state in which the two outer nodes are bound, and the central node is dissociated.

2. Map exclusion rules between basis states.

The exclusion rules for each system are mapped out as a binary matrix that matches the three sets of substrate and product basis states against the set of ligand binding basis states. For each matchup, the two states can either coexist together on the enzyme, and thus are an allowed two-molecule-bound state, or they cannot coexist together on the enzyme, and thus are an excluded two-molecule-bound state (symbolized by). What should be noticed is that excluded states only result from matching one multivalent state to another multivalent state. The system has nine excluded states: seven for S and L ({1, 12}, {0, 12}, {1, 10}, {0, 10}, {2, 10}, {0, 11}, and {2, 11}); and two for P2 and L ({6, 10} and {6, 11}).

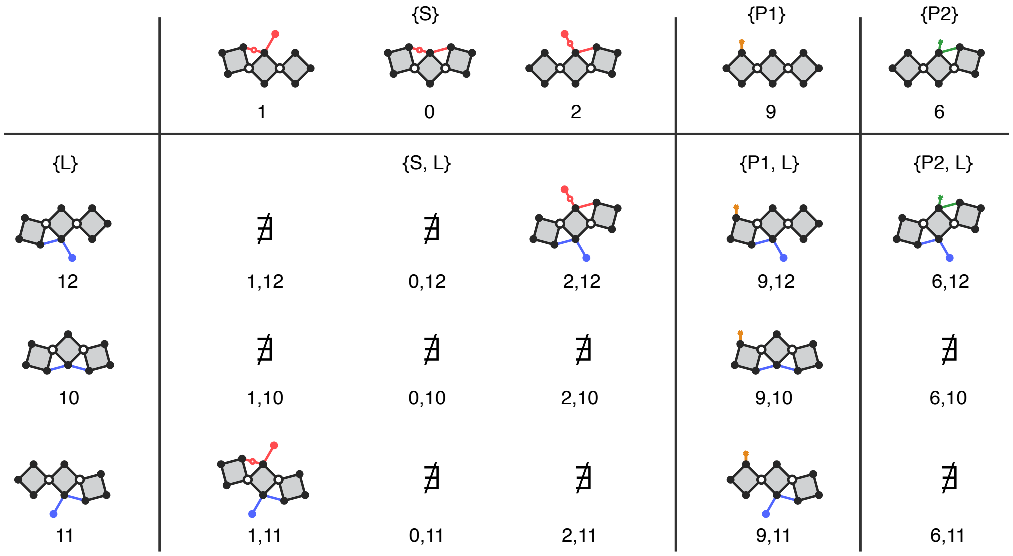

Fig. 4. Rules matrix. This binary matrix graphically displays the geometric restrictions that exist between the basis states, and which each represent an instance of negative allosteric coupling. Basis states that cannot coexist, and generate a new state, are represented with $\nexists$, for "does not exist". There are nine conflicts denoted with $\nexists$. Note that P1 and the ligand-bound-states ({L}) do have any conflicts, because P1 is bound monovalently to the enzyme.

3. Generate complete set of states.

[Notations used: B^n, where $n = 0$ to 6, denotes sets of states with n number of molecules bound to the enzyme. For example, B^0 is the empty state, and B^1 is the set of one-molecule-bound basis states (see SI Fig. 2). $\mathbf{B}^n$, for $n > 1$, is the matrix form of each set of bound states, and $\mathbf{B}^n_m$, refers to the row of the matrix, where the subscript m denotes a state written as a set of basis states (e.g. if $m = 0$, 13, this means state $\{0, 13\}$). In the matrix, '0' means the states cannot coexist, and '1' means that can coexist. Hence, '0' is equivalent to the $\nexists$ symbol used in Fig. 4.]

Sets of higher order bound states were successively generated by multiplying (as a binary matrix) the set of states with one molecule less, with the set of basis states B^1 (e.g. $B^4 = B^3 \times B^1$). The process starts with the generation of B^2, where $B^2 = B^1 \times B^1$:

$\mathbf{B^2} =$

	0	1	2	3	4	5	6	7	8	9	10	11	12	13	14	15
0	0	0	0	0	0	0	0	0	0	0	0	0	0	1	1	1
1	0	0	0	0	1	0	0	1	0	0	0	1	0	1	1	1
2	0	0	0	1	0	0	0	0	0	1	0	0	1	1	1	1
3	0	0	1	0	1	1	1	1	1	0	1	1	1	1	1	1
4	0	1	0	1	0	1	0	0	1	1	1	1	1	1	1	1
5	0	0	0	1	1	0	0	1	0	1	1	1	1	1	1	1
6	0	0	0	1	0	0	0	0	0	1	0	0	1	1	1	1
7	0	1	0	1	0	1	0	0	1	1	1	1	1	1	1	1
8	0	0	0	1	1	0	0	1	0	1	1	1	1	1	1	1
9	0	0	1	0	1	1	1	1	1	0	1	1	1	1	1	1
10	0	0	0	1	1	1	0	1	1	1	0	0	0	0	0	0
11	0	1	0	1	1	1	0	1	1	1	0	0	0	0	1	0
12	0	0	1	1	1	1	1	1	1	1	0	0	0	1	0	0
13	1	1	1	1	1	1	1	1	1	1	0	0	1	0	1	1
14	1	1	1	1	1	1	1	1	1	1	0	1	0	1	0	1
15	1	1	1	1	1	1	1	1	1	1	0	0	0	1	1	0

The resulting $\mathbf{B}^2$ matrix has two uses: 1) it generates the two-molecule-bound states, which are indicated wherever a '1' appears in the matrix; and 2) it generates a set of row vectors $\mathbf{B}^2_m$ to compliment each basis state m in B^1, which are in turn used as multipliers to generate the higher order states. For example, the '1' in row 0, column 13, represents

state {0, 13} in B^2. To generate B^3 ($B^3 = B^2 \times B^1$), each state in B^2 is converted into a row vector for $\mathbf{B}^3$, using the set of basis vectors $\mathbf{B}^2_m$. This is done by performing an element wise multiplication of the basis vectors that comprise each state in B^2. Hence, state {0, 13} is converted into row vector $\mathbf{B}^3_{0,13}$ in $\mathbf{B}^3$ by multiplying basis vectors $\mathbf{B}^2_0$ and $\mathbf{B}^2_{13}$ together element-wise, which is expressed as the Hadamard product, where '$\circ$' means element-wise multiplication:

$$\begin{aligned}\mathbf{B}^3_{0,13} = \mathbf{B}^2_0 \circ \mathbf{B}^2_{13} = &[\ 0\ 0\ 0\ 0\ 0\ 0\ 0\ 0\ 0\ 0\ 0\ 0\ 0\ 1\ 1\ 1\ ] \circ \\ &[\ 1\ 1\ 1\ 1\ 1\ 1\ 1\ 1\ 1\ 1\ 0\ 0\ 1\ 0\ 1\ 1\ ] = \\ &[\ 0\ 0\ 0\ 0\ 0\ 0\ 0\ 0\ 0\ 0\ 0\ 0\ 0\ 0\ 1\ 1\ ]\end{aligned}$$

Thus, for every '1' in $\mathbf{B}^2$, the Hadamard product is used to construct the rows of $\mathbf{B}^3$:

$\mathbf{B^3} =$

	0	1	2	3	4	5	6	7	8	9	10	11	12	13	14	15
0, 13	0	0	0	0	0	0	0	0	0	0	0	0	0	0	1	1
0, 14	0	0	0	0	0	0	0	0	0	0	0	0	0	1	0	1
0, 15	0	0	0	0	1	0	0	0	0	0	0	0	0	1	1	0
1, 4	0	0	0	0	0	0	0	0	0	0	0	1	0	1	1	1
1, 7	0	0	0	0	0	0	0	0	0	0	0	1	0	1	1	1
1, 11	0	0	0	0	1	0	0	1	0	0	0	0	0	0	1	0
⋮	⋮	⋮	⋮	⋮	⋮	⋮	⋮	⋮	⋮	⋮	⋮	⋮	⋮	⋮	⋮	⋮
14, 15	1	1	1	1	1	1	1	1	1	1	0	0	0	1	0	0

To generate $\mathbf{B}^4$ the process is repeated. For example, the row vector $\mathbf{B}^4_{0,13,14}$ is generated by the product of $\mathbf{B}^3_{0,13}$ and basis vector $\mathbf{B}^2_{14}$:

$$\begin{aligned}\mathbf{B}^4_{0,13,14} = \mathbf{B}^3_{0,13} \circ \mathbf{B}^1_{14} = &[\ 0\ 0\ 0\ 0\ 0\ 0\ 0\ 0\ 0\ 0\ 0\ 0\ 0\ 0\ 1\ 1\ ] \circ \\ &[\ 1\ 1\ 1\ 1\ 1\ 1\ 1\ 1\ 1\ 1\ 0\ 1\ 0\ 1\ 0\ 1\ ] = \\ &[\ 0\ 0\ 0\ 0\ 0\ 0\ 0\ 0\ 0\ 0\ 0\ 0\ 0\ 0\ 0\ 1\ ]\end{aligned}$$

If $\mathcal{B}$ is used to symbolize the full set of states in the system, then $\mathcal{B} = \{B^0, B^1, B^2, B^3, B^4, B^5, B^6\}$, where B^0 is the empty state, in which none of the nodes are occupied, and B^6 is the subset in which all six nodes are occupied by a different reactant. The number of states in each set is given below in Table 2. The total list of states is given in....

Set	No. of states
B^0	1
B^1	16
B^2	74
B^3	150
B^4	142
B^5	58
B^6	8
$\mathcal{B}$ (total)	449

Table 2: **Number of states in each set.**

4. Connect states to one another

The states were connected together in two stages: (i) a unique set was constructed in which each member was a pair of states that could reversibly transition to one another; and (ii) each direction of the pair was again classified by reaction type and assigned with the appropriate rate. The process has some redundancy because pairs were classified by reaction type at both stages. In principle, this could have been done once. First we describe stage i in more detail, and then stage ii:

i, Constructing a unique set of transition pairs: This process was done in three parts: (a) finding pairs that define bimolecular binding/dissociation; (b) finding pairs that define intramolecular binding/dissociation; and (c) finding pairs that define catalysis/ligation. These three categories are described individually below:

a, Finding bimolecular binding/dissociation pairs: These pairs were found using a matrix called the binding matrix **b**, which describes whether two basis states in the B^1 set can combine to form a new state by a binding reaction, with the addition of a row for the empty state ($\{18\}$ or B^0):

$$\mathbf{b} = \begin{array}{c|cccccccccccccccc} & 0 & 1 & 2 & 3 & 4 & 5 & 6 & 7 & 8 & 9 & 10 & 11 & 12 & 13 & 14 & 15 \\ \hline 0 & 0 & 0 & 0 & 0 & 0 & 0 & 0 & 0 & 0 & 0 & 0 & 0 & 0 & 1 & 1 & 1 \\ 1 & 0 & 0 & 0 & 0 & 1 & 0 & 0 & 1 & 0 & 0 & 0 & 0 & 0 & 1 & 1 & 1 \\ 2 & 0 & 0 & 0 & 1 & 0 & 0 & 0 & 0 & 0 & 1 & 0 & 0 & 0 & 1 & 1 & 1 \\ 3 & 0 & 0 & 0 & 0 & 1 & 1 & 0 & 1 & 1 & 0 & 0 & 0 & 0 & 1 & 1 & 1 \\ 4 & 0 & 0 & 0 & 1 & 0 & 1 & 0 & 0 & 1 & 1 & 0 & 0 & 0 & 1 & 1 & 1 \\ 5 & 0 & 0 & 0 & 1 & 1 & 0 & 0 & 1 & 0 & 1 & 0 & 0 & 0 & 1 & 1 & 1 \\ 6 & 0 & 0 & 0 & 1 & 0 & 0 & 0 & 0 & 0 & 1 & 0 & 0 & 0 & 1 & 1 & 1 \\ 7 & 0 & 0 & 0 & 1 & 0 & 1 & 0 & 0 & 1 & 1 & 0 & 0 & 0 & 1 & 1 & 1 \\ 8 & 0 & 0 & 0 & 1 & 1 & 0 & 0 & 1 & 0 & 1 & 0 & 0 & 0 & 1 & 1 & 1 \\ 9 & 0 & 0 & 0 & 0 & 1 & 1 & 0 & 1 & 1 & 0 & 0 & 0 & 0 & 1 & 1 & 1 \\ 10 & 0 & 0 & 0 & 1 & 1 & 1 & 0 & 1 & 1 & 1 & 0 & 0 & 0 & 0 & 0 & 0 \\ 11 & 0 & 0 & 0 & 1 & 1 & 1 & 0 & 1 & 1 & 1 & 0 & 0 & 0 & 0 & 1 & 0 \\ 12 & 0 & 0 & 0 & 1 & 1 & 1 & 0 & 1 & 1 & 1 & 0 & 0 & 0 & 1 & 0 & 0 \\ 13 & 0 & 0 & 0 & 1 & 1 & 1 & 0 & 1 & 1 & 1 & 0 & 0 & 0 & 0 & 1 & 1 \\ 14 & 0 & 0 & 0 & 1 & 1 & 1 & 0 & 1 & 1 & 1 & 0 & 0 & 0 & 1 & 0 & 1 \\ 15 & 0 & 0 & 0 & 1 & 1 & 1 & 0 & 1 & 1 & 1 & 0 & 0 & 0 & 1 & 1 & 0 \\ 18 & 0 & 0 & 0 & 1 & 1 & 1 & 0 & 1 & 1 & 1 & 0 & 0 & 0 & 1 & 1 & 1 \end{array}$$

The matrix **b** maps out a subset of the pairs in $\mathbf{B^2}$, with entries that reflect only allowed single-node interactions. An entry of a '1' at a given column position means that the basis state at that column position can combine with the basis state for that row to form a new state that contains the row basis state and the column basis state, where the column state is the new molecule that binds at a single node. Hence, each row can be thought of as a basis vector for binding. And thus, for every state in the system, a combined binding vector could be calculated using the Hadamard product in the exact same way it was done to find successive higher order states. For example, to find the binding vector $\mathbf{b_{1,13}}$ for state {1, 13}, the binding vectors for {1} and {13} are multiplied together:

$$\begin{aligned} \mathbf{b}_{1,13} = \mathbf{b}_1 \circ \mathbf{b}_{13} = & \left[\ 0\ 0\ 0\ 0\ 1\ 0\ 0\ 1\ 0\ 0\ 0\ 0\ 0\ 1\ 1\ 1\ \right] \circ \\ & \left[\ 0\ 0\ 0\ 1\ 1\ 1\ 0\ 1\ 1\ 1\ 0\ 0\ 0\ 0\ 1\ 1\ \right] = \\ & \left[\ 0\ 0\ 0\ 0\ 1\ 0\ 0\ 1\ 0\ 0\ 0\ 0\ 0\ 0\ 1\ 1\ \right] \end{aligned}$$

As there is a '1' at positions $\{4\}, \{6\}, \{14\}$, and $\{15\}$, state $\{1, 13\}$ can make four different binding transitions to new states containing these basis states, and in reverse, dissociations take place to transition to state $\{1, 13\}$:

$$\begin{aligned} \{1, 13\} &\rightleftharpoons \{1, 4, 13\} \\ \{1, 13\} &\rightleftharpoons \{1, 6, 13\} \\ \{1, 13\} &\rightleftharpoons \{1, 13, 14\} \\ \{1, 13\} &\rightleftharpoons \{1, 13, 15\} \end{aligned} \tag{9}$$

Hence, for every state in the system, the process is repeated to find all the possible bimolecular binding/dissociation transitions.

b. Finding intramolecular binding/dissociation pairs: These pairs were found using a matrix, the isomerization matrix (**i**), that describes which states can transition to another by an intramolecular binding/dissociation reaction. For example, when the ligand or substrate are bound at two nodes, and make a third association with the enzyme, and vice versa for dissociation:

$\mathbf{i} =$

	0	1	2	3	4	5	6	7	8	9	10	11	12	13	14	15
0	0	1	1	0	0	0	0	0	0	0	0	0	0	0	0	0
1	1	0	0	1	0	1	0	0	0	0	0	0	0	0	0	0
2	1	0	0	0	1	1	0	0	0	0	0	0	0	0	0	0
3	0	1	0	0	0	0	0	0	0	0	0	0	0	0	0	0
4	0	0	1	0	0	0	0	0	0	0	0	0	0	0	0	0
5	0	1	1	0	0	0	0	0	0	0	0	0	0	0	0	0
6	0	0	0	0	0	0	0	1	1	0	0	0	0	0	0	0
7	0	0	0	0	0	0	1	0	0	0	0	0	0	0	0	0
8	0	0	0	0	0	0	1	0	0	0	0	0	0	0	0	0
9	0	0	0	0	0	0	0	0	0	0	0	0	0	0	0	0
10	0	0	0	0	0	0	0	0	0	0	0	1	1	0	0	0
11	0	0	0	0	0	0	0	0	0	0	1	0	0	1	0	1
12	0	0	0	0	0	0	0	0	0	0	1	0	0	0	1	1
13	0	0	0	0	0	0	0	0	0	0	0	1	0	0	0	0
14	0	0	0	0	0	0	0	0	0	0	0	0	1	0	0	0
15	0	0	0	0	0	0	0	0	0	0	0	1	1	0	0	0

Like $\mathbf{b}$, $\mathbf{i}$ is a subset of the pairs defined in $\mathbf{B}^2$. The possible isomerizations indicated by $\mathbf{i}$ cannot conflict with any other molecule that is bound to the enzyme, where the conflict can be either steric or allosteric. For example, the state {1, 14} does not have any conflicts, but {1, 12} does have an allosteric conflict, even though {14} can transition to {12} when it is on the enzyme alone. Hence, to account for conflicts, $\mathbf{i}$ is used with $\mathbf{B}^2$ in the following way. To test for isomerizations, each basis state in a state is tested individually by taking the Hadamard product of its $\mathbf{i}$ vector and the $\mathbf{B}^2$ basis vectors for all the other basis states in the state. For example, to test state {3, 12, 13} for isomerizations, {3}, {12}, and {13} are tested separately. Testing {3} first is done by multiplying $\mathbf{i}_3$ by $\mathbf{B}^2_{12}$ and $\mathbf{B}^2_{13}$, which shows that {3} cannot isomerize when present with {12}, and {13}; or substrate on node a cannot bind any other node when one ligand is bound divalently at d and e, and another is bound at f:

$$\begin{aligned}\mathbf{i}_3 \circ \mathbf{B}^2_{12} \circ \mathbf{B}^2_{13} = & \left[\begin{array}{cccccccccccccccc} 0 & 1 & 0 & 0 & 0 & 0 & 0 & 0 & 0 & 0 & 0 & 0 & 0 & 0 & 0 & 0 \end{array}\right] \circ \\ & \left[\begin{array}{cccccccccccccccc} 0 & 0 & 1 & 1 & 1 & 1 & 1 & 1 & 1 & 1 & 0 & 0 & 0 & 1 & 0 & 0 \end{array}\right] \circ \\ & \left[\begin{array}{cccccccccccccccc} 1 & 1 & 1 & 1 & 1 & 1 & 1 & 1 & 1 & 1 & 0 & 0 & 1 & 0 & 1 & 1 \end{array}\right] = \\ & \left[\begin{array}{cccccccccccccccc} 0 & 0 & 0 & 0 & 0 & 0 & 0 & 0 & 0 & 0 & 0 & 0 & 0 & 0 & 0 & 0 \end{array}\right]\end{aligned}$$

Testing {12}, reveals one possible isomerization to state {3, 13, 14}:

$$\begin{aligned}\mathbf{i}_{12} \circ \mathbf{B}^2_{3} \circ \mathbf{B}^2_{13} = & \left[\begin{array}{cccccccccccccccc} 0 & 0 & 0 & 0 & 0 & 0 & 0 & 0 & 0 & 0 & 1 & 0 & 0 & 0 & 1 & 1 \end{array}\right] \circ \\ & \left[\begin{array}{cccccccccccccccc} 0 & 0 & 1 & 0 & 1 & 1 & 1 & 1 & 1 & 1 & 0 & 1 & 1 & 1 & 1 & 1 \end{array}\right] \circ \\ & \left[\begin{array}{cccccccccccccccc} 1 & 1 & 1 & 1 & 1 & 1 & 1 & 1 & 1 & 1 & 0 & 0 & 1 & 0 & 1 & 1 \end{array}\right] = \\ & \left[\begin{array}{cccccccccccccccc} 0 & 0 & 0 & 0 & 0 & 0 & 0 & 0 & 0 & 0 & 0 & 0 & 0 & 0 & 1 & 1 \end{array}\right]\end{aligned}$$

where in this isomerization the divalently bound ligand is dissociating from node d or e. Testing {13} results in no other isomerizations. Hence the final result is that {3, 12, 13} isomerizes to {3, 13, 14} and {3, 13, 15}, where because basis states are ordered numerically, {14} and {15} are written after {13} in the new states, though they are isomerization of {12}:

$$\begin{aligned}\{3, 12, 13\} &\rightleftharpoons \{3, 13, 14\} \\ \{3, 12, 13\} &\rightleftharpoons \{3, 13, 15\}\end{aligned} \tag{10}$$

To describe the transitions in terms of the molecules: in the forward direction, the divalently bound ligand dissociates from node d or e, and in the reverse direction, the same ligand rebinds binds at node d or e.

c. Finding cleavage/ligation pairs: These pairs were found by looking for all states that contain {0} the fully bound substrate, and converting {0} to {6} and {9}, where {6} is P1 bound at node a, and {9} is P2 bound divalently. Any state that contains {0} automatically accounts for the reaction in both directions and rules out any geometric conflicts, because the substrate can only be fully bound when there are no geometric conflicts. Thus any state generated by converting {0} to {6} and {9} is a ligation ready state without geometric conflicts, which specifically means they do not contain basis states {11} or {12}.

ii. Assigning rates to each direction of a reaction pair: Rates were assigned using a series of logical statements that checked to see what kind of forward and reverse reactions a pair of states described, and then assigned rates accordingly: dissociations were assigned rates based on the node from which the dissociation took place, bimolecular binding reactions were assigned the bimolecular binding rate constant multiplied by a factor that reflected the concentration of the molecule binding, intramolecular binding reactions were all assigned the same unimolecular rate (k_{uni}); and cleavage and ligation reactions were assigned the same rate (k_{cat}) (see tables 5, 6, and 7, which are repeated from the main text.)

Table 3: Categories of reaction rates

Parameter	Value
Bimolecular rate constant, k_{bi}	$9 \times 10^6\,\text{M}^{-1}\text{s}^{-1}$
Concentration, c_m ($m =$ S, P1, P2, L)	varies with m; units of M
Intermolecular binding rates, k_m	$k_{\text{bi}} \times c_m$ s^{-1}
Intramolecular binding rate, k_{uni}	$1 \times 10^6\,\text{s}^{-1}$
Dissociation rates, $k_{\text{off-}node}$	varies with $node$; units of s^{-1}
Catalysis, k_{cat}	$100\,\text{s}^{-1}$

Table 4: Dissociation rates

Rate	Reactants	Value
$k_{\text{off-a}}$	S/P1	$250\,\text{s}^{-1}$
$k_{\text{off-b}}$	S/P2	$3\,\text{s}^{-1}$
$k_{\text{off-c}}$	S/P2	$680\,\text{s}^{-1}$
$k_{\text{off-d}}$	L	$680\,\text{s}^{-1}$
$k_{\text{off-e}}$	L	$200\,\text{s}^{-1}$
$k_{\text{off-f}}$	L	$680\,\text{s}^{-1}$

1.3 Stochastic formulation of the intermolecular on rates

The rates expressed in table 5 in the SI (table 1 in main text) were input as stochastic rates in the simulations (table 8 in SI). To express the rates stochastically, a scaling factor was used, which is a reaction volume multiplied by Avogadro's number ($N_A \times v$). By dividing the bimolecular rate constant (k_{bi}) by this scaling factor, k_{bi}, which has units of $\text{M}^{-1}\text{s}^{-1}$, and which retains some relevance to reaction rates for real biomolecules, was converted into a stochastic version, k^*_{bi}, with units of s^{-1}. Likewise, concentrations, c_m's, which have units of M, were multiplied by the scaling factor to convert them into their stochastic versions, c^*_m's, which have units of "number of molecules" (No.).

Table 5: Stochastic conversions of rates

Parameter	Value
Volume, v	$1 \times 10^{-12}\,\text{m}^3$
Scaling factor, $N_A \times v$	$6.022 \times 10^{11}\,\text{M}^{-1}$
Stoch. bimol. rate constant, $k^*_{\text{bi}} = k_{\text{bi}}/(N_A \times v)$	$9/6.022 \times 10^{-5}\,\text{s}^{-1}$
Stoch. "concentration", $c^*_m = c_m \times (N_A \times v)$	varies with m; units of No.
Intermolecular binding rates, k_m	$k^*_{\text{bi}} \times c^*_m$ (or $k_{\text{bi}} \times c_m$)

1.4 Converting node energies into off rates

Here, an expression is derived for calculating a node dissociation rate ($k_{\text{off-}node}$) in terms of its node energy (ε_{node}), and vice versa. This is done by satisfying local detailed balance for binding to and dissociation from a single node, as shown in the following figure:

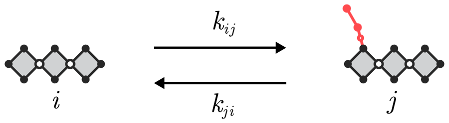

State i is the unbound enzyme, and state j is the bound enzyme; k_{ij} is the rate for transitioning from i to j, and k_{ji} is the rate for transitioning from j to i. Although a specific reaction is shown in the figure, the setup of the derivation is general and can work for any node and molecule that binds that node.

If the equilibrium probability of being in state i is written as p_i, and for state j, written as p_j, then the equilibrium condition must satisfy:

$$k_{ij}p_i = k_{ji}p_j \tag{11}$$

The equilibrium probabilities are given by the Boltzmann factors for each state, divided by the partition function:

$$p_i = \frac{e^{-\beta G_i}}{Z} \tag{12}$$

where G_i is the free energy of the state. Using the above expression for p_i and p_j in equation 3, allows the transition rates to be expressed in terms of the state energies:

$$\frac{k_{ij}}{k_{ji}} = e^{\beta(G_j - G_i)} \tag{13}$$

The goal is to replace the above free energies with expressions relevant to states of the linkage. To do this, we closely follow the modeling done in Marzen et al. [3] for a "one-site MWC molecule", except we leave out the enzyme's "activated" state and its corresponding energy:

State	Energy	Boltzmann factor
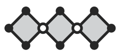	ε_l	$e^{-\beta\varepsilon_l}$
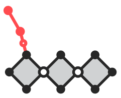	$\varepsilon_l + \varepsilon_{node} - \mu$	$e^{-\beta(\varepsilon_l + \varepsilon_{node} - \mu)}$

Table 6: Energies of the unbound and bound enzyme.

In Table 6, ε_l is the conformational energy of the enzyme (or receptor), and ε_{node} is the binding energy of the node. The full expression for μ, the chemical potential, which is described in Marzen et al. [3] as the "free energy cost of removing a ligand from dilute solution", is:

$$\mu = \mu_0 + k_B T \ln\frac{c}{c_0} \tag{14}$$

where μ_0 is an "unspecified reference chemical potential", and c_0 is an "unspecified reference concentration".

Equation 5 can be re-expressed in terms of the energies in Table 6 by letting $G_i = \varepsilon_l$ and $G_j = \varepsilon_l + \varepsilon_b - \mu$, and by using the full expression for μ in equation 6:

$$\frac{k_{ij}}{k_{ji}} = \frac{c}{c_0} e^{-\beta(\varepsilon_{node} - \mu_0)} \tag{15}$$

In the final step, k_{ij}, a binding rate, is expressed as the concentration (c) of the molecule binding, multiplied by a bimolecular rate constant (k_{bi}), so that $k_{ij} = ck_{\mathrm{bi}}$; and k_{ij}, a dissociation rate, is renamed $k_{\text{off-}node}$. Making these two substitutions and solving for $k_{\text{off-}node}$, gives:

$$k_{\text{off-}node} = c_0 k_{\mathrm{bi}}\, e^{-\beta\Delta\varepsilon_{node}} \tag{16}$$

where $\Delta\varepsilon_{node} = \mu_0 - \varepsilon_{node}$. Taking the log of both sides allows the node energy to be solved for in terms of the dissociation rate:

$$\Delta\varepsilon_{node} = -k_B T \ln\left(\frac{k_{\text{off-}node}}{c_0 k_{\text{bi}}}\right) \tag{17}$$

1.5 Behavior blocks

Here we describe the different ways that substrate turnover takes place, using twelve flow charts ("blocks"). Each block contains multiple pathways by which turnover can take place. These twelve blocks are the twelve different categories of turnover behavior that were tracked by code, analyzed and graphed. All describe a set of unique futile behaviors, except the productive cycle block, which describes the desired non-futile behavior.

Reaction sequences that make the behavior blocks

To generate the flow charts, we first defined a set of fourteen reaction sequences. Each of these sequences describes a different way that the composition of molecules bound to the enzyme changes. Eight sequences (ad2, ad2w/S, ad2*, sd, combo-I, combo-II, rd $\rightarrow \emptyset \rightarrow$ S, and rd $\rightarrow \emptyset \rightarrow$ L) describe eight different ways that P2 dissociates from the enzyme, accompanied by the binding of S, L, of a combination of S and L. Two sequences describe two variations of ligand displacement by susbtrate (ad1, ad1ws). One sequence describes how one substrate can be exchanged for another substrate (combo III). One describes cleavage. One describes rectification. And finally one describes idling:

1. ad1 (allosteric displacement 1): A single substrate binds and displaces ligand, such that when ligand dissociates, substrate is completely bound at all three nodes.
2. ad1ws (allosteric displacement 1 weak start): A single substrate binds and displaces ligand, such that when ligand dissociates, substrate is NOT completely bound at all three nodes.
3. ad2 (allosteric displacement 2): A single ligand binds and displaces P2, such that when P2 dissociates, ligand is completely bound at all three nodes.
4. ad2w/S (allosteric displacement 2 with S): Same as ad2, except that when P2 dissociates, substrate happens to be bound as well, but bound without actively participating in the displacement of P2.
5. ad2* (allosteric displacement 2*): Either a single ligand binds and displaces P2, such that the ligand is NOT completely bound when P2 dissociates; or, multiple ligand bind and displace P2, which requires that none of the ligands can be completely bound.
6. sd (steric displacement): a single subtrate binds and displaces P2.
7. combo I: Substrate and ligand displace P2 together, such that ligand is partially bound when P2 dissociates.
8. combo II: Substrate and ligand displace P2 together, such that ligand is completely bound when P2 dissociates.
9. combo III: The exchange of substrate before it is cleaved, mediated by another substrate alone, or another substrate and ligand working together. After exchange is completed, the new substrate will either displace the ligand, or the ligand will displace the new substrate. If ligand wins, it will eventually be displaced by another substrate. Thus, we say that a combo III always ends with an ad1.
10. rd $\rightarrow \emptyset \rightarrow$ S: P2 spontaneously dissociates, followed by substrate binding to the empty enzyme.
11. rd $\rightarrow \emptyset \rightarrow$ L: P2 spontaneously dissociates, followed by ligand binding to the empty enzyme.
12. ✂ (cleavage): substrate is cleaved into P1 and P2
13. —▷|— (rectification): P1 dissociates, which stops reversible catalysis
14. idling: Ligand stays bound during the cleavage of substrate. Idling may of may not include ligand also staying bound through rectification.

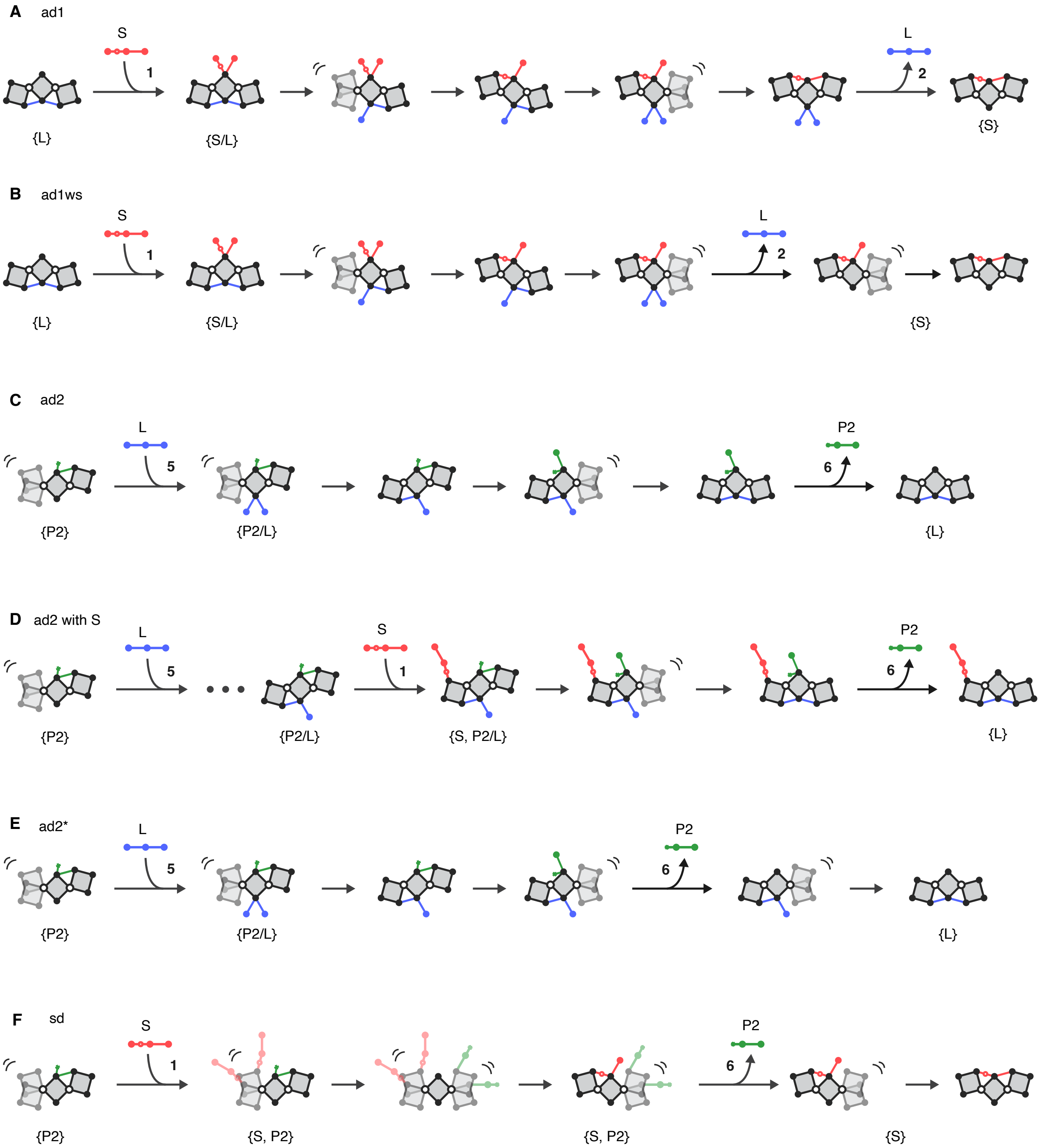

Fig. 5. Reaction sequences.

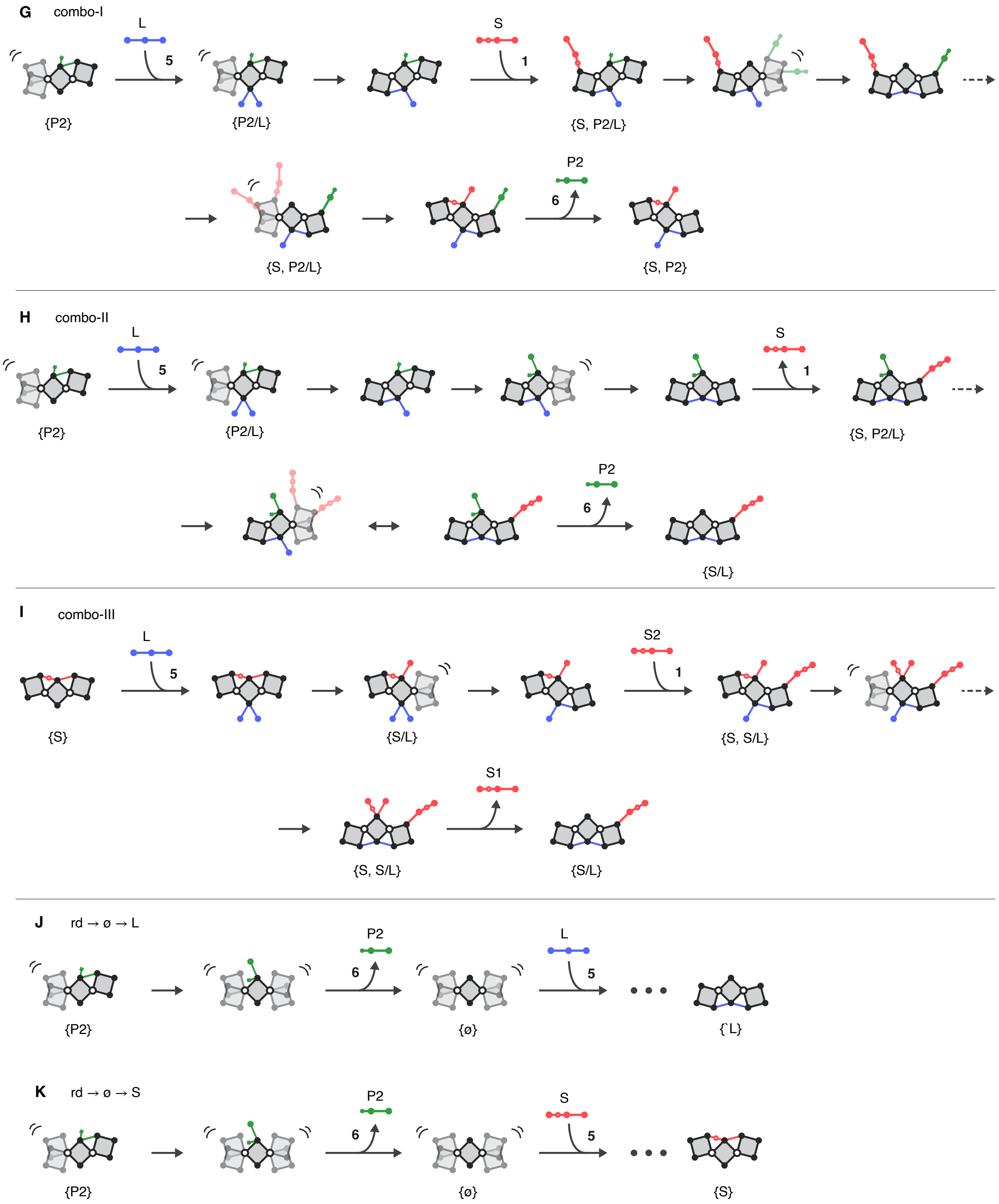

Fig. 6. Reaction sequences.

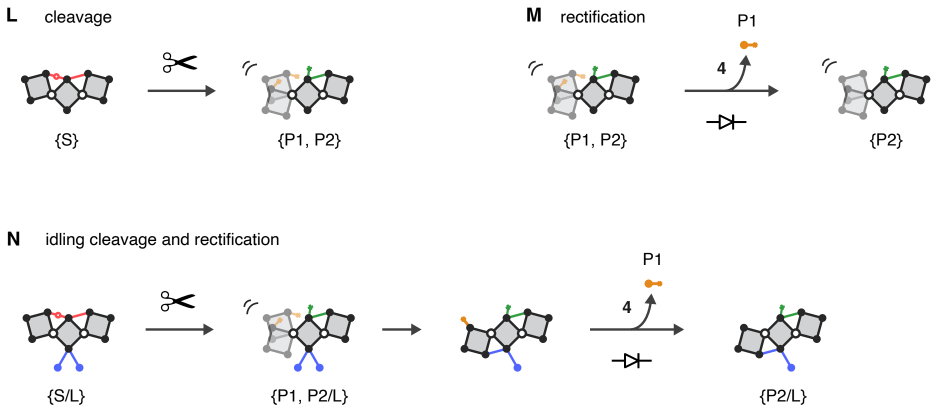

Fig. 7. Reaction sequences.

Behavior blocks

There are twelve behavior blocks that are defined from the reaction sequences (list below; and SI Figs. 8 & 9). The first four paths on the list of blocks below are the most commonly occuring. Each path begins and ends with the dissociation of P2. In between the beginning an end a substrate binds and is cleaved. The first P2 that dissociates is the P2 born from the previous substrate that was bound and cleaved. The second P2 that dissociates is born from the substrate that binds and cleaved between the two P2 dissociation events. If the two P2 dissociations conform to the definitions of a productive cycle, then the cycle is a productive cycle. If they do not, the cycle is futile:

1. pc (productive cycle): A pc begins and ends good.
2. fryS (futile-reset-yes-S): A fryS begins good (unless it is preceded by a fryS or lfsd that end with a sd); and it ends badly. Its bad ending is either the steric displacement of P2 by S (sd), or the displacement of P2 by ligand and substrate together (combo I).
3. lfsd1 (ligand-free-sd; sd at the beginning): A lfsd1 begins and ends badly. It begins with a sd, and ends in one of four futile ways (sd, combo-I, ad2*, or rd $\rightarrow \emptyset \rightarrow$ S). The 'ligand-free' designation is accurate if it does not end with a combo-I or ad2*. Since a combo-I and ad2* occur rarely, more often then not, a lfsd is 'ligand-free'.
4. fssd (futile-start-sd): A fssd begins badly but ends good. It begins with a sd, and ends like a productive cycle.
5. idling: Idling can begin and end good or bad. It is futile and unique in that cleavage takes place while ligand is bound.
6. frnS (futile-reset-no-S): A frnS begins good (unless it is preceded by a fryS or lfsd that end with a sd, just like a fryS); and it ends badly. Its bad ending is either P2 spontaneously dissociating, followed substrate binding (rd $\rightarrow \emptyset \rightarrow$ S); or the dissociation of P2 when ligand is weakly bound (ad2*).
7. frc (futile-reset-complete): A frc begins like a frnS and fryS, and ends badly, like frnS (rd $\rightarrow \emptyset \rightarrow$ S).
8. lf-frc (ligand-free-futile-reset-complete): A lf-frc begins badly, with either a sd or rd $\rightarrow \emptyset \rightarrow$ S; and it ends badly, like a frc (rd $\rightarrow \emptyset \rightarrow$ S). The 'ligand-free' designation is accurate if it does not ends with a combo-I.
9. fs (futile-start): A futile-start begins bad, and ends good (like a pc). Its bad beginning is the random dissociation of P2, followed by the binding S to the empty enzyme (rd $\rightarrow \emptyset \rightarrow$ S).
10. lfsd2 (ligand-free-sd; sd at the end): A lfsd2 begins bad (rd $\rightarrow \emptyset \rightarrow$ S), and ends bad (sd or combo-I). The "ligand-free" designation is accurate if ends with an sd.
11. lf (ligand-free): A lf begins and ends bad. Its bad beginning is rd $\rightarrow \emptyset \rightarrow$ S, and its bad ending is either ad2* or rd $\rightarrow \emptyset \rightarrow$ S. The 'ligand-free' designation is accurate if it ends with rd $\rightarrow \emptyset \rightarrow$ S.
12. ws (weak-start): A ws almost begins good (like a pc), but is characterized by a displacement sequence in which ligand dissociates when substrate is partially bound.

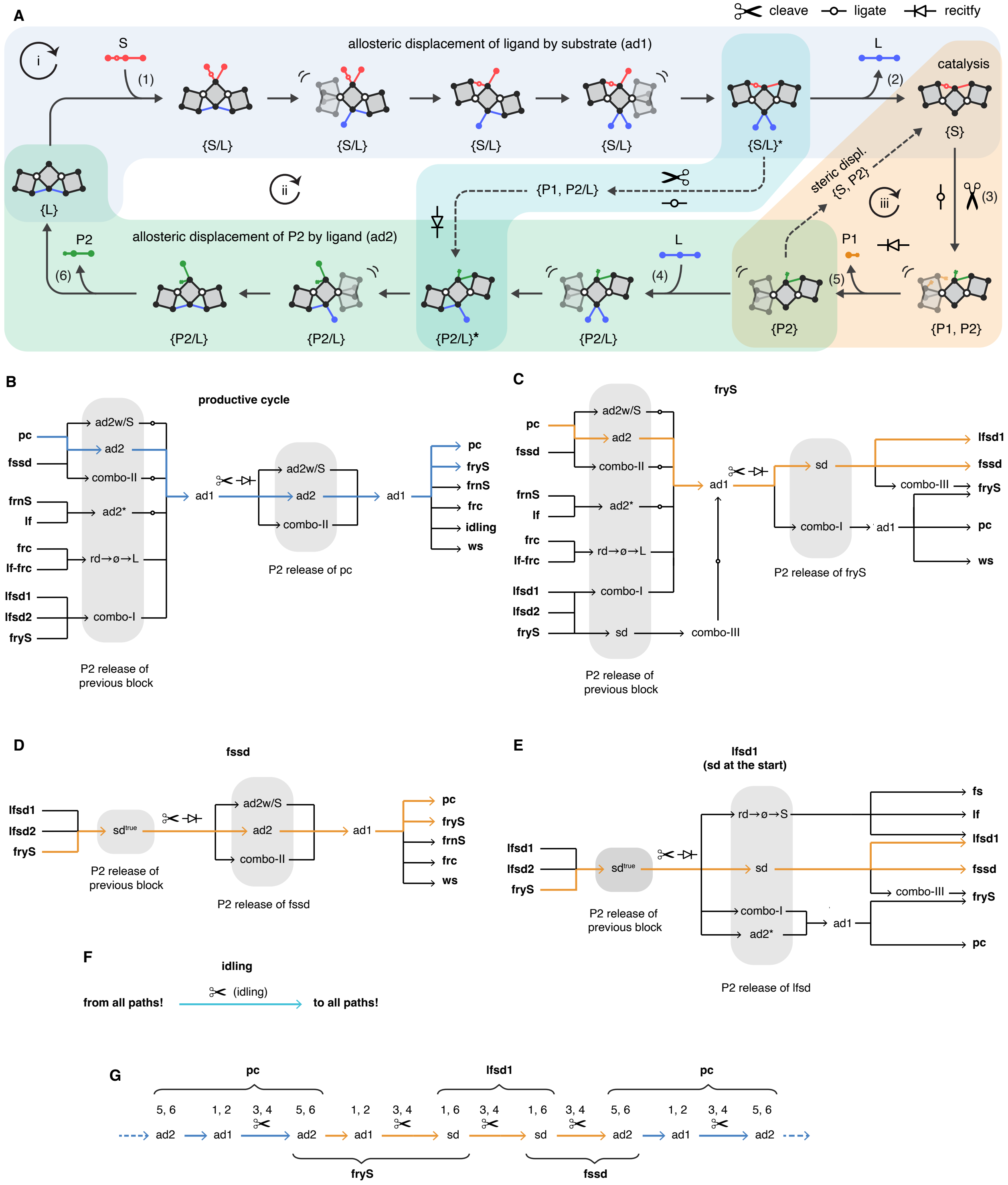

Fig. 8. Behavior blocks. A, B, C, D, E,

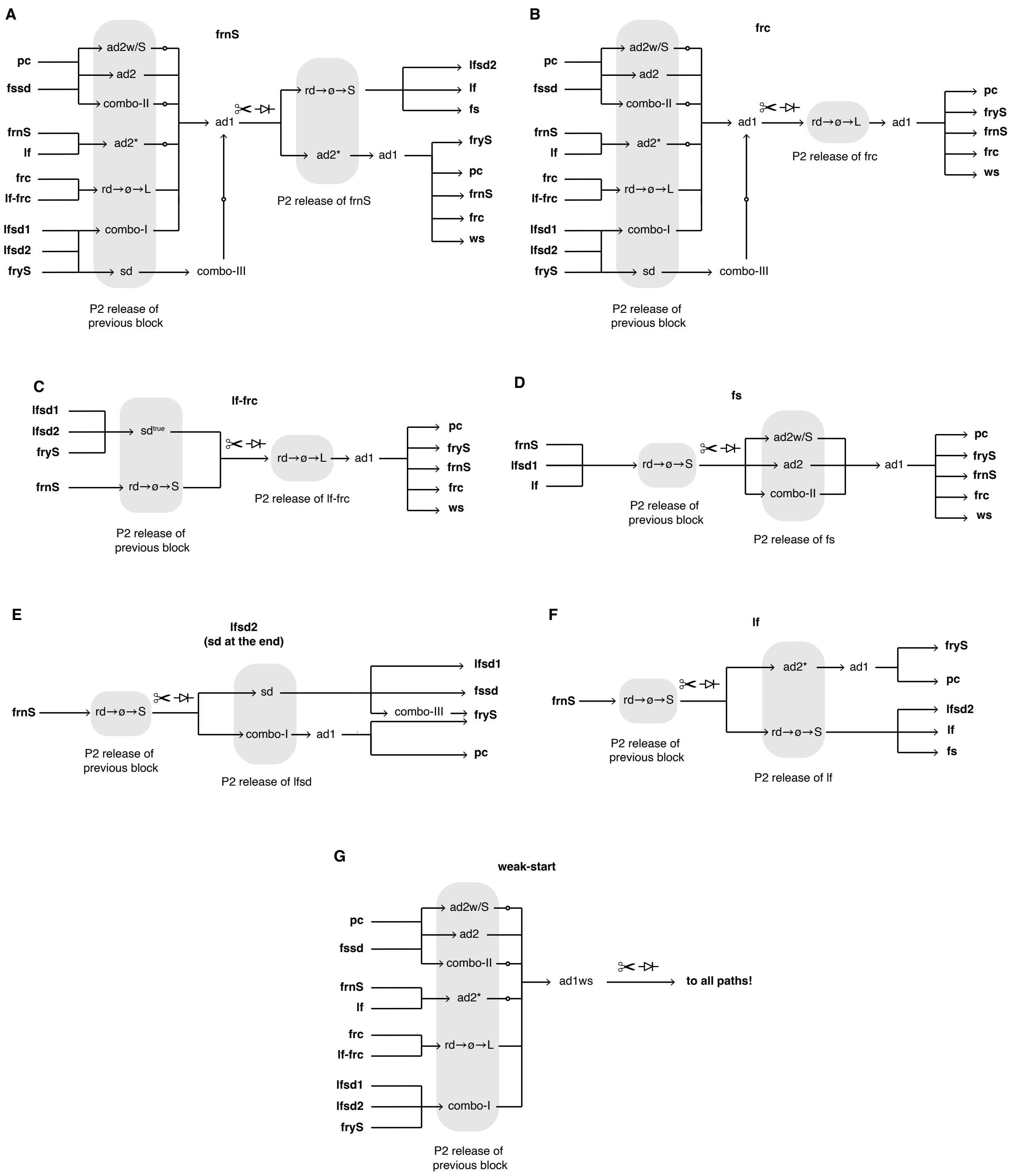

Fig. 9. Behavior blocks continued. A, B, C, D, E,

Identifying the behavior blocks

Step 1: The first step was to go through each trajectory, line by line, and record relevant binding information about substrate, ligand and p2.

For each substrate that bound, we recorded:

1. when it dissociated—if it dissociated as substrate
2. when cleavage happened, if it happened
3. when ligation happened, if it happened
4. when P1 and P2 (created by cleavage) dissociated

For each ligand that bound, we recorded:

1. when it bound
2. when it dissociated
3. if cleavage events took place, while it was bound
4. if P2 dissociated, while it was bound

For each P2 that dissociated, we recorded when it dissociated.

Step 2: In the second step, substrate turnover events were matched to specific behaviors that make them either part of a productive cylces, or part of a futile turnover. These behaviors are the initial definitions of the turnover blocks. The initial definitions are called "non-exclusive" (and labeled with nx superscript) because some turnover events satisfied conditions in more than one these "non-exclusive" blocks.

Initial & non-exclusive behavior blocks:

1. **fryS$^{\mathbf{nx}}$**: When P2 dissociates, substrate is bound, and ligand (if bound) is not fully bound. By default, ligand can be partially bound, though this is not explicitly tested for. The fryS^{nx} P2 release conditions satisfy reaction sequences sd, or ad2*, at the end of a fryS, and lfsd1.
2. **frnS$^{\mathbf{nx}}$**: When P2 dissociates, substrate is not bound at all, and ligand (if bound) is not fully bound. By default, ligand can be partially bound (in states {11} or {12}). In contrast to a fryS^{nx}, partially bound ligand is flagged here, for later use in defining other exclusive blocks. The frnS^{nx} P2 release conditions satisfy reaction sequences rd $\rightarrow \emptyset \rightarrow$ S and ad2* at the ends of frnS, lf and lfsd1. Combined with the P2 release conditions for frc^{nx}, they satisfy reaction sequence, rd $\rightarrow \emptyset \rightarrow$ L, at the end of a frc and lf-frc.
3. **frc$^{\mathbf{nx}}$**: When P2 dissociates, substrate is not bound at all, ligand is not fully bound, and following P2 dissociation, ligand binds to the empty enzyme. By default, ligand can be partially bound, though this is not explicitly tested for. Combined with frnS^{nx}, these P2 release conditions satisfy reaction sequence, rd $\rightarrow \emptyset \rightarrow$ L, at the end of a frc^{x}.
4. **fs$^{\mathbf{nx}}$**: When substrate binds and is subsequently cleaved, ligand is not bound at all. If it is not the first substrate binding event (turnover event) of the simulation, the fs^{nx} conditions satisfy reaction sequence, rd $\rightarrow \emptyset \rightarrow$ S, at the beginning of fs^{x}, lf^{x} and lfsd1^{x}, where rd in the reaction sequence corresponds to the P2 release event of the preceding block. If it is the first substrate binding event of the simulation, they satisfy reaction sequence, $\emptyset \rightarrow$ S, at their beginnings.
5. **fssd$^{\mathbf{nx}}$**: When substrate binds, P2 is bound, and when substrate is subsequently cleaved, ligand is not bound. These conditions eventually satisfy a sd at the beginning of a fssd, for the P2 release of the preceding block.
6. **ws$^{\mathbf{nx}}$**: When P2 dissociates, ligand is partially bound (ligand is any state but {10}). This satisfies the ad1ws condition.
7. **lf$^{\mathbf{nx}}$** (logical combination of frnS and fs): lf is defined as a combination of the conditions set for frnS and fs. Here the flag set in frnS is used to rule out partial binding of ligand during P2 dissociation. Thes conditions satisfy the beginning and ending of lf as defined in Fig.
8. **lfsd$^{\mathbf{nx}}$** (logical combination of fryS, frnS, fs, fssd): lfsd is defined so that a sd takes place at the beginning, end, or both the beginning and end of a substrate turnover event. Three logical combination of fryS, frnS, fs and fssd achieve this goal:
   (a) fssd, fs, and fryS are true; frnS is false (lfsd1, sd at the beginning and end)
   (b) fssd, fs and frnS are true; fryS is false (lfsd1, sd at the beginning only)
   (c) fs and fryS are true; frnS and fssd are false (lfsd2, sd at the end only)

9. **idling$^{\mathbf{nx}}$**: A futile event because ligand is bound during cleavage. Three cases are looked for, with each considering the possibility that substrate is cleaved (and thus ligated at least once) more that once before P1 dissociates, where category 2 and 3 are rare:
   (a) Ligand is bound at the first and last cleavage event
   (b) Ligand is bound at the first cleavage event, but dissociates before the last cleavage. This case only applies when there are more than one cleavage events
   (c) Ligand is NOT bound at the first cleavage event, but binds before the last cleavage. This case also only applies when there are more than one cleavage events.
10. **pc$^{\mathbf{nx}}$**: The productive cycle has two main conditions tested for (steps i and ii below) to establish that the substrate turnover event in question is a productive cycle. If these two conditions are met, steps iii and iv determine further details of the two P2 displacements that place, where the P2 displacement tested for in step iii, is not required to occur, given how productive cycles are defined (e.g. they can start with rd $\rightarrow$ ø $\rightarrow$ L).
   (i) When P2 dissociates, ligand is fully bound (in $\{10\}$). This P2 is P2_current, and this ligand is L2.
   (ii) The substrate from which P2_current was cleaved is matched to a viable ligand (L1), if possible. Viable ligand are ligands that dissociated when a substrate was fully bound (in $\{0\}$). If a match is made, the substrate turnover event is a productive cycle. Three conditions are met for a successful match:
      i. substrate binds to the enzyme when a viable ligand is already bound
      ii. substrate remains bound after the viable ligand dissociates
      iii. substrate is cleaved after the viable ligand dissociates
   (iii) If L1 displaced a P2 (P2_previous) when it bound, L1 is matched to this P2.
   (iv) P2_current is finally matched to L2.

Step 3: In the final step, a series of logical statements were used to construct mutually exclusive behavior blocks from the non-exclusive blocks, such that each exclusive block captured a unique set of pathways by which substrate was turned over, and all turnover events were accounted for.

A tricky aspect of the non-exclusive blocks is that some of them are defined in such a way that they capture unexpected, or unintended behavior, and this behavior is not always consistent with their naming. For example, fssd^{nx} captures steric displacements, but it also captures combo-I and combo-II displacements, because there is no restriction on the whether ligand is bound or not in the fssd^{nx} definition. These combos can show up at the start of a productive cycle, and thus the "futile-start" and "sd" monikers for fssd^{nx}, do match the "productive cycle" moniker, but the behavior of fssd^{nx} is consistent with the definition of a productive cycle.

Note that the starred exclusives (lfsd*, lf* and lf-frc*) are only defined as logical combinations of the non-exclusive blocks. However, in the code, lfsd^{nx} and lf^{nx} are already defined at step 2, before the final set of logical statements are used to make all the behavior blocks mutually exclusive from one another.

	pc^{nx}	pci^{nx}	$fryS^{nx}$	$frnS^{nx}$	frc^{nx}	$fssd^{nx}$	fs^{nx}	ws^{nx}	idl^{nx}
pc	1	0	0	0	0	0	0	0	0
	1	0	0	0	0	1	0	0	0
pci	0	1	0	0	0	0	0	0	0
	0	1	0	0	0	1	0	0	0
fryS	0	0	1	0	0	0	1	0	0
	0	0	1	0	0	1	1	0	0
frnS	0	0	0	1	0	0	0	0	0
	0	0	0	1	0	1	0	0	0
frc	0	0	0	1	1	0	0	0	0
lfsd*	0	0	1	0	0	1	1	0	0
	0	0	0	1	0	1	1	0	0
	0	0	1	0	0	0	1	0	0
fssd	0	0	0	0	0	1	1	0	0
lf*	0	0	0	1	0	0	1	0	0
lf-frc*	0	0	0	1	1	0	1	0	0
fs	0	0	0	0	0	0	1	0	0
ws	0	0	0	0	0	0	0	1	0
idl	0	0	0	0	0	0	0	0	1

Table 7: **Exclusive behavior blocks from non-exclusive blocks**. This table shows the sets of non-exclusive behavior blocks (top row, 'nx' subscript) that compose each exclusive behavior blocks (left column, no subscript). A '1' indicates that the non-exclusive block is an allowed element, and a '0' indicates that it is not an allowed element. The starred exclusive blocks, lfsd*, lf* and lf-frc*, do not have non-exclusive counterparts, as do the other blocks, and are only defined as compositions of the other non-exclusive blocks.

1.6 Movies

(All animations were made by Christian Swinehart (drafting@samizdat.co).)

Each movie is accompanied below by a dwell time plot (see examples in SI Figs. 10 and 11). In each dwell time plot, the time spent bound to the enzyme by each of the four reactants (dwell time) is represented by horizontal bars of color, with one color for each reactant: red for the substrate; orange for P1; green for P2; and blue for the ligand. The enzyme is represented the same as in the main text, except that all three squares are colored blue, and all the nodes are colored white when not bound. Instead, the reactants are depicted with colored nodes, although without any variation in shading. Thus, the substrate's nodes are all the same color red, P2's nodes are both the same green, and the ligand's nodes are the same blue. The final difference in representation is that the substrate is not depicted with a delta node, even though it is split in the same place.

List of movies:

Movie 1: single target cycle

Movie 2: seven consecutive target cycles

Movie 3: side by side clips of a simulation started with substrate and ligand (left), and a simulation started with substrate only (right).

Movie 4: steric displacement

Movie 5: idling

Movie 6: saturation

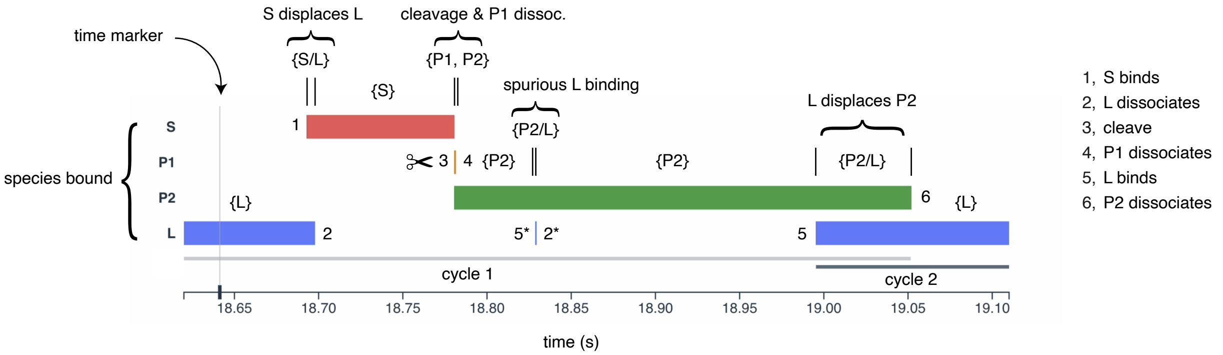

Fig. 10. Movie 1 dwell legend. This is a labeled version of the legend that appears in Movie 1. This dwell time plot shows a roughly a half second of the simulation, and the species that bound to the enzyme during this time. On the left, the row for each species is labeled ('species bound'), where S is shown in red, P1 in orange, P2 in green and L in blue. When multiple species are bound the bars of color overlap. For example, when S binds, close to 18.70 seconds, the red and blue bars overlap for a short section, during which the displacement of ligand by substrate takes place (see section labeled {S, L}). There are three more overlapping sections: {P1, P2}, where P1 and P2 are created just after cleavage; {P2, L}*, where short-lived spurious binding of L takes place; and {P2, L}, during which L displaces P2. From the beginning to roughly 19.05 seconds, one cycle (cycle 1) is completed. The beginning of cycle 1, where L first binds, takes place earlier in the simulation and is not shown. The end of cycle 1 overlaps with the beginning of cycle 2, where the overlap is the section where L displaces P2. The two cycles (cycle 1, grey; cycle 2, black) are demarcated at the bottom with horizontal lines.

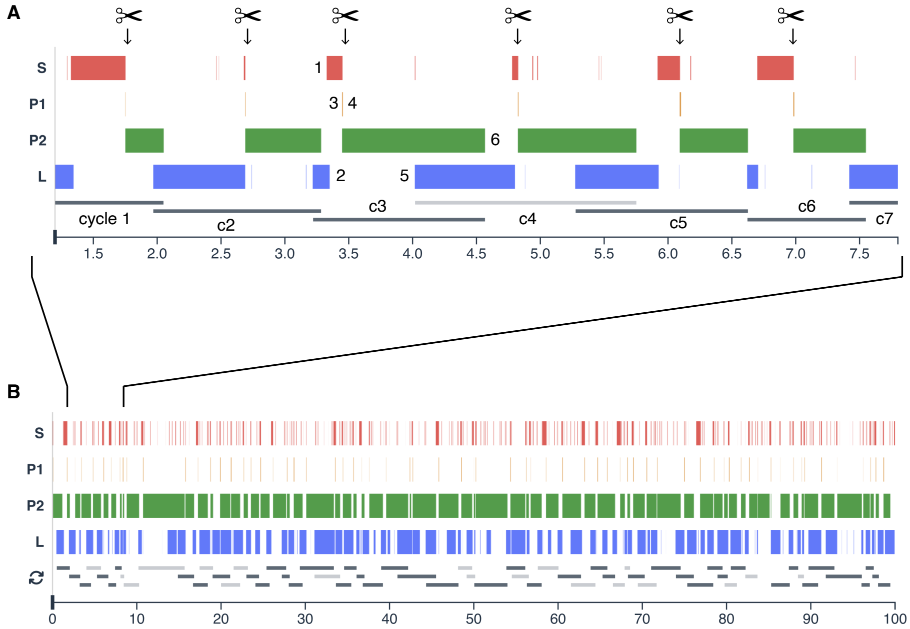

Fig. 11. Movie 2 dwell legend. A. This is the legend that appears in Movie 2. At the bottom, the seven cycles that take place (though cycles 1 and 7 are not complete) are demarcated. The six reactions are labeled by number for cycle 3. At the top the six cleavage events that take place are demarcated. **B.** This is the full 100 second simulation from which Movie 2 and Movie 1 were taken.

1.7 Pathway data

Tables 7 and 8 give the total counts for each pathway. Each total count is a sum of ten counts, where each count is from the ten simulations performed at each of the twenty-four substrate concentrations. Each simulation was run for 100 seconds. Thus, for each simulation (n) the turnover rate (v_n), with units of no. P2 released $\times$ s^{-1}, is the count for that simulation (c_n) divided by 100 seconds:

$$v_n = \frac{c_n}{100} \tag{18}$$

A mean turnover rate (v) was calculated for the ten simulations run:

$$v = \frac{1}{10}\sum_{n=1}^{10} v_n \tag{19}$$

and a standard deviation (sd) of the mean:

$$\text{sd} = \sqrt{\frac{1}{10}\sum_{n=1}^{10}(v_n - v)^2} \tag{20}$$

Each point on the plots shows the mean turnover rate for that pathway and its standard deviation.

[S] μM	total	idles	fryS	frnS	frc	fs	fssd	lf	lfsd	lf-frc	ws	pc	pci
1×10^{-4}	1	0	0	0	0	0	0	0	0	0	0	1	0
2×10^{-4}	4	0	0	0	0	0	0	0	0	0	0	4	0
5×10^{-4}	2	1	0	0	0	0	0	0	0	0	0	1	0
1×10^{-3}	19	1	0	1	0	0	0	0	0	0	0	17	0
2×10^{-3}	37	3	0	0	0	0	0	0	0	0	1	33	0
5×10^{-3}	93	1	0	0	0	2	0	0	0	0	0	90	0
1×10^{-2}	148	5	0	1	1	0	0	0	0	0	0	141	0
2×10^{-2}	238	8	0	1	0	3	0	0	0	0	0	225	1
5×10^{-2}	448	16	0	1	0	5	0	0	0	0	2	420	4
1×10^{-1}	624	11	1	2	0	7	0	0	0	0	0	597	6
2×10^{-1}	706	18	1	2	1	10	1	0	0	0	2	666	5
5×10^{-1}	819	24	13	0	2	8	9	0	0	0	0	760	3
1×10^{0}	875	25	33	4	0	13	28	0	1	0	1	765	5
2×10^{0}	924	32	51	0	0	8	47	0	4	0	1	775	6
5×10^{0}	984	30	104	3	0	11	92	0	13	0	1	724	6
1×10^{1}	1061	36	157	2	0	6	133	0	35	0	1	686	5
2×10^{1}	1193	41	225	0	0	7	194	0	77	0	2	639	8
5×10^{1}	1327	32	308	1	0	9	268	0	133	0	1	566	9
1×10^{2}	1476	41	331	0	0	5	303	0	203	0	6	587	0
2×10^{2}	1484	37	349	0	0	7	334	0	242	0	11	499	5
5×10^{2}	1236	35	283	0	0	7	254	0	196	0	12	444	5
1×10^{3}	768	12	183	0	0	6	171	0	122	0	17	256	1
2×10^{3}	358	8	86	0	0	7	79	0	49	0	11	118	0
5×10^{3}	103	2	12	0	0	6	19	0	23	0	10	31	0

Table 8: **Behavior block data for 'basic' simulations.**

k_{cat} s^{-1}	total	idles	fryS	frnS	frc	fs	fssd	lf	lfsd	lf-frc	ws	pc	pci
1	422	2	4	1	0	7	3	0	0	0	0	401	4
10	689	21	4	0	2	4	5	0	2	0	1	647	3
100	798	206	4	1	1	4	4	1	0	0	0	574	3
1×10^3	1051	803	2	1	0	7	3	0	0	0	0	235	0
1×10^4	1162	1118	0	0	0	9	0	0	0	0	0	35	0
1×10^5	1164	1151	0	0	0	7	1	0	0	0	1	4	0
1×10^6	1181	1170	0	0	0	7	4	0	0	0	0	0	0
1×10^7	1124	1114	0	0	0	8	2	0	0	0	0	0	0
1×10^8	1097	1091	0	1	0	5	1	0	0	0	0	0	0
1×10^9	1109	1102	0	0	0	5	1	0	0	0	1	0	0

Table 9: **kcat variation simulations.**

k_{lig} s^{-1}	total	idles	fryS	frnS	frc	fs	fssd	lf	lfsd	lf-frc	ws	pc	pci
1	700	22	4	3	0	5	4	0	0	0	1	655	6
10	689	21	4	0	2	4	5	0	2	0	1	647	3
100	706	27	7	2	2	9	4	0	0	0	0	649	6
1×10^3	548	24	2	2	0	5	3	0	1	0	0	499	12
1×10^4	227	10	2	0	0	4	0	0	0	0	0	180	31
1×10^5	70	5	0	0	0	0	0	0	0	0	0	22	43
1×10^6	18	4	0	0	0	0	0	0	0	0	0	1	13
1×10^7	3	1	0	0	0	0	0	0	0	0	0	0	2
1×10^8	0	0	0	0	0	0	0	0	0	0	0	0	0
1×10^9	0	0	0	0	0	0	0	0	0	0	0	0	0

Table 10: **klig variation simulations.**

1.8 Saturation

Saturation (SI Fig. 12) takes place at very high concentrations of substrate (e.g. $> 200\ \mu$M), where the rate at which substrate binds the enzyme surpasses the rate at which bound ligand dissociates from its nodes. At peak saturation, the substrate binding site is occupied by three substrate molecules, and the ligand binding site by one trivalently bound ligand ({S, S, S, L})(SI Fig. 12A). The bound substrates cannot displace the ligand because the ligand controls the geometry, and each substrate sterically blocks the other from binding divalently or trivalently. The saturation state persists because if one substrate molecule dissociates, it will likely be replaced by another before the ligand dissociates from one of its nodes and allows a bound substrate to bind divalently.

The peak saturation state forms during the displacement of P2 by ligand (SI Fig. 12B). During the displacement process, unoccupied nodes at the substrate binding site are rapidly bound by substrate. The first node to be occupied by substrate is a, which becomes available after P1 dissociates. The most likely second node to be occupied by substrate is c, which is a three-step process involving ligand and the displacement of P2 by ligand.

To describe this process, the first thing to note is that spurious dissociates of P2 from c are much more likely than b, because the node c reaction is much weaker than that of node b ($k_{\text{off-c}} = 680\,\text{s}^{-1}$ vs. $k_{\text{off-b}} = 3\,\text{s}^{-1}$). During spurious dissociations of P2 from node c, ligand can bind trivalently by capturing node f and the right degree of freedom, thereby inhibiting P2 from rebinding c (SI Fig. 12B, 4th state from top). In this state, where P2 is still bound at node b and ligand is bound trivalently, substrate will likely bind at node c before P2 dissociates from node b and is released into solution, as the rate at which substrate binds at high [S] is much greater than the rate at which P2 dissociates from b ($k_{\text{on-S}} = 4.5 \times 10^4\,\text{s}^{-1}$ at [S] = 5 mM[1] vs. $k_{\text{off-b}} = 3\,\text{s}^{-1}$.). Upon substrate binding to node c, the precursor state of saturation is reached, in which one P2, two substrates, and one ligand are bound to the enzyme ({P2, S, S, L})(SI Fig. 12B, 5th state from top). When P2 dissociates from node b, rapid substrate binding to node b will complete formation of the peak saturation complex, in which three substrates and one ligand molecule are bound ({S, S, S, L}). Peak saturation will dynamically persist, as described above, until one substrate molecule manages to displace ligand.

[1] $k_{\text{on-S}}$, at 5 mM [S], is calculated by multiplying the bimolecular rate constant, $k_{\text{bi}} = 9 \times 10^6\,\text{M}^{-1}\text{s}^{-1}$, by 5 mM

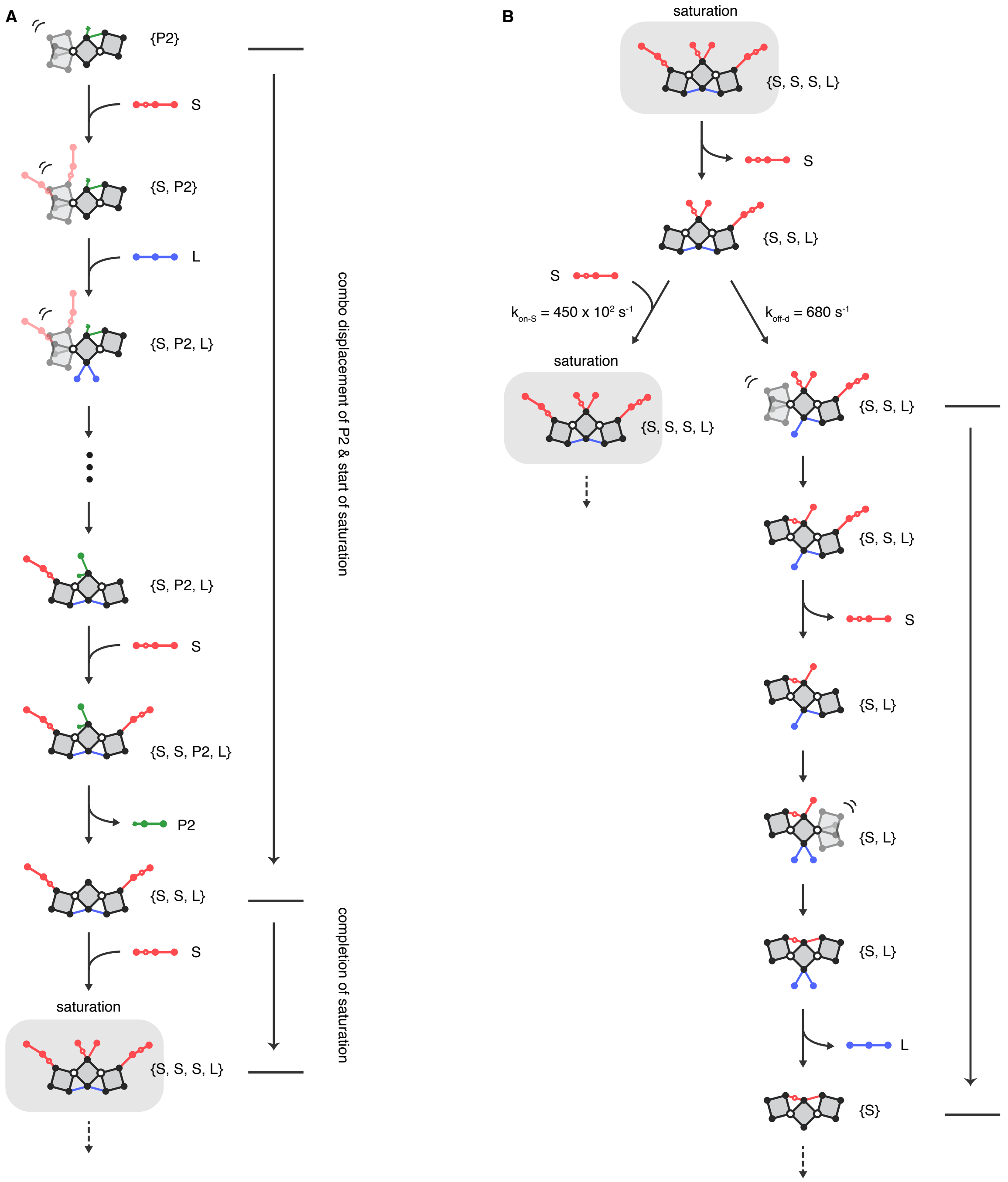

Fig. 12. Saturation. A, Pathway to saturation. Beginning with the P2-bound state ({P2}), substrate, at high concentration, binds at node a, followed by ligand. Trivalent ligand binding (abbreviated by '...') displaces P2, which exposes nodes a and b for substrate binding, and finally, substrate saturation. **B,** Persistence of the saturation state. The saturation state persists because any substrate that dissociates is likely replaced (left branch) before ligand dissociates from one of its nodes and opens up a pathway for the displacement of ligand by substrate (right branch). Substrate replacement beats ligand dissociation because at high [S], the binding rate surpasses the ligand dissociation by orders of magnitude. For example, at 5 mM [S], $k_{\text{on-S}} = 450 \times 10^2\,\text{s}^{-1}$, whereas $k_{\text{off-d}} = 680\,\text{s}^{-1}$. The figure shows the saturation state decaying by dissociation of substrate at node a (the second state from top; state {4, 5, 10} in basis form, see SI Fig. 2), but it can also decay by dissociation of substrate from node c (state {3, 5, 10} in basis form).

1.9 States of the system

{18}, {0}, {1}, {2}, {3}, {4}, {5}, {6}, {7}, {8}, {9}, {10}, {11}, {12}, {13}, {14}, {15}, {0, 13}, {0, 13, 14}, {0, 13, 14, 15}, {0, 13, 15}, {0, 14}, {0, 14, 15}, {0, 15}, {1, 4}, {1, 4, 11}, {1, 4, 11, 14}, {1, 4, 13}, {1, 4, 13, 14}, {1, 4, 13, 14, 15}, {1, 4, 13, 15}, {1, 4, 14}, {1, 4, 14, 15}, {1, 4, 15}, {1, 7}, {1, 7, 11}, {1, 7, 11, 14}, {1, 7, 13}, {1, 7, 13, 14}, {1, 7, 13, 14, 15}, {1, 7, 13, 15}, {1, 7, 14}, {1, 7, 14, 15}, {1, 7, 15}, {1, 11}, {1, 11, 14}, {1, 13}, {1, 13, 14}, {1, 13, 14, 15}, {1, 13, 15}, {1, 14}, {1, 14, 15}, {1, 15}, {2, 3}, {2, 3, 12}, {2, 3, 12, 13}, {2, 3, 13}, {2, 3, 13, 14}, {2, 3, 13, 14, 15}, {2, 3, 13, 15}, {2, 3, 14}, {2, 3, 14, 15}, {2, 3, 15}, {2, 9}, {2, 9, 12}, {2, 9, 12, 13}, {2, 9, 13}, {2, 9, 13, 14}, {2, 9, 13, 14, 15}, {2, 9, 13, 15}, {2, 9, 14}, {2, 9, 14, 15}, {2, 9, 15}, {2, 12}, {2, 12, 13}, {2, 13}, {2, 13, 14}, {2, 13, 14, 15}, {2, 13, 15}, {2, 14}, {2, 14, 15}, {2, 15}, {3, 4}, {3, 4, 5}, {3, 4, 5, 10}, {3, 4, 5, 11}, {3, 4, 5, 11, 14}, {3, 4, 5, 12}, {3, 4, 5, 12, 13}, {3, 4, 5, 13}, {3, 4, 5, 13, 14}, {3, 4, 5, 13, 14, 15}, {3, 4, 5, 13, 15}, {3, 4, 5, 14}, {3, 4, 5, 14, 15}, {3, 4, 5, 15}, {3, 4, 8}, {3, 4, 8, 10}, {3, 4, 8, 11}, {3, 4, 8, 11, 14}, {3, 4, 8, 12}, {3, 4, 8, 12, 13}, {3, 4, 8, 13}, {3, 4, 8, 13, 14}, {3, 4, 8, 13, 14, 15}, {3, 4, 8, 13, 15}, {3, 4, 8, 14}, {3, 4, 8, 14, 15}, {3, 4, 8, 15}, {3, 4, 10}, {3, 4, 11}, {3, 4, 11, 14}, {3, 4, 12}, {3, 4, 12, 13}, {3, 4, 13}, {3, 4, 13, 14}, {3, 4, 13, 14, 15}, {3, 4, 13, 15}, {3, 4, 14}, {3, 4, 14, 15}, {3, 4, 15}, {3, 5}, {3, 5, 7}, {3, 5, 7, 10}, {3, 5, 7, 11}, {3, 5, 7, 11, 14}, {3, 5, 7, 12}, {3, 5, 7, 12, 13}, {3, 5, 7, 13}, {3, 5, 7, 13, 14}, {3, 5, 7, 13, 14, 15}, {3, 5, 7, 13, 15}, {3, 5, 7, 14}, {3, 5, 7, 14, 15}, {3, 5, 7, 15}, {3, 5, 10}, {3, 5, 11}, {3, 5, 11, 14}, {3, 5, 12}, {3, 5, 12, 13}, {3, 5, 13}, {3, 5, 13, 14}, {3, 5, 13, 14, 15}, {3, 5, 13, 15}, {3, 5, 14}, {3, 5, 14, 15}, {3, 5, 15}, {3, 6}, {3, 6, 12}, {3, 6, 12, 13}, {3, 6, 13}, {3, 6, 13, 14}, {3, 6, 13, 14, 15}, {3, 6, 13, 15}, {3, 6, 14}, {3, 6, 14, 15}, {3, 6, 15}, {3, 7}, {3, 7, 8}, {3, 7, 8, 10}, {3, 7, 8, 11}, {3, 7, 8, 11, 14}, {3, 7, 8, 12}, {3, 7, 8, 12, 13}, {3, 7, 8, 13}, {3, 7, 8, 13, 14}, {3, 7, 8, 13, 14, 15}, {3, 7, 8, 13, 15}, {3, 7, 8, 14}, {3, 7, 8, 14, 15}, {3, 7, 8, 15}, {3, 7, 10}, {3, 7, 11}, {3, 7, 11, 14}, {3, 7, 12}, {3, 7, 12, 13}, {3, 7, 13}, {3, 7, 13, 14}, {3, 7, 13, 14, 15}, {3, 7, 13, 15}, {3, 7, 14}, {3, 7, 14, 15}, {3, 7, 15}, {3, 8}, {3, 8, 10}, {3, 8, 11}, {3, 8, 11, 14}, {3, 8, 12}, {3, 8, 12, 13}, {3, 8, 13}, {3, 8, 13, 14}, {3, 8, 13, 14, 15}, {3, 8, 13, 15}, {3, 8, 14}, {3, 8, 14, 15}, {3, 8, 15}, {3, 10}, {3, 11}, {3, 11, 14}, {3, 12}, {3, 12, 13}, {3, 13}, {3, 13, 14}, {3, 13, 14, 15}, {3, 13, 15}, {3, 14}, {3, 14, 15}, {3, 15}, {4, 5}, {4, 5, 9}, {4, 5, 9, 10}, {4, 5, 9, 11}, {4, 5, 9, 11, 14}, {4, 5, 9, 12}, {4, 5, 9, 12, 13}, {4, 5, 9, 13}, {4, 5, 9, 13, 14}, {4, 5, 9, 13, 14, 15}, {4, 5, 9, 13, 15}, {4, 5, 9, 14}, {4, 5, 9, 14, 15}, {4, 5, 9, 15}, {4, 5, 10}, {4, 5, 11}, {4, 5, 11, 14}, {4, 5, 12}, {4, 5, 12, 13}, {4, 5, 13}, {4, 5, 13, 14}, {4, 5, 13, 14, 15}, {4, 5, 13, 15}, {4, 5, 14}, {4, 5, 14, 15}, {4, 5, 15}, {4, 8}, {4, 8, 9}, {4, 8, 9, 10}, {4, 8, 9, 11}, {4, 8, 9, 11, 14}, {4, 8, 9, 12}, {4, 8, 9, 12, 13}, {4, 8, 9, 13}, {4, 8, 9, 13, 14}, {4, 8, 9, 13, 14, 15}, {4, 8, 9, 13, 15}, {4, 8, 9, 14}, {4, 8, 9, 14, 15}, {4, 8, 9, 15}, {4, 8, 10}, {4, 8, 11}, {4, 8, 11, 14}, {4, 8, 12}, {4, 8, 12, 13}, {4, 8, 13}, {4, 8, 13, 14}, {4, 8, 13, 14, 15}, {4, 8, 13, 15}, {4, 8, 14}, {4, 8, 14, 15}, {4, 8, 15}, {4, 9}, {4, 9, 10}, {4, 9, 11}, {4, 9, 11, 14}, {4, 9, 12}, {4, 9, 12, 13}, {4, 9, 13}, {4, 9, 13, 14}, {4, 9, 13, 14, 15}, {4, 9, 13, 15}, {4, 9, 14}, {4, 9, 14, 15}, {4, 9, 15}, {4, 10}, {4, 11}, {4, 11, 14}, {4, 12}, {4, 12, 13}, {4, 13}, {4, 13, 14}, {4, 13, 14, 15}, {4, 13, 15}, {4, 14}, {4, 14, 15}, {4, 15}, {5, 7}, {5, 7, 9}, {5, 7, 9, 10}, {5, 7, 9, 11}, {5, 7, 9, 11, 14}, {5, 7, 9, 12}, {5, 7, 9, 12, 13}, {5, 7, 9, 13}, {5, 7, 9, 13, 14}, {5, 7, 9, 13, 14, 15}, {5, 7, 9, 13, 15}, {5, 7, 9, 14}, {5, 7, 9, 14, 15}, {5, 7, 9, 15}, {5, 7, 10}, {5, 7, 11}, {5, 7, 11, 14}, {5, 7, 12}, {5, 7, 12, 13}, {5, 7, 13}, {5, 7, 13, 14}, {5, 7, 13, 14, 15}, {5, 7, 13, 15}, {5, 7, 14}, {5, 7, 14, 15}, {5, 7, 15}, {5, 9}, {5, 9, 10}, {5, 9, 11}, {5, 9, 11, 14}, {5, 9, 12}, {5, 9, 12, 13}, {5, 9, 13}, {5, 9, 13, 14}, {5, 9, 13, 14, 15}, {5, 9, 13, 15}, {5, 9, 14}, {5, 9, 14, 15}, {5, 9, 15}, {5, 10}, {5, 11}, {5, 11, 14}, {5, 12}, {5, 12, 13}, {5, 13}, {5, 13, 14}, {5, 13, 14, 15}, {5, 13, 15}, {5, 14}, {5, 14, 15}, {5, 15}, {6, 9}, {6, 9, 12}, {6, 9, 12, 13}, {6, 9, 13}, {6, 9, 13, 14}, {6, 9, 13, 14, 15}, {6, 9, 13, 15}, {6, 9, 14}, {6, 9, 14, 15}, {6, 9, 15}, {6, 12}, {6, 12, 13}, {6, 13}, {6, 13, 14}, {6, 13, 14, 15}, {6, 13, 15}, {6, 14}, {6, 14, 15}, {6, 15}, {7, 8}, {7, 8, 9}, {7, 8, 9, 10}, {7, 8, 9, 11}, {7, 8, 9, 11, 14}, {7, 8, 9, 12}, {7, 8, 9, 12, 13}, {7, 8, 9, 13}, {7, 8, 9, 13, 14}, {7, 8, 9, 13, 14, 15}, {7, 8, 9, 13, 15}, {7, 8, 9, 14}, {7, 8, 9, 14, 15}, {7, 8, 9, 15}, {7, 8, 10}, {7, 8, 11}, {7, 8, 11, 14}, {7, 8, 12}, {7, 8, 12, 13}, {7, 8, 13}, {7, 8, 13, 14}, {7, 8, 13, 14, 15}, {7, 8, 13, 15}, {7, 8, 14}, {7, 8, 14, 15}, {7, 8, 15}, {7, 9}, {7, 9, 10}, {7, 9, 11}, {7, 9, 11, 14}, {7, 9, 12}, {7, 9, 12, 13}, {7, 9, 13}, {7, 9, 13, 14}, {7, 9, 13, 14, 15}, {7, 9, 13, 15}, {7, 9, 14}, {7, 9, 14, 15}, {7, 9, 15}, {7, 10}, {7, 11}, {7, 11, 14}, {7, 12}, {7, 12, 13}, {7, 13}, {7, 13, 14}, {7, 13, 14, 15}, {7, 13, 15}, {7, 14}, {7, 14, 15}, {7, 15}, {8, 9}, {8, 9, 10}, {8, 9, 11}, {8, 9, 11, 14}, {8, 9, 12}, {8, 9, 12, 13}, {8, 9, 13}, {8, 9, 13, 14}, {8, 9, 13, 14, 15}, {8, 9, 13, 15}, {8, 9, 14}, {8, 9, 14, 15}, {8, 9, 15}, {8, 10}, {8, 11}, {8, 11, 14}, {8, 12}, {8, 12, 13}, {8, 13}, {8, 13, 14}, {8, 13, 14, 15}, {8, 13, 15}, {8, 14}, {8, 14, 15}, {8, 15}, {9, 10}, {9, 11}, {9, 11, 14}, {9, 12}, {9, 12, 13}, {9, 13}, {9, 13, 14}, {9, 13, 14, 15}, {9, 13, 15}, {9, 14}, {9, 14, 15}, {9, 15}, {11, 14}, {12, 13}, {13, 14}, {13, 14, 15}, {13, 15}, {14, 15}

1.10 Possible transitions out of each state

{18} → {3}, {4}, {5}, {7}, {8}, {9}, {13}, {14}, {15}
{0} → {1}, {2}, {0, 13}, {0, 14}, {0, 15}, {6, 9}
{1} → {0}, {3}, {5}, {1, 4}, {1, 7}, {1, 13}, {1, 14}, {1, 15}
{2} → {0}, {4}, {5}, {2, 3}, {2, 9}, {2, 13}, {2, 14}, {2, 15}
{3} → {1}, {18}, {3, 4}, {3, 5}, {3, 7}, {3, 8}, {3, 13}, {3, 14}, {3, 15}
{4} → {2}, {18}, {3, 4}, {4, 5}, {4, 8}, {4, 9}, {4, 13}, {4, 14}, {4, 15}
{5} → {1}, {2}, {18}, {3, 5}, {4, 5}, {5, 7}, {5, 9}, {5, 13}, {5, 14}, {5, 15}
{6} → {7}, {8}, {3, 6}, {6, 9}, {6, 13}, {6, 14}, {6, 15}
{7} → {6}, {18}, {3, 7}, {5, 7}, {7, 8}, {7, 9}, {7, 13}, {7, 14}, {7, 15}
{8} → {6}, {18}, {3, 8}, {4, 8}, {7, 8}, {8, 9}, {8, 13}, {8, 14}, {8, 15}
{9} → {18}, {4, 9}, {5, 9}, {7, 9}, {8, 9}, {9, 13}, {9, 14}, {9, 15}
{10} → {11}, {12}, {3, 10}, {4, 10}, {5, 10}, {7, 10}, {8, 10}, {9, 10}
{11} → {10}, {13}, {15}, {3, 11}, {4, 11}, {5, 11}, {7, 11}, {8, 11}, {9, 11}, {11, 14}
{12} → {10}, {14}, {15}, {3, 12}, {4, 12}, {5, 12}, {7, 12}, {8, 12}, {9, 12}, {12, 13}
{13} → {11}, {18}, {3, 13}, {4, 13}, {5, 13}, {7, 13}, {8, 13}, {9, 13}, {13, 14}, {13, 15}
{14} → {12}, {18}, {3, 14}, {4, 14}, {5, 14}, {7, 14}, {8, 14}, {9, 14}, {13, 14}, {14, 15}
{15} → {11}, {12}, {18}, {3, 15}, {4, 15}, {5, 15}, {7, 15}, {8, 15}, {9, 15}, {13, 15}, {14, 15}
{0, 13} → {1, 13}, {2, 13}, {0}, {0, 13, 14}, {0, 13, 15}, {6, 9, 13}
{0, 13, 14} → {1, 13, 14}, {2, 13, 14}, {0, 14}, {0, 13}, {0, 13, 14, 15}, {6, 9, 13, 14}
{0, 13, 14, 15} → {1, 13, 14, 15}, {2, 13, 14, 15}, {0, 14, 15}, {0, 13, 15}, {0, 13, 14}, {6, 9, 13, 14, 15}
{0, 13, 15} → {1, 13, 15}, {2, 13, 15}, {0, 15}, {0, 13}, {0, 13, 14, 15}, {6, 9, 13, 15}
{0, 14} → {1, 14}, {2, 14}, {0}, {0, 13, 14}, {0, 14, 15}, {6, 9, 14}
{0, 14, 15} → {1, 14, 15}, {2, 14, 15}, {0, 15}, {0, 14}, {0, 13, 14, 15}, {6, 9, 14, 15}
{0, 15} → {1, 15}, {2, 15}, {0}, {0, 13, 15}, {0, 14, 15}, {6, 9, 15}
{1, 4} → {3, 4}, {4, 5}, {1}, {1, 4, 13}, {1, 4, 14}, {1, 4, 15}
{1, 4, 11} → {3, 4, 11}, {4, 5, 11}, {1, 11}, {1, 4, 13}, {1, 4, 15}, {1, 4, 11, 14}
{1, 4, 11, 14} → {3, 4, 11, 14}, {4, 5, 11, 14}, {1, 11, 14}, {1, 4, 13, 14}, {1, 4, 14, 15}, {1, 4, 11}
{1, 4, 13} → {3, 4, 13}, {4, 5, 13}, {1, 13}, {1, 4, 11}, {1, 4}, {1, 4, 13, 14}, {1, 4, 13, 15}
{1, 4, 13, 14} → {3, 4, 13, 14}, {4, 5, 13, 14}, {1, 13, 14}, {1, 4, 11, 14}, {1, 4, 14}, {1, 4, 13}, {1, 4, 13, 14, 15}
{1, 4, 13, 14, 15} → {3, 4, 13, 14, 15}, {4, 5, 13, 14, 15}, {1, 13, 14, 15}, {1, 4, 14, 15}, {1, 4, 13, 15}, {1, 4, 13, 14}
{1, 4, 13, 15} → {3, 4, 13, 15}, {4, 5, 13, 15}, {1, 13, 15}, {1, 4, 15}, {1, 4, 13}, {1, 4, 13, 14, 15}
{1, 4, 14} → {3, 4, 14}, {4, 5, 14}, {1, 14}, {1, 4}, {1, 4, 13, 14}, {1, 4, 14, 15}
{1, 4, 14, 15} → {3, 4, 14, 15}, {4, 5, 14, 15}, {1, 14, 15}, {1, 4, 15}, {1, 4, 11, 14}, {1, 4, 14}, {1, 4, 13, 14, 15}

{1, 4, 15} → {3, 4, 15}, {4, 5, 15}, {1, 15}, {1, 4, 11}, {1, 4}, {1, 4, 13, 15}, {1, 4, 14, 15}
{1, 7} → {3, 7}, {5, 7}, {1}, {1, 7, 13}, {1, 7, 14}, {1, 7, 15}
{1, 7, 11} → {3, 7, 11}, {5, 7, 11}, {1, 11}, {1, 7, 13}, {1, 7, 15}, {1, 7, 11, 14}
{1, 7, 11, 14} → {3, 7, 11, 14}, {5, 7, 11, 14}, {1, 11, 14}, {1, 7, 13, 14}, {1, 7, 14, 15}, {1, 7, 11}
{1, 7, 13} → {3, 7, 13}, {5, 7, 13}, {1, 13}, {1, 7, 11}, {1, 7}, {1, 7, 13, 14}, {1, 7, 13, 15}
{1, 7, 13, 14} → {3, 7, 13, 14}, {5, 7, 13, 14}, {1, 13, 14}, {1, 7, 11, 14}, {1, 7, 14}, {1, 7, 13}, {1, 7, 13, 14, 15}
{1, 7, 13, 14, 15} → {3, 7, 13, 14, 15}, {5, 7, 13, 14, 15}, {1, 13, 14, 15}, {1, 7, 14, 15}, {1, 7, 13, 15}, {1, 7, 13, 14}
{1, 7, 13, 15} → {3, 7, 13, 15}, {5, 7, 13, 15}, {1, 13, 15}, {1, 7, 15}, {1, 7, 13}, {1, 7, 13, 14, 15}
{1, 7, 14} → {3, 7, 14}, {5, 7, 14}, {1, 14}, {1, 7}, {1, 7, 13, 14}, {1, 7, 14, 15}
{1, 7, 14, 15} → {3, 7, 14, 15}, {5, 7, 14, 15}, {1, 14, 15}, {1, 7, 15}, {1, 7, 11, 14}, {1, 7, 14}, {1, 7, 13, 14, 15}
{1, 7, 15} → {3, 7, 15}, {5, 7, 15}, {1, 15}, {1, 7, 11}, {1, 7}, {1, 7, 13, 15}, {1, 7, 14, 15}
{1, 11} → {3, 11}, {5, 11}, {1, 13}, {1, 15}, {1, 4, 11}, {1, 7, 11}, {1, 11, 14}
{1, 11, 14} → {3, 11, 14}, {5, 11, 14}, {1, 13, 14}, {1, 14, 15}, {1, 11}, {1, 4, 11, 14}, {1, 7, 11, 14}
{1, 13} → {0, 13}, {3, 13}, {5, 13}, {1, 11}, {1}, {1, 4, 13}, {1, 7, 13}, {1, 13, 14}, {1, 13, 15}
{1, 13, 14} → {0, 13, 14}, {3, 13, 14}, {5, 13, 14}, {1, 11, 14}, {1, 14}, {1, 13}, {1, 4, 13, 14}, {1, 7, 13, 14}, {1, 13, 14, 15}
{1, 13, 14, 15} → {0, 13, 14, 15}, {3, 13, 14, 15}, {5, 13, 14, 15}, {1, 14, 15}, {1, 13, 15}, {1, 13, 14}, {1, 4, 13, 14, 15}, {1, 7, 13, 14, 15}
{1, 13, 15} → {0, 13, 15}, {3, 13, 15}, {5, 13, 15}, {1, 15}, {1, 13}, {1, 4, 13, 15}, {1, 7, 13, 15}, {1, 13, 14, 15}
{1, 14} → {0, 14}, {3, 14}, {5, 14}, {1}, {1, 4, 14}, {1, 7, 14}, {1, 13, 14}, {1, 14, 15}
{1, 14, 15} → {0, 14, 15}, {3, 14, 15}, {5, 14, 15}, {1, 15}, {1, 11, 14}, {1, 14}, {1, 4, 14, 15}, {1, 7, 14, 15}, {1, 13, 14, 15}
{1, 15} → {0, 15}, {3, 15}, {5, 15}, {1, 11}, {1}, {1, 4, 15}, {1, 7, 15}, {1, 13, 15}, {1, 14, 15}
{2, 3} → {3, 4}, {3, 5}, {2}, {2, 3, 13}, {2, 3, 14}, {2, 3, 15}
{2, 3, 12} → {3, 4, 12}, {3, 5, 12}, {2, 12}, {2, 3, 14}, {2, 3, 15}, {2, 3, 12, 13}
{2, 3, 12, 13} → {3, 4, 12, 13}, {3, 5, 12, 13}, {2, 12, 13}, {2, 3, 13, 14}, {2, 3, 13, 15}, {2, 3, 12}
{2, 3, 13} → {3, 4, 13}, {3, 5, 13}, {2, 13}, {2, 3}, {2, 3, 13, 14}, {2, 3, 13, 15}
{2, 3, 13, 14} → {3, 4, 13, 14}, {3, 5, 13, 14}, {2, 13, 14}, {2, 3, 14}, {2, 3, 12, 13}, {2, 3, 13}, {2, 3, 13, 14, 15}
{2, 3, 13, 14, 15} → {3, 4, 13, 14, 15}, {3, 5, 13, 14, 15}, {2, 13, 14, 15}, {2, 3, 14, 15}, {2, 3, 13, 15}, {2, 3, 13, 14}
{2, 3, 13, 15} → {3, 4, 13, 15}, {3, 5, 13, 15}, {2, 13, 15}, {2, 3, 15}, {2, 3, 12, 13}, {2, 3, 13}, {2, 3, 13, 14, 15}
{2, 3, 14} → {3, 4, 14}, {3, 5, 14}, {2, 14}, {2, 3, 12}, {2, 3}, {2, 3, 13, 14}, {2, 3, 14, 15}
{2, 3, 14, 15} → {3, 4, 14, 15}, {3, 5, 14, 15}, {2, 14, 15}, {2, 3, 15}, {2, 3, 14}, {2, 3, 13, 14, 15}
{2, 3, 15} → {3, 4, 15}, {3, 5, 15}, {2, 15}, {2, 3, 12}, {2, 3}, {2, 3, 13, 15}, {2, 3, 14, 15}
{2, 9} → {4, 9}, {5, 9}, {2}, {2, 9, 13}, {2, 9, 14}, {2, 9, 15}
{2, 9, 12} → {4, 9, 12}, {5, 9, 12}, {2, 12}, {2, 9, 14}, {2, 9, 15}, {2, 9, 12, 13}
{2, 9, 12, 13} → {4, 9, 12, 13}, {5, 9, 12, 13}, {2, 12, 13}, {2, 9, 13, 14}, {2, 9, 13, 15}, {2, 9, 12}
{2, 9, 13} → {4, 9, 13}, {5, 9, 13}, {2, 13}, {2, 9}, {2, 9, 13, 14}, {2, 9, 13, 15}
{2, 9, 13, 14} → {4, 9, 13, 14}, {5, 9, 13, 14}, {2, 13, 14}, {2, 9, 14}, {2, 9, 12, 13}, {2, 9, 13}, {2, 9, 13, 14, 15}
{2, 9, 13, 14, 15} → {4, 9, 13, 14, 15}, {5, 9, 13, 14, 15}, {2, 13, 14, 15}, {2, 9, 14, 15}, {2, 9, 13, 15}, {2, 9, 13, 14}
{2, 9, 13, 15} → {4, 9, 13, 15}, {5, 9, 13, 15}, {2, 13, 15}, {2, 9, 15}, {2, 9, 12, 13}, {2, 9, 13}, {2, 9, 13, 14, 15}
{2, 9, 14} → {4, 9, 14}, {5, 9, 14}, {2, 14}, {2, 9, 12}, {2, 9}, {2, 9, 13, 14}, {2, 9, 14, 15}
{2, 9, 14, 15} → {4, 9, 14, 15}, {5, 9, 14, 15}, {2, 14, 15}, {2, 9, 15}, {2, 9, 14}, {2, 9, 13, 14, 15}
{2, 9, 15} → {4, 9, 15}, {5, 9, 15}, {2, 15}, {2, 9, 12}, {2, 9}, {2, 9, 13, 15}, {2, 9, 14, 15}
{2, 12} → {4, 12}, {5, 12}, {2, 14}, {2, 15}, {2, 3, 12}, {2, 9, 12}, {2, 12, 13}
{2, 12, 13} → {4, 12, 13}, {5, 12, 13}, {2, 13, 14}, {2, 13, 15}, {2, 12}, {2, 3, 12, 13}, {2, 9, 12, 13}
{2, 13} → {0, 13}, {4, 13}, {5, 13}, {2}, {2, 3, 13}, {2, 9, 13}, {2, 13, 14}, {2, 13, 15}
{2, 13, 14} → {0, 13, 14}, {4, 13, 14}, {5, 13, 14}, {2, 14}, {2, 12, 13}, {2, 13}, {2, 3, 13, 14}, {2, 9, 13, 14}, {2, 13, 14, 15}
{2, 13, 14, 15} → {0, 13, 14, 15}, {4, 13, 14, 15}, {5, 13, 14, 15}, {2, 14, 15}, {2, 13, 15}, {2, 13, 14}, {2, 3, 13, 14, 15}, {2, 9, 13, 14, 15}
{2, 13, 15} → {0, 13, 15}, {4, 13, 15}, {5, 13, 15}, {2, 15}, {2, 12, 13}, {2, 13}, {2, 3, 13, 15}, {2, 9, 13, 15}, {2, 13, 14, 15}
{2, 14} → {0, 14}, {4, 14}, {5, 14}, {2, 12}, {2}, {2, 3, 14}, {2, 9, 14}, {2, 13, 14}, {2, 14, 15}
{2, 14, 15} → {0, 14, 15}, {4, 14, 15}, {5, 14, 15}, {2, 15}, {2, 14}, {2, 3, 14, 15}, {2, 9, 14, 15}, {2, 13, 14, 15}
{2, 15} → {0, 15}, {4, 15}, {5, 15}, {2, 12}, {2}, {2, 3, 15}, {2, 9, 15}, {2, 13, 15}, {2, 14, 15}
{3, 4} → {1, 4}, {4}, {2, 3}, {3}, {3, 4, 5}, {3, 4, 8}, {3, 4, 13}, {3, 4, 14}, {3, 4, 15}
{3, 4, 5} → {4, 5}, {3, 5}, {3, 4}, {3, 4, 5, 13}, {3, 4, 5, 14}, {3, 4, 5, 15}
{3, 4, 5, 10} → {4, 5, 10}, {3, 5, 10}, {3, 4, 10}, {3, 4, 5, 11}, {3, 4, 5, 12}
{3, 4, 5, 11} → {4, 5, 11}, {3, 5, 11}, {3, 4, 11}, {3, 4, 5, 10}, {3, 4, 5, 13}, {3, 4, 5, 15}, {3, 4, 5, 11, 14}
{3, 4, 5, 11, 14} → {4, 5, 11, 14}, {3, 5, 11, 14}, {3, 4, 11, 14}, {3, 4, 5, 13, 14}, {3, 4, 5, 14, 15}, {3, 4, 5, 11}
{3, 4, 5, 12} → {4, 5, 12}, {3, 5, 12}, {3, 4, 12}, {3, 4, 5, 10}, {3, 4, 5, 14}, {3, 4, 5, 15}, {3, 4, 5, 12, 13}
{3, 4, 5, 12, 13} → {4, 5, 12, 13}, {3, 5, 12, 13}, {3, 4, 12, 13}, {3, 4, 5, 13, 14}, {3, 4, 5, 13, 15}, {3, 4, 5, 12}
{3, 4, 5, 13} → {4, 5, 13}, {3, 5, 13}, {3, 4, 13}, {3, 4, 5, 11}, {3, 4, 5}, {3, 4, 5, 13, 14}, {3, 4, 5, 13, 15}
{3, 4, 5, 13, 14} → {4, 5, 13, 14}, {3, 5, 13, 14}, {3, 4, 13, 14}, {3, 4, 5, 11, 14}, {3, 4, 5, 14}, {3, 4, 5, 12, 13}, {3, 4, 5, 13}, {3, 4, 5, 13, 14, 15}
{3, 4, 5, 13, 14, 15} → {4, 5, 13, 14, 15}, {3, 5, 13, 14, 15}, {3, 4, 13, 14, 15}, {3, 4, 5, 14, 15}, {3, 4, 5, 13, 15}, {3, 4, 5, 13, 14}
{3, 4, 5, 13, 15} → {4, 5, 13, 15}, {3, 5, 13, 15}, {3, 4, 13, 15}, {3, 4, 5, 15}, {3, 4, 5, 12, 13}, {3, 4, 5, 13}, {3, 4, 5, 13, 14, 15}
{3, 4, 5, 14} → {4, 5, 14}, {3, 5, 14}, {3, 4, 14}, {3, 4, 5, 12}, {3, 4, 5}, {3, 4, 5, 13, 14}, {3, 4, 5, 14, 15}
{3, 4, 5, 14, 15} → {4, 5, 14, 15}, {3, 5, 14, 15}, {3, 4, 14, 15}, {3, 4, 5, 15}, {3, 4, 5, 11, 14}, {3, 4, 5, 14}, {3, 4, 5, 13, 14, 15}
{3, 4, 5, 15} → {4, 5, 15}, {3, 5, 15}, {3, 4, 15}, {3, 4, 5, 11}, {3, 4, 5, 12}, {3, 4, 5}, {3, 4, 5, 13, 15}, {3, 4, 5, 14, 15}
{3, 4, 8} → {4, 8}, {3, 8}, {3, 4}, {3, 4, 8, 13}, {3, 4, 8, 14}, {3, 4, 8, 15}
{3, 4, 8, 10} → {4, 8, 10}, {3, 8, 10}, {3, 4, 10}, {3, 4, 8, 11}, {3, 4, 8, 12}
{3, 4, 8, 11} → {4, 8, 11}, {3, 8, 11}, {3, 4, 11}, {3, 4, 8, 10}, {3, 4, 8, 13}, {3, 4, 8, 15}, {3, 4, 8, 11, 14}
{3, 4, 8, 11, 14} → {4, 8, 11, 14}, {3, 8, 11, 14}, {3, 4, 11, 14}, {3, 4, 8, 13, 14}, {3, 4, 8, 14, 15}, {3, 4, 8, 11}
{3, 4, 8, 12} → {4, 8, 12}, {3, 8, 12}, {3, 4, 12}, {3, 4, 8, 10}, {3, 4, 8, 14}, {3, 4, 8, 15}, {3, 4, 8, 12, 13}
{3, 4, 8, 12, 13} → {4, 8, 12, 13}, {3, 8, 12, 13}, {3, 4, 12, 13}, {3, 4, 8, 13, 14}, {3, 4, 8, 13, 15}, {3, 4, 8, 12}
{3, 4, 8, 13} → {4, 8, 13}, {3, 8, 13}, {3, 4, 13}, {3, 4, 8, 11}, {3, 4, 8}, {3, 4, 8, 13, 14}, {3, 4, 8, 13, 15}
{3, 4, 8, 13, 14} → {4, 8, 13, 14}, {3, 8, 13, 14}, {3, 4, 13, 14}, {3, 4, 8, 11, 14}, {3, 4, 8, 14}, {3, 4, 8, 12, 13}, {3, 4, 8, 13}, {3, 4, 8, 13, 14, 15}
{3, 4, 8, 13, 14, 15} → {4, 8, 13, 14, 15}, {3, 8, 13, 14, 15}, {3, 4, 13, 14, 15}, {3, 4, 8, 14, 15}, {3, 4, 8, 13, 15}, {3, 4, 8, 13, 14}
{3, 4, 8, 13, 15} → {4, 8, 13, 15}, {3, 8, 13, 15}, {3, 4, 13, 15}, {3, 4, 8, 15}, {3, 4, 8, 12, 13}, {3, 4, 8, 13}, {3, 4, 8, 13, 14, 15}
{3, 4, 8, 14} → {4, 8, 14}, {3, 8, 14}, {3, 4, 14}, {3, 4, 8, 12}, {3, 4, 8}, {3, 4, 8, 13, 14}, {3, 4, 8, 14, 15}
{3, 4, 8, 14, 15} → {4, 8, 14, 15}, {3, 8, 14, 15}, {3, 4, 14, 15}, {3, 4, 8, 15}, {3, 4, 8, 11, 14}, {3, 4, 8, 14}, {3, 4, 8, 13, 14, 15}
{3, 4, 8, 15} → {4, 8, 15}, {3, 8, 15}, {3, 4, 15}, {3, 4, 8, 11}, {3, 4, 8, 12}, {3, 4, 8}, {3, 4, 8, 13, 15}, {3, 4, 8, 14, 15}
{3, 4, 10} → {4, 10}, {3, 10}, {3, 4, 11}, {3, 4, 12}, {3, 4, 5, 10}, {3, 4, 8, 10}

{3, 4, 11} → {1, 4, 11}, {4, 11}, {3, 11}, {3, 4, 10}, {3, 4, 13}, {3, 4, 15}, {3, 4, 5, 11}, {3, 4, 8, 11}, {3, 4, 11, 14}
{3, 4, 11, 14} → {1, 4, 11, 14}, {4, 11, 14}, {3, 11, 14}, {3, 4, 13, 14}, {3, 4, 14, 15}, {3, 4, 11}, {3, 4, 5, 11, 14}, {3, 4, 8, 11, 14}
{3, 4, 12} → {4, 12}, {2, 3, 12}, {3, 12}, {3, 4, 10}, {3, 4, 14}, {3, 4, 15}, {3, 4, 5, 12}, {3, 4, 8, 12}, {3, 4, 12, 13}
{3, 4, 12, 13} → {4, 12, 13}, {2, 3, 12, 13}, {3, 12, 13}, {3, 4, 13, 14}, {3, 4, 13, 15}, {3, 4, 12}, {3, 4, 5, 12, 13}, {3, 4, 8, 12, 13}
{3, 4, 13} → {1, 4, 13}, {4, 13}, {2, 3, 13}, {3, 13}, {3, 4, 11}, {3, 4}, {3, 4, 5, 13}, {3, 4, 8, 13}, {3, 4, 13, 14}, {3, 4, 13, 15}
{3, 4, 13, 14} → {1, 4, 13, 14}, {4, 13, 14}, {2, 3, 13, 14}, {3, 13, 14}, {3, 4, 11, 14}, {3, 4, 14}, {3, 4, 12, 13}, {3, 4, 13}, {3, 4, 5, 13, 14}, {3, 4, 8, 13, 14}, {3, 4, 13, 14, 15}
{3, 4, 13, 14, 15} → {1, 4, 13, 14, 15}, {4, 13, 14, 15}, {2, 3, 13, 14, 15}, {3, 13, 14, 15}, {3, 4, 14, 15}, {3, 4, 13, 15}, {3, 4, 13, 14}, {3, 4, 5, 13, 14, 15}, {3, 4, 8, 13, 14, 15}
{3, 4, 13, 15} → {1, 4, 13, 15}, {4, 13, 15}, {2, 3, 13, 15}, {3, 13, 15}, {3, 4, 15}, {3, 4, 12, 13}, {3, 4, 13}, {3, 4, 5, 13, 15}, {3, 4, 8, 13, 15}, {3, 4, 13, 14, 15}
{3, 4, 14} → {1, 4, 14}, {4, 14}, {2, 3, 14}, {3, 14}, {3, 4, 12}, {3, 4}, {3, 4, 5, 14}, {3, 4, 8, 14}, {3, 4, 13, 14}, {3, 4, 14, 15}
{3, 4, 14, 15} → {1, 4, 14, 15}, {4, 14, 15}, {2, 3, 14, 15}, {3, 14, 15}, {3, 4, 15}, {3, 4, 11, 14}, {3, 4, 14}, {3, 4, 5, 14, 15}, {3, 4, 8, 14, 15}, {3, 4, 13, 14, 15}
{3, 4, 15} → {1, 4, 15}, {4, 15}, {2, 3, 15}, {3, 15}, {3, 4, 11}, {3, 4, 12}, {3, 4}, {3, 4, 5, 15}, {3, 4, 8, 15}, {3, 4, 13, 15}, {3, 4, 14, 15}
{3, 5} → {5}, {2, 3}, {3}, {3, 4, 5}, {3, 5, 7}, {3, 5, 13}, {3, 5, 14}, {3, 5, 15}
{3, 5, 7} → {5, 7}, {3, 7}, {3, 5}, {3, 5, 7, 13}, {3, 5, 7, 14}, {3, 5, 7, 15}
{3, 5, 7, 10} → {5, 7, 10}, {3, 7, 10}, {3, 5, 10}, {3, 5, 7, 11}, {3, 5, 7, 12}
{3, 5, 7, 11} → {5, 7, 11}, {3, 7, 11}, {3, 5, 11}, {3, 5, 7, 10}, {3, 5, 7, 13}, {3, 5, 7, 15}, {3, 5, 7, 11, 14}
{3, 5, 7, 11, 14} → {5, 7, 11, 14}, {3, 7, 11, 14}, {3, 5, 11, 14}, {3, 5, 7, 13, 14}, {3, 5, 7, 14, 15}, {3, 5, 7, 11}
{3, 5, 7, 12} → {5, 7, 12}, {3, 7, 12}, {3, 5, 12}, {3, 5, 7, 10}, {3, 5, 7, 14}, {3, 5, 7, 15}, {3, 5, 7, 12, 13}
{3, 5, 7, 12, 13} → {5, 7, 12, 13}, {3, 7, 12, 13}, {3, 5, 12, 13}, {3, 5, 7, 13, 14}, {3, 5, 7, 13, 15}, {3, 5, 7, 12}
{3, 5, 7, 13} → {5, 7, 13}, {3, 7, 13}, {3, 5, 13}, {3, 5, 7, 11}, {3, 5, 7}, {3, 5, 7, 13, 14}, {3, 5, 7, 13, 15}
{3, 5, 7, 13, 14} → {5, 7, 13, 14}, {3, 7, 13, 14}, {3, 5, 13, 14}, {3, 5, 7, 11, 14}, {3, 5, 7, 14}, {3, 5, 7, 12, 13}, {3, 5, 7, 13}, {3, 5, 7, 13, 14, 15}
{3, 5, 7, 13, 14, 15} → {5, 7, 13, 14, 15}, {3, 7, 13, 14, 15}, {3, 5, 13, 14, 15}, {3, 5, 7, 14, 15}, {3, 5, 7, 13, 15}, {3, 5, 7, 13, 14}
{3, 5, 7, 13, 15} → {5, 7, 13, 15}, {3, 7, 13, 15}, {3, 5, 13, 15}, {3, 5, 7, 15}, {3, 5, 7, 12, 13}, {3, 5, 7, 13}, {3, 5, 7, 13, 14, 15}
{3, 5, 7, 14} → {5, 7, 14}, {3, 7, 14}, {3, 5, 14}, {3, 5, 7, 12}, {3, 5, 7}, {3, 5, 7, 13, 14}, {3, 5, 7, 14, 15}
{3, 5, 7, 14, 15} → {5, 7, 14, 15}, {3, 7, 14, 15}, {3, 5, 14, 15}, {3, 5, 7, 15}, {3, 5, 7, 11, 14}, {3, 5, 7, 14}, {3, 5, 7, 13, 14, 15}
{3, 5, 7, 15} → {5, 7, 15}, {3, 7, 15}, {3, 5, 15}, {3, 5, 7, 11}, {3, 5, 7, 12}, {3, 5, 7}, {3, 5, 7, 13, 15}, {3, 5, 7, 14, 15}
{3, 5, 10} → {5, 10}, {3, 10}, {3, 5, 11}, {3, 5, 12}, {3, 4, 5, 10}, {3, 5, 7, 10}
{3, 5, 11} → {5, 11}, {3, 11}, {3, 5, 10}, {3, 5, 13}, {3, 5, 15}, {3, 4, 5, 11}, {3, 5, 7, 11}, {3, 5, 11, 14}
{3, 5, 11, 14} → {5, 11, 14}, {3, 11, 14}, {3, 5, 13, 14}, {3, 5, 14, 15}, {3, 5, 11}, {3, 4, 5, 11, 14}, {3, 5, 7, 11, 14}
{3, 5, 12} → {5, 12}, {2, 3, 12}, {3, 12}, {3, 5, 10}, {3, 5, 14}, {3, 5, 15}, {3, 4, 5, 12}, {3, 5, 7, 12}, {3, 5, 12, 13}
{3, 5, 12, 13} → {5, 12, 13}, {2, 3, 12, 13}, {3, 12, 13}, {3, 5, 13, 14}, {3, 5, 13, 15}, {3, 5, 12}, {3, 4, 5, 12, 13}, {3, 5, 7, 12, 13}
{3, 5, 13} → {5, 13}, {2, 3, 13}, {3, 13}, {3, 5, 11}, {3, 5}, {3, 4, 5, 13}, {3, 5, 7, 13}, {3, 5, 13, 14}, {3, 5, 13, 15}
{3, 5, 13, 14} → {5, 13, 14}, {2, 3, 13, 14}, {3, 13, 14}, {3, 5, 11, 14}, {3, 5, 14}, {3, 5, 12, 13}, {3, 5, 13}, {3, 4, 5, 13, 14}, {3, 5, 7, 13, 14}, {3, 5, 13, 14, 15}
{3, 5, 13, 14, 15} → {5, 13, 14, 15}, {2, 3, 13, 14, 15}, {3, 13, 14, 15}, {3, 5, 14, 15}, {3, 5, 13, 15}, {3, 5, 13, 14}, {3, 4, 5, 13, 14, 15}, {3, 5, 7, 13, 14, 15}
{3, 5, 13, 15} → {5, 13, 15}, {2, 3, 13, 15}, {3, 13, 15}, {3, 5, 15}, {3, 5, 12, 13}, {3, 5, 13}, {3, 4, 5, 13, 15}, {3, 5, 7, 13, 15}, {3, 5, 13, 14, 15}
{3, 5, 14} → {5, 14}, {2, 3, 14}, {3, 14}, {3, 5, 12}, {3, 5}, {3, 4, 5, 14}, {3, 5, 7, 14}, {3, 5, 13, 14}, {3, 5, 14, 15}
{3, 5, 14, 15} → {5, 14, 15}, {2, 3, 14, 15}, {3, 14, 15}, {3, 5, 15}, {3, 5, 11, 14}, {3, 5, 14}, {3, 4, 5, 14, 15}, {3, 5, 7, 14, 15}, {3, 5, 13, 14, 15}
{3, 5, 15} → {5, 15}, {2, 3, 15}, {3, 15}, {3, 5, 11}, {3, 5, 12}, {3, 5}, {3, 4, 5, 15}, {3, 5, 7, 15}, {3, 5, 13, 15}, {3, 5, 14, 15}
{3, 6} → {6}, {3, 7}, {3, 8}, {3, 6, 13}, {3, 6, 14}, {3, 6, 15}
{3, 6, 12} → {6, 12}, {3, 7, 12}, {3, 8, 12}, {3, 6, 14}, {3, 6, 15}, {3, 6, 12, 13}
{3, 6, 12, 13} → {6, 12, 13}, {3, 7, 12, 13}, {3, 8, 12, 13}, {3, 6, 13, 14}, {3, 6, 13, 15}, {3, 6, 12}
{3, 6, 13} → {6, 13}, {3, 7, 13}, {3, 8, 13}, {3, 6}, {3, 6, 13, 14}, {3, 6, 13, 15}
{3, 6, 13, 14} → {6, 13, 14}, {3, 7, 13, 14}, {3, 8, 13, 14}, {3, 6, 14}, {3, 6, 12, 13}, {3, 6, 13}, {3, 6, 13, 14, 15}
{3, 6, 13, 14, 15} → {6, 13, 14, 15}, {3, 7, 13, 14, 15}, {3, 8, 13, 14, 15}, {3, 6, 14, 15}, {3, 6, 13, 15}, {3, 6, 13, 14}
{3, 6, 13, 15} → {6, 13, 15}, {3, 7, 13, 15}, {3, 8, 13, 15}, {3, 6, 15}, {3, 6, 12, 13}, {3, 6, 13}, {3, 6, 13, 14, 15}
{3, 6, 14} → {6, 14}, {3, 7, 14}, {3, 8, 14}, {3, 6, 12}, {3, 6}, {3, 6, 13, 14}, {3, 6, 14, 15}
{3, 6, 14, 15} → {6, 14, 15}, {3, 7, 14, 15}, {3, 8, 14, 15}, {3, 6, 15}, {3, 6, 14}, {3, 6, 13, 14, 15}
{3, 6, 15} → {6, 15}, {3, 7, 15}, {3, 8, 15}, {3, 6, 12}, {3, 6}, {3, 6, 13, 15}, {3, 6, 14, 15}
{3, 7} → {1, 7}, {7}, {3, 6}, {3}, {3, 5, 7}, {3, 7, 8}, {3, 7, 13}, {3, 7, 14}, {3, 7, 15}
{3, 7, 8} → {7, 8}, {3, 8}, {3, 7}, {3, 7, 8, 13}, {3, 7, 8, 14}, {3, 7, 8, 15}
{3, 7, 8, 10} → {7, 8, 10}, {3, 8, 10}, {3, 7, 10}, {3, 7, 8, 11}, {3, 7, 8, 12}
{3, 7, 8, 11} → {7, 8, 11}, {3, 8, 11}, {3, 7, 11}, {3, 7, 8, 10}, {3, 7, 8, 13}, {3, 7, 8, 15}, {3, 7, 8, 11, 14}
{3, 7, 8, 11, 14} → {7, 8, 11, 14}, {3, 8, 11, 14}, {3, 7, 11, 14}, {3, 7, 8, 13, 14}, {3, 7, 8, 14, 15}, {3, 7, 8, 11}
{3, 7, 8, 12} → {7, 8, 12}, {3, 8, 12}, {3, 7, 12}, {3, 7, 8, 10}, {3, 7, 8, 14}, {3, 7, 8, 15}, {3, 7, 8, 12, 13}
{3, 7, 8, 12, 13} → {7, 8, 12, 13}, {3, 8, 12, 13}, {3, 7, 12, 13}, {3, 7, 8, 13, 14}, {3, 7, 8, 13, 15}, {3, 7, 8, 12}
{3, 7, 8, 13} → {7, 8, 13}, {3, 8, 13}, {3, 7, 13}, {3, 7, 8, 11}, {3, 7, 8}, {3, 7, 8, 13, 14}, {3, 7, 8, 13, 15}
{3, 7, 8, 13, 14} → {7, 8, 13, 14}, {3, 8, 13, 14}, {3, 7, 13, 14}, {3, 7, 8, 11, 14}, {3, 7, 8, 14}, {3, 7, 8, 12, 13}, {3, 7, 8, 13}, {3, 7, 8, 13, 14, 15}
{3, 7, 8, 13, 14, 15} → {7, 8, 13, 14, 15}, {3, 8, 13, 14, 15}, {3, 7, 13, 14, 15}, {3, 7, 8, 14, 15}, {3, 7, 8, 13, 15}, {3, 7, 8, 13, 14}
{3, 7, 8, 13, 15} → {7, 8, 13, 15}, {3, 8, 13, 15}, {3, 7, 13, 15}, {3, 7, 8, 15}, {3, 7, 8, 12, 13}, {3, 7, 8, 13}, {3, 7, 8, 13, 14, 15}
{3, 7, 8, 14} → {7, 8, 14}, {3, 8, 14}, {3, 7, 14}, {3, 7, 8, 12}, {3, 7, 8}, {3, 7, 8, 13, 14}, {3, 7, 8, 14, 15}
{3, 7, 8, 14, 15} → {7, 8, 14, 15}, {3, 8, 14, 15}, {3, 7, 14, 15}, {3, 7, 8, 15}, {3, 7, 8, 11, 14}, {3, 7, 8, 14}, {3, 7, 8, 13, 14, 15}
{3, 7, 8, 15} → {7, 8, 15}, {3, 8, 15}, {3, 7, 15}, {3, 7, 8, 11}, {3, 7, 8, 12}, {3, 7, 8}, {3, 7, 8, 13, 15}, {3, 7, 8, 14, 15}
{3, 7, 10} → {7, 10}, {3, 10}, {3, 7, 11}, {3, 7, 12}, {3, 5, 7, 10}, {3, 7, 8, 10}
{3, 7, 11} → {1, 7, 11}, {7, 11}, {3, 11}, {3, 7, 10}, {3, 7, 13}, {3, 7, 15}, {3, 5, 7, 11}, {3, 7, 8, 11}, {3, 7, 11, 14}
{3, 7, 11, 14} → {1, 7, 11, 14}, {7, 11, 14}, {3, 11, 14}, {3, 7, 13, 14}, {3, 7, 14, 15}, {3, 7, 11}, {3, 5, 7, 11, 14}, {3, 7, 8, 11, 14}
{3, 7, 12} → {7, 12}, {3, 6, 12}, {3, 12}, {3, 7, 10}, {3, 7, 14}, {3, 7, 15}, {3, 5, 7, 12}, {3, 7, 8, 12}, {3, 7, 12, 13}
{3, 7, 12, 13} → {7, 12, 13}, {3, 6, 12, 13}, {3, 12, 13}, {3, 7, 13, 14}, {3, 7, 13, 15}, {3, 7, 12}, {3, 5, 7, 12, 13}, {3, 7, 8, 12, 13}
{3, 7, 13} → {1, 7, 13}, {7, 13}, {3, 6, 13}, {3, 13}, {3, 7, 11}, {3, 7}, {3, 5, 7, 13}, {3, 7, 8, 13}, {3, 7, 13, 14}, {3, 7, 13, 15}
{3, 7, 13, 14} → {1, 7, 13, 14}, {7, 13, 14}, {3, 6, 13, 14}, {3, 13, 14}, {3, 7, 11, 14}, {3, 7, 14}, {3, 7, 12, 13}, {3, 7, 13}, {3, 5, 7, 13, 14}, {3, 7, 8, 13, 14}, {3, 7, 13, 14, 15}
{3, 7, 13, 14, 15} → {1, 7, 13, 14, 15}, {7, 13, 14, 15}, {3, 6, 13, 14, 15}, {3, 13, 14, 15}, {3, 7, 14, 15}, {3, 7, 13, 15}, {3, 7, 13, 14}, {3, 5, 7, 13, 14, 15}, {3, 7, 8, 13, 14, 15}

{3, 7, 13, 15} → {1, 7, 13, 15}, {7, 13, 15}, {3, 6, 13, 15}, {3, 13, 15}, {3, 7, 15}, {3, 7, 12, 13}, {3, 7, 13}, {3, 5, 7, 13, 15}, {3, 7, 8, 13, 15}, {3, 7, 13, 14, 15}
{3, 7, 14} → {1, 7, 14}, {7, 14}, {3, 6, 14}, {3, 14}, {3, 7, 12}, {3, 7}, {3, 5, 7, 14}, {3, 7, 8, 14}, {3, 7, 13, 14}, {3, 7, 14, 15}
{3, 7, 14, 15} → {1, 7, 14, 15}, {7, 14, 15}, {3, 6, 14, 15}, {3, 14, 15}, {3, 7, 15}, {3, 7, 11, 14}, {3, 7, 14}, {3, 5, 7, 14, 15}, {3, 7, 8, 14, 15}, {3, 7, 13, 14, 15}
{3, 7, 15} → {1, 7, 15}, {7, 15}, {3, 6, 15}, {3, 15}, {3, 7, 11}, {3, 7, 12}, {3, 7}, {3, 5, 7, 15}, {3, 7, 8, 15}, {3, 7, 13, 15}, {3, 7, 14, 15}
{3, 8} → {8}, {3, 6}, {3}, {3, 4, 8}, {3, 7, 8}, {3, 8, 13}, {3, 8, 14}, {3, 8, 15}
{3, 8, 10} → {8, 10}, {3, 10}, {3, 8, 11}, {3, 8, 12}, {3, 4, 8, 10}, {3, 7, 8, 10}
{3, 8, 11} → {8, 11}, {3, 11}, {3, 8, 10}, {3, 8, 13}, {3, 8, 15}, {3, 4, 8, 11}, {3, 7, 8, 11}, {3, 8, 11, 14}
{3, 8, 11, 14} → {8, 11, 14}, {3, 11, 14}, {3, 8, 13, 14}, {3, 8, 14, 15}, {3, 8, 11}, {3, 4, 8, 11, 14}, {3, 7, 8, 11, 14}
{3, 8, 12} → {8, 12}, {3, 6, 12}, {3, 12}, {3, 8, 10}, {3, 8, 14}, {3, 8, 15}, {3, 4, 8, 12}, {3, 7, 8, 12}, {3, 8, 12, 13}
{3, 8, 12, 13} → {8, 12, 13}, {3, 6, 12, 13}, {3, 12, 13}, {3, 8, 13, 14}, {3, 8, 13, 15}, {3, 8, 12}, {3, 4, 8, 12, 13}, {3, 7, 8, 12, 13}
{3, 8, 13} → {8, 13}, {3, 6, 13}, {3, 13}, {3, 8, 11}, {3, 8}, {3, 4, 8, 13}, {3, 7, 8, 13}, {3, 8, 13, 14}, {3, 8, 13, 15}
{3, 8, 13, 14} → {8, 13, 14}, {3, 6, 13, 14}, {3, 13, 14}, {3, 8, 11, 14}, {3, 8, 14}, {3, 8, 12, 13}, {3, 8, 13}, {3, 4, 8, 13, 14}, {3, 7, 8, 13, 14}, {3, 8, 13, 14, 15}
{3, 8, 13, 14, 15} → {8, 13, 14, 15}, {3, 6, 13, 14, 15}, {3, 13, 14, 15}, {3, 8, 14, 15}, {3, 8, 13, 15}, {3, 8, 13, 14}, {3, 4, 8, 13, 14, 15}, {3, 7, 8, 13, 14, 15}
{3, 8, 13, 15} → {8, 13, 15}, {3, 6, 13, 15}, {3, 13, 15}, {3, 8, 15}, {3, 8, 12, 13}, {3, 8, 13}, {3, 4, 8, 13, 15}, {3, 7, 8, 13, 15}, {3, 8, 13, 14, 15}
{3, 8, 14} → {8, 14}, {3, 6, 14}, {3, 14}, {3, 8, 12}, {3, 8}, {3, 4, 8, 14}, {3, 7, 8, 14}, {3, 8, 13, 14}, {3, 8, 14, 15}
{3, 8, 14, 15} → {8, 14, 15}, {3, 6, 14, 15}, {3, 14, 15}, {3, 8, 15}, {3, 8, 11, 14}, {3, 8, 14}, {3, 4, 8, 14, 15}, {3, 7, 8, 14, 15}, {3, 8, 13, 14, 15}
{3, 8, 15} → {8, 15}, {3, 6, 15}, {3, 15}, {3, 8, 11}, {3, 8, 12}, {3, 8}, {3, 4, 8, 15}, {3, 7, 8, 15}, {3, 8, 13, 15}, {3, 8, 14, 15}
{3, 10} → {10}, {3, 11}, {3, 12}, {3, 4, 10}, {3, 5, 10}, {3, 7, 10}, {3, 8, 10}
{3, 11} → {1, 11}, {11}, {3, 10}, {3, 13}, {3, 15}, {3, 4, 11}, {3, 5, 11}, {3, 7, 11}, {3, 8, 11}, {3, 11, 14}
{3, 11, 14} → {1, 11, 14}, {11, 14}, {3, 13, 14}, {3, 14, 15}, {3, 11}, {3, 4, 11, 14}, {3, 5, 11, 14}, {3, 7, 11, 14}, {3, 8, 11, 14}
{3, 12} → {12}, {3, 10}, {3, 14}, {3, 15}, {3, 4, 12}, {3, 5, 12}, {3, 7, 12}, {3, 8, 12}, {3, 12, 13}
{3, 12, 13} → {12, 13}, {3, 13, 14}, {3, 13, 15}, {3, 12}, {3, 4, 12, 13}, {3, 5, 12, 13}, {3, 7, 12, 13}, {3, 8, 12, 13}
{3, 13} → {1, 13}, {13}, {3, 11}, {3}, {3, 4, 13}, {3, 5, 13}, {3, 7, 13}, {3, 8, 13}, {3, 13, 14}, {3, 13, 15}
{3, 13, 14} → {1, 13, 14}, {13, 14}, {3, 11, 14}, {3, 14}, {3, 12, 13}, {3, 13}, {3, 4, 13, 14}, {3, 5, 13, 14}, {3, 7, 13, 14}, {3, 8, 13, 14}, {3, 13, 14, 15}
{3, 13, 14, 15} → {1, 13, 14, 15}, {13, 14, 15}, {3, 14, 15}, {3, 13, 15}, {3, 13, 14}, {3, 4, 13, 14, 15}, {3, 5, 13, 14, 15}, {3, 7, 13, 14, 15}, {3, 8, 13, 14, 15}
{3, 13, 15} → {1, 13, 15}, {13, 15}, {3, 15}, {3, 12, 13}, {3, 13}, {3, 4, 13, 15}, {3, 5, 13, 15}, {3, 7, 13, 15}, {3, 8, 13, 15}, {3, 13, 14, 15}
{3, 14} → {1, 14}, {14}, {3, 12}, {3}, {3, 4, 14}, {3, 5, 14}, {3, 7, 14}, {3, 8, 14}, {3, 13, 14}, {3, 14, 15}
{3, 14, 15} → {1, 14, 15}, {14, 15}, {3, 15}, {3, 11, 14}, {3, 14}, {3, 4, 14, 15}, {3, 5, 14, 15}, {3, 7, 14, 15}, {3, 8, 14, 15}, {3, 13, 14, 15}
{3, 15} → {1, 15}, {15}, {3, 11}, {3, 12}, {3}, {3, 4, 15}, {3, 5, 15}, {3, 7, 15}, {3, 8, 15}, {3, 13, 15}, {3, 14, 15}
{4, 5} → {5}, {1, 4}, {4}, {3, 4, 5}, {4, 5, 9}, {4, 5, 13}, {4, 5, 14}, {4, 5, 15}
{4, 5, 9} → {5, 9}, {4, 9}, {4, 5}, {4, 5, 9, 13}, {4, 5, 9, 14}, {4, 5, 9, 15}
{4, 5, 9, 10} → {5, 9, 10}, {4, 9, 10}, {4, 5, 10}, {4, 5, 9, 11}, {4, 5, 9, 12}
{4, 5, 9, 11} → {5, 9, 11}, {4, 9, 11}, {4, 5, 11}, {4, 5, 9, 10}, {4, 5, 9, 13}, {4, 5, 9, 15}, {4, 5, 9, 11, 14}
{4, 5, 9, 11, 14} → {5, 9, 11, 14}, {4, 9, 11, 14}, {4, 5, 11, 14}, {4, 5, 9, 13, 14}, {4, 5, 9, 14, 15}, {4, 5, 9, 11}
{4, 5, 9, 12} → {5, 9, 12}, {4, 9, 12}, {4, 5, 12}, {4, 5, 9, 10}, {4, 5, 9, 14}, {4, 5, 9, 15}, {4, 5, 9, 12, 13}
{4, 5, 9, 12, 13} → {5, 9, 12, 13}, {4, 9, 12, 13}, {4, 5, 12, 13}, {4, 5, 9, 13, 14}, {4, 5, 9, 13, 15}, {4, 5, 9, 12}
{4, 5, 9, 13} → {5, 9, 13}, {4, 9, 13}, {4, 5, 13}, {4, 5, 9, 11}, {4, 5, 9}, {4, 5, 9, 13, 14}, {4, 5, 9, 13, 15}
{4, 5, 9, 13, 14} → {5, 9, 13, 14}, {4, 9, 13, 14}, {4, 5, 13, 14}, {4, 5, 9, 11, 14}, {4, 5, 9, 14}, {4, 5, 9, 12, 13}, {4, 5, 9, 13}, {4, 5, 9, 13, 14, 15}
{4, 5, 9, 13, 14, 15} → {5, 9, 13, 14, 15}, {4, 9, 13, 14, 15}, {4, 5, 13, 14, 15}, {4, 5, 9, 14, 15}, {4, 5, 9, 13, 15}, {4, 5, 9, 13, 14}
{4, 5, 9, 13, 15} → {5, 9, 13, 15}, {4, 9, 13, 15}, {4, 5, 13, 15}, {4, 5, 9, 15}, {4, 5, 9, 12, 13}, {4, 5, 9, 13}, {4, 5, 9, 13, 14, 15}
{4, 5, 9, 14} → {5, 9, 14}, {4, 9, 14}, {4, 5, 14}, {4, 5, 9, 12}, {4, 5, 9}, {4, 5, 9, 13, 14}, {4, 5, 9, 14, 15}
{4, 5, 9, 14, 15} → {5, 9, 14, 15}, {4, 9, 14, 15}, {4, 5, 14, 15}, {4, 5, 9, 15}, {4, 5, 9, 11, 14}, {4, 5, 9, 14}, {4, 5, 9, 13, 14, 15}
{4, 5, 9, 15} → {5, 9, 15}, {4, 9, 15}, {4, 5, 15}, {4, 5, 9, 11}, {4, 5, 9, 12}, {4, 5, 9}, {4, 5, 9, 13, 15}, {4, 5, 9, 14, 15}
{4, 5, 10} → {5, 10}, {4, 10}, {4, 5, 11}, {4, 5, 12}, {3, 4, 5, 10}, {4, 5, 9, 10}
{4, 5, 11} → {5, 11}, {1, 4, 11}, {4, 11}, {4, 5, 10}, {4, 5, 13}, {4, 5, 15}, {3, 4, 5, 11}, {4, 5, 9, 11}, {4, 5, 11, 14}
{4, 5, 11, 14} → {5, 11, 14}, {1, 4, 11, 14}, {4, 11, 14}, {4, 5, 13, 14}, {4, 5, 14, 15}, {4, 5, 11}, {3, 4, 5, 11, 14}, {4, 5, 9, 11, 14}
{4, 5, 12} → {5, 12}, {4, 12}, {4, 5, 10}, {4, 5, 14}, {4, 5, 15}, {3, 4, 5, 12}, {4, 5, 9, 12}, {4, 5, 12, 13}
{4, 5, 12, 13} → {5, 12, 13}, {4, 12, 13}, {4, 5, 13, 14}, {4, 5, 13, 15}, {4, 5, 12}, {3, 4, 5, 12, 13}, {4, 5, 9, 12, 13}
{4, 5, 13} → {5, 13}, {1, 4, 13}, {4, 13}, {4, 5, 11}, {4, 5}, {3, 4, 5, 13}, {4, 5, 9, 13}, {4, 5, 13, 14}, {4, 5, 13, 15}
{4, 5, 13, 14} → {5, 13, 14}, {1, 4, 13, 14}, {4, 13, 14}, {4, 5, 11, 14}, {4, 5, 14}, {4, 5, 12, 13}, {4, 5, 13}, {3, 4, 5, 13, 14}, {4, 5, 9, 13, 14}, {4, 5, 13, 14, 15}
{4, 5, 13, 14, 15} → {5, 13, 14, 15}, {1, 4, 13, 14, 15}, {4, 13, 14, 15}, {4, 5, 14, 15}, {4, 5, 13, 15}, {4, 5, 13, 14}, {3, 4, 5, 13, 14, 15}, {4, 5, 9, 13, 14, 15}
{4, 5, 13, 15} → {5, 13, 15}, {1, 4, 13, 15}, {4, 13, 15}, {4, 5, 15}, {4, 5, 12, 13}, {4, 5, 13}, {3, 4, 5, 13, 15}, {4, 5, 9, 13, 15}, {4, 5, 13, 14, 15}
{4, 5, 14} → {5, 14}, {1, 4, 14}, {4, 14}, {4, 5, 12}, {4, 5}, {3, 4, 5, 14}, {4, 5, 9, 14}, {4, 5, 13, 14}, {4, 5, 14, 15}
{4, 5, 14, 15} → {5, 14, 15}, {1, 4, 14, 15}, {4, 14, 15}, {4, 5, 15}, {4, 5, 11, 14}, {4, 5, 14}, {3, 4, 5, 14, 15}, {4, 5, 9, 14, 15}, {4, 5, 13, 14, 15}
{4, 5, 15} → {5, 15}, {1, 4, 15}, {4, 15}, {4, 5, 11}, {4, 5, 12}, {4, 5}, {3, 4, 5, 15}, {4, 5, 9, 15}, {4, 5, 13, 15}, {4, 5, 14, 15}
{4, 8} → {8}, {4}, {3, 4, 8}, {4, 8, 9}, {4, 8, 13}, {4, 8, 14}, {4, 8, 15}
{4, 8, 9} → {8, 9}, {4, 9}, {4, 8}, {4, 8, 9, 13}, {4, 8, 9, 14}, {4, 8, 9, 15}
{4, 8, 9, 10} → {8, 9, 10}, {4, 9, 10}, {4, 8, 10}, {4, 8, 9, 11}, {4, 8, 9, 12}
{4, 8, 9, 11} → {8, 9, 11}, {4, 9, 11}, {4, 8, 11}, {4, 8, 9, 10}, {4, 8, 9, 13}, {4, 8, 9, 15}, {4, 8, 9, 11, 14}
{4, 8, 9, 11, 14} → {8, 9, 11, 14}, {4, 9, 11, 14}, {4, 8, 11, 14}, {4, 8, 9, 13, 14}, {4, 8, 9, 14, 15}, {4, 8, 9, 11}
{4, 8, 9, 12} → {8, 9, 12}, {4, 9, 12}, {4, 8, 12}, {4, 8, 9, 10}, {4, 8, 9, 14}, {4, 8, 9, 15}, {4, 8, 9, 12, 13}
{4, 8, 9, 12, 13} → {8, 9, 12, 13}, {4, 9, 12, 13}, {4, 8, 12, 13}, {4, 8, 9, 13, 14}, {4, 8, 9, 13, 15}, {4, 8, 9, 12}
{4, 8, 9, 13} → {8, 9, 13}, {4, 9, 13}, {4, 8, 13}, {4, 8, 9, 11}, {4, 8, 9}, {4, 8, 9, 13, 14}, {4, 8, 9, 13, 15}
{4, 8, 9, 13, 14} → {8, 9, 13, 14}, {4, 9, 13, 14}, {4, 8, 13, 14}, {4, 8, 9, 11, 14}, {4, 8, 9, 14}, {4, 8, 9, 12, 13}, {4, 8, 9, 13}, {4, 8, 9, 13, 14, 15}
{4, 8, 9, 13, 14, 15} → {8, 9, 13, 14, 15}, {4, 9, 13, 14, 15}, {4, 8, 13, 14, 15}, {4, 8, 9, 14, 15}, {4, 8, 9, 13, 15}, {4, 8, 9, 13, 14}

{4, 8, 9, 13, 15} → {8, 9, 13, 15}, {4, 9, 13, 15}, {4, 8, 13, 15}, {4, 8, 9, 15}, {4, 8, 9, 12, 13}, {4, 8, 9, 13}, {4, 8, 9, 13, 14, 15}
{4, 8, 9, 14} → {8, 9, 14}, {4, 9, 14}, {4, 8, 14}, {4, 8, 9, 12}, {4, 8, 9}, {4, 8, 9, 13, 14}, {4, 8, 9, 14, 15}
{4, 8, 9, 14, 15} → {8, 9, 14, 15}, {4, 9, 14, 15}, {4, 8, 14, 15}, {4, 8, 9, 15}, {4, 8, 9, 11, 14}, {4, 8, 9, 14}, {4, 8, 9, 13, 14, 15}
{4, 8, 9, 15} → {8, 9, 15}, {4, 9, 15}, {4, 8, 15}, {4, 8, 9, 11}, {4, 8, 9, 12}, {4, 8, 9}, {4, 8, 9, 13, 15}, {4, 8, 9, 14, 15}
{4, 8, 10} → {8, 10}, {4, 10}, {4, 8, 11}, {4, 8, 12}, {3, 4, 8, 10}, {4, 8, 9, 10}
{4, 8, 11} → {8, 11}, {4, 11}, {4, 8, 10}, {4, 8, 13}, {4, 8, 15}, {3, 4, 8, 11}, {4, 8, 9, 11}, {4, 8, 11, 14}
{4, 8, 11, 14} → {8, 11, 14}, {4, 11, 14}, {4, 8, 13, 14}, {4, 8, 14, 15}, {4, 8, 11}, {3, 4, 8, 11, 14}, {4, 8, 9, 11, 14}
{4, 8, 12} → {8, 12}, {4, 12}, {4, 8, 10}, {4, 8, 14}, {4, 8, 15}, {3, 4, 8, 12}, {4, 8, 9, 12}, {4, 8, 12, 13}
{4, 8, 12, 13} → {8, 12, 13}, {4, 12, 13}, {4, 8, 13, 14}, {4, 8, 13, 15}, {4, 8, 12}, {3, 4, 8, 12, 13}, {4, 8, 9, 12, 13}
{4, 8, 13} → {8, 13}, {4, 13}, {4, 8, 11}, {4, 8}, {3, 4, 8, 13}, {4, 8, 9, 13}, {4, 8, 13, 14}, {4, 8, 13, 15}
{4, 8, 13, 14} → {8, 13, 14}, {4, 13, 14}, {4, 8, 11, 14}, {4, 8, 14}, {4, 8, 12, 13}, {4, 8, 13}, {3, 4, 8, 13, 14}, {4, 8, 9, 13, 14}, {4, 8, 13, 14, 15}
{4, 8, 13, 14, 15} → {8, 13, 14, 15}, {4, 13, 14, 15}, {4, 8, 14, 15}, {4, 8, 13, 15}, {4, 8, 13, 14}, {3, 4, 8, 13, 14, 15}, {4, 8, 9, 13, 14, 15}
{4, 8, 13, 15} → {8, 13, 15}, {4, 13, 15}, {4, 8, 15}, {4, 8, 12, 13}, {4, 8, 13}, {3, 4, 8, 13, 15}, {4, 8, 9, 13, 15}, {4, 8, 13, 14, 15}
{4, 8, 14} → {8, 14}, {4, 14}, {4, 8, 12}, {4, 8}, {3, 4, 8, 14}, {4, 8, 9, 14}, {4, 8, 13, 14}, {4, 8, 14, 15}
{4, 8, 14, 15} → {8, 14, 15}, {4, 14, 15}, {4, 8, 15}, {4, 8, 11, 14}, {4, 8, 14}, {3, 4, 8, 14, 15}, {4, 8, 9, 14, 15}, {4, 8, 13, 14, 15}
{4, 8, 15} → {8, 15}, {4, 15}, {4, 8, 11}, {4, 8, 12}, {4, 8}, {3, 4, 8, 15}, {4, 8, 9, 15}, {4, 8, 13, 15}, {4, 8, 14, 15}
{4, 9} → {2, 9}, {9}, {4}, {4, 5, 9}, {4, 8, 9}, {4, 9, 13}, {4, 9, 14}, {4, 9, 15}
{4, 9, 10} → {9, 10}, {4, 10}, {4, 9, 11}, {4, 9, 12}, {4, 5, 9, 10}, {4, 8, 9, 10}
{4, 9, 11} → {9, 11}, {4, 11}, {4, 9, 10}, {4, 9, 13}, {4, 9, 15}, {4, 5, 9, 11}, {4, 8, 9, 11}, {4, 9, 11, 14}
{4, 9, 11, 14} → {9, 11, 14}, {4, 11, 14}, {4, 9, 13, 14}, {4, 9, 14, 15}, {4, 9, 11}, {4, 5, 9, 11, 14}, {4, 8, 9, 11, 14}
{4, 9, 12} → {2, 9, 12}, {9, 12}, {4, 12}, {4, 9, 10}, {4, 9, 14}, {4, 9, 15}, {4, 5, 9, 12}, {4, 8, 9, 12}, {4, 9, 12, 13}
{4, 9, 12, 13} → {2, 9, 12, 13}, {9, 12, 13}, {4, 12, 13}, {4, 9, 13, 14}, {4, 9, 13, 15}, {4, 9, 12}, {4, 5, 9, 12, 13}, {4, 8, 9, 12, 13}
{4, 9, 13} → {2, 9, 13}, {9, 13}, {4, 13}, {4, 9, 11}, {4, 9}, {4, 5, 9, 13}, {4, 8, 9, 13}, {4, 9, 13, 14}, {4, 9, 13, 15}
{4, 9, 13, 14} → {2, 9, 13, 14}, {9, 13, 14}, {4, 13, 14}, {4, 9, 11, 14}, {4, 9, 14}, {4, 9, 12, 13}, {4, 9, 13}, {4, 5, 9, 13, 14}, {4, 8, 9, 13, 14}, {4, 9, 13, 14, 15}
{4, 9, 13, 14, 15} → {2, 9, 13, 14, 15}, {9, 13, 14, 15}, {4, 13, 14, 15}, {4, 9, 14, 15}, {4, 9, 13, 15}, {4, 9, 13, 14}, {4, 5, 9, 13, 14, 15}, {4, 8, 9, 13, 14, 15}
{4, 9, 13, 15} → {2, 9, 13, 15}, {9, 13, 15}, {4, 13, 15}, {4, 9, 15}, {4, 9, 12, 13}, {4, 9, 13}, {4, 5, 9, 13, 15}, {4, 8, 9, 13, 15}, {4, 9, 13, 14, 15}
{4, 9, 14} → {2, 9, 14}, {9, 14}, {4, 14}, {4, 9, 12}, {4, 9}, {4, 5, 9, 14}, {4, 8, 9, 14}, {4, 9, 13, 14}, {4, 9, 14, 15}
{4, 9, 14, 15} → {2, 9, 14, 15}, {9, 14, 15}, {4, 14, 15}, {4, 9, 15}, {4, 9, 11, 14}, {4, 9, 14}, {4, 5, 9, 14, 15}, {4, 8, 9, 14, 15}, {4, 9, 13, 14, 15}
{4, 9, 15} → {2, 9, 15}, {9, 15}, {4, 15}, {4, 9, 11}, {4, 9, 12}, {4, 9}, {4, 5, 9, 15}, {4, 8, 9, 15}, {4, 9, 13, 15}, {4, 9, 14, 15}
{4, 10} → {10}, {4, 11}, {4, 12}, {3, 4, 10}, {4, 5, 10}, {4, 8, 10}, {4, 9, 10}
{4, 11} → {11}, {4, 10}, {4, 13}, {4, 15}, {3, 4, 11}, {4, 5, 11}, {4, 8, 11}, {4, 9, 11}, {4, 11, 14}
{4, 11, 14} → {11, 14}, {4, 13, 14}, {4, 14, 15}, {4, 11}, {3, 4, 11, 14}, {4, 5, 11, 14}, {4, 8, 11, 14}, {4, 9, 11, 14}
{4, 12} → {2, 12}, {12}, {4, 10}, {4, 14}, {4, 15}, {3, 4, 12}, {4, 5, 12}, {4, 8, 12}, {4, 9, 12}, {4, 12, 13}
{4, 12, 13} → {2, 12, 13}, {12, 13}, {4, 13, 14}, {4, 13, 15}, {4, 12}, {3, 4, 12, 13}, {4, 5, 12, 13}, {4, 8, 12, 13}, {4, 9, 12, 13}
{4, 13} → {2, 13}, {13}, {4, 11}, {4}, {3, 4, 13}, {4, 5, 13}, {4, 8, 13}, {4, 9, 13}, {4, 13, 14}, {4, 13, 15}
{4, 13, 14} → {2, 13, 14}, {13, 14}, {4, 11, 14}, {4, 14}, {4, 12, 13}, {4, 13}, {3, 4, 13, 14}, {4, 5, 13, 14}, {4, 8, 13, 14}, {4, 9, 13, 14}, {4, 13, 14, 15}
{4, 13, 14, 15} → {2, 13, 14, 15}, {13, 14, 15}, {4, 14, 15}, {4, 13, 15}, {4, 13, 14}, {3, 4, 13, 14, 15}, {4, 5, 13, 14, 15}, {4, 8, 13, 14, 15}, {4, 9, 13, 14, 15}
{4, 13, 15} → {2, 13, 15}, {13, 15}, {4, 15}, {4, 12, 13}, {4, 13}, {3, 4, 13, 15}, {4, 5, 13, 15}, {4, 8, 13, 15}, {4, 9, 13, 15}, {4, 13, 14, 15}
{4, 14} → {2, 14}, {14}, {4, 12}, {4}, {3, 4, 14}, {4, 5, 14}, {4, 8, 14}, {4, 9, 14}, {4, 13, 14}, {4, 14, 15}
{4, 14, 15} → {2, 14, 15}, {14, 15}, {4, 15}, {4, 11, 14}, {4, 14}, {3, 4, 14, 15}, {4, 5, 14, 15}, {4, 8, 14, 15}, {4, 9, 14, 15}, {4, 13, 14, 15}
{4, 15} → {2, 15}, {15}, {4, 11}, {4, 12}, {4}, {3, 4, 15}, {4, 5, 15}, {4, 8, 15}, {4, 9, 15}, {4, 13, 15}, {4, 14, 15}
{5, 7} → {1, 7}, {7}, {5}, {3, 5, 7}, {5, 7, 9}, {5, 7, 13}, {5, 7, 14}, {5, 7, 15}
{5, 7, 9} → {7, 9}, {5, 9}, {5, 7}, {5, 7, 9, 13}, {5, 7, 9, 14}, {5, 7, 9, 15}
{5, 7, 9, 10} → {7, 9, 10}, {5, 9, 10}, {5, 7, 10}, {5, 7, 9, 11}, {5, 7, 9, 12}
{5, 7, 9, 11} → {7, 9, 11}, {5, 9, 11}, {5, 7, 11}, {5, 7, 9, 10}, {5, 7, 9, 13}, {5, 7, 9, 15}, {5, 7, 9, 11, 14}
{5, 7, 9, 11, 14} → {7, 9, 11, 14}, {5, 9, 11, 14}, {5, 7, 11, 14}, {5, 7, 9, 13, 14}, {5, 7, 9, 14, 15}, {5, 7, 9, 11}
{5, 7, 9, 12} → {7, 9, 12}, {5, 9, 12}, {5, 7, 12}, {5, 7, 9, 10}, {5, 7, 9, 14}, {5, 7, 9, 15}, {5, 7, 9, 12, 13}
{5, 7, 9, 12, 13} → {7, 9, 12, 13}, {5, 9, 12, 13}, {5, 7, 12, 13}, {5, 7, 9, 13, 14}, {5, 7, 9, 13, 15}, {5, 7, 9, 12}
{5, 7, 9, 13} → {7, 9, 13}, {5, 9, 13}, {5, 7, 13}, {5, 7, 9, 11}, {5, 7, 9}, {5, 7, 9, 13, 14}, {5, 7, 9, 13, 15}
{5, 7, 9, 13, 14} → {7, 9, 13, 14}, {5, 9, 13, 14}, {5, 7, 13, 14}, {5, 7, 9, 11, 14}, {5, 7, 9, 14}, {5, 7, 9, 12, 13}, {5, 7, 9, 13}, {5, 7, 9, 13, 14, 15}
{5, 7, 9, 13, 14, 15} → {7, 9, 13, 14, 15}, {5, 9, 13, 14, 15}, {5, 7, 13, 14, 15}, {5, 7, 9, 14, 15}, {5, 7, 9, 13, 15}, {5, 7, 9, 13, 14}
{5, 7, 9, 13, 15} → {7, 9, 13, 15}, {5, 9, 13, 15}, {5, 7, 13, 15}, {5, 7, 9, 15}, {5, 7, 9, 12, 13}, {5, 7, 9, 13}, {5, 7, 9, 13, 14, 15}
{5, 7, 9, 14} → {7, 9, 14}, {5, 9, 14}, {5, 7, 14}, {5, 7, 9, 12}, {5, 7, 9}, {5, 7, 9, 13, 14}, {5, 7, 9, 14, 15}
{5, 7, 9, 14, 15} → {7, 9, 14, 15}, {5, 9, 14, 15}, {5, 7, 14, 15}, {5, 7, 9, 15}, {5, 7, 9, 11, 14}, {5, 7, 9, 14}, {5, 7, 9, 13, 14, 15}
{5, 7, 9, 15} → {7, 9, 15}, {5, 9, 15}, {5, 7, 15}, {5, 7, 9, 11}, {5, 7, 9, 12}, {5, 7, 9}, {5, 7, 9, 13, 15}, {5, 7, 9, 14, 15}
{5, 7, 10} → {7, 10}, {5, 10}, {5, 7, 11}, {5, 7, 12}, {3, 5, 7, 10}, {5, 7, 9, 10}
{5, 7, 11} → {1, 7, 11}, {7, 11}, {5, 11}, {5, 7, 10}, {5, 7, 13}, {5, 7, 15}, {3, 5, 7, 11}, {5, 7, 9, 11}, {5, 7, 11, 14}
{5, 7, 11, 14} → {1, 7, 11, 14}, {7, 11, 14}, {5, 11, 14}, {5, 7, 13, 14}, {5, 7, 14, 15}, {5, 7, 11}, {3, 5, 7, 11, 14}, {5, 7, 9, 11, 14}
{5, 7, 12} → {7, 12}, {5, 12}, {5, 7, 10}, {5, 7, 14}, {5, 7, 15}, {3, 5, 7, 12}, {5, 7, 9, 12}, {5, 7, 12, 13}
{5, 7, 12, 13} → {7, 12, 13}, {5, 12, 13}, {5, 7, 13, 14}, {5, 7, 13, 15}, {5, 7, 12}, {3, 5, 7, 12, 13}, {5, 7, 9, 12, 13}
{5, 7, 13} → {1, 7, 13}, {7, 13}, {5, 13}, {5, 7, 11}, {5, 7}, {3, 5, 7, 13}, {5, 7, 9, 13}, {5, 7, 13, 14}, {5, 7, 13, 15}
{5, 7, 13, 14} → {1, 7, 13, 14}, {7, 13, 14}, {5, 13, 14}, {5, 7, 11, 14}, {5, 7, 14}, {5, 7, 12, 13}, {5, 7, 13}, {3, 5, 7, 13, 14}, {5, 7, 9, 13, 14}, {5, 7, 13, 14, 15}
{5, 7, 13, 14, 15} → {1, 7, 13, 14, 15}, {7, 13, 14, 15}, {5, 13, 14, 15}, {5, 7, 14, 15}, {5, 7, 13, 15}, {5, 7, 13, 14}, {3, 5, 7, 13, 14, 15}, {5, 7, 9, 13, 14, 15}
{5, 7, 13, 15} → {1, 7, 13, 15}, {7, 13, 15}, {5, 13, 15}, {5, 7, 15}, {5, 7, 12, 13}, {5, 7, 13}, {3, 5, 7, 13, 15}, {5, 7, 9, 13, 15}, {5, 7, 13, 14, 15}
{5, 7, 14} → {1, 7, 14}, {7, 14}, {5, 14}, {5, 7, 12}, {5, 7}, {3, 5, 7, 14}, {5, 7, 9, 14}, {5, 7, 13, 14}, {5, 7, 14, 15}
{5, 7, 14, 15} → {1, 7, 14, 15}, {7, 14, 15}, {5, 14, 15}, {5, 7, 15}, {5, 7, 11, 14}, {5, 7, 14}, {3, 5, 7, 14, 15}, {5, 7, 9, 14, 15}, {5, 7, 13, 14, 15}
{5, 7, 15} → {1, 7, 15}, {7, 15}, {5, 15}, {5, 7, 11}, {5, 7, 12}, {5, 7}, {3, 5, 7, 15}, {5, 7, 9, 15}, {5, 7, 13, 15}, {5, 7, 14, 15}

{5, 9} → {2, 9}, {9}, {5}, {4, 5, 9}, {5, 7, 9}, {5, 9, 13}, {5, 9, 14}, {5, 9, 15}
{5, 9, 10} → {9, 10}, {5, 10}, {5, 9, 11}, {5, 9, 12}, {4, 5, 9, 10}, {5, 7, 9, 10}
{5, 9, 11} → {9, 11}, {5, 11}, {5, 9, 10}, {5, 9, 13}, {5, 9, 15}, {4, 5, 9, 11}, {5, 7, 9, 11}, {5, 9, 11, 14}
{5, 9, 11, 14} → {9, 11, 14}, {5, 11, 14}, {5, 9, 13, 14}, {5, 9, 14, 15}, {5, 9, 11}, {4, 5, 9, 11, 14}, {5, 7, 9, 11, 14}
{5, 9, 12} → {2, 9, 12}, {9, 12}, {5, 12}, {5, 9, 10}, {5, 9, 14}, {5, 9, 15}, {4, 5, 9, 12}, {5, 7, 9, 12}, {5, 9, 12, 13}
{5, 9, 12, 13} → {2, 9, 12, 13}, {9, 12, 13}, {5, 12, 13}, {5, 9, 13, 14}, {5, 9, 13, 15}, {5, 9, 12}, {4, 5, 9, 12, 13}, {5, 7, 9, 12, 13}
{5, 9, 13} → {2, 9, 13}, {9, 13}, {5, 13}, {5, 9, 11}, {5, 9}, {4, 5, 9, 13}, {5, 7, 9, 13}, {5, 9, 13, 14}, {5, 9, 13, 15}
{5, 9, 13, 14} → {2, 9, 13, 14}, {9, 13, 14}, {5, 13, 14}, {5, 9, 11, 14}, {5, 9, 14}, {5, 9, 12, 13}, {5, 9, 13}, {4, 5, 9, 13, 14}, {5, 7, 9, 13, 14}, {5, 9, 13, 14, 15}
{5, 9, 13, 14, 15} → {2, 9, 13, 14, 15}, {9, 13, 14, 15}, {5, 13, 14, 15}, {5, 9, 14, 15}, {5, 9, 13, 15}, {5, 9, 13, 14}, {4, 5, 9, 13, 14, 15}, {5, 7, 9, 13, 14, 15}
{5, 9, 13, 15} → {2, 9, 13, 15}, {9, 13, 15}, {5, 13, 15}, {5, 9, 15}, {5, 9, 12, 13}, {5, 9, 13}, {4, 5, 9, 13, 15}, {5, 7, 9, 13, 15}, {5, 9, 13, 14, 15}
{5, 9, 14} → {2, 9, 14}, {9, 14}, {5, 14}, {5, 9, 12}, {5, 9}, {4, 5, 9, 14}, {5, 7, 9, 14}, {5, 9, 13, 14}, {5, 9, 14, 15}
{5, 9, 14, 15} → {2, 9, 14, 15}, {9, 14, 15}, {5, 14, 15}, {5, 9, 15}, {5, 9, 11, 14}, {5, 9, 14}, {4, 5, 9, 14, 15}, {5, 7, 9, 14, 15}, {5, 9, 13, 14, 15}
{5, 9, 15} → {2, 9, 15}, {9, 15}, {5, 15}, {5, 9, 11}, {5, 9, 12}, {5, 9}, {4, 5, 9, 15}, {5, 7, 9, 15}, {5, 9, 13, 15}, {5, 9, 14, 15}
{5, 10} → {10}, {5, 11}, {5, 12}, {3, 5, 10}, {4, 5, 10}, {5, 7, 10}, {5, 9, 10}
{5, 11} → {1, 11}, {11}, {5, 10}, {5, 13}, {5, 15}, {3, 5, 11}, {4, 5, 11}, {5, 7, 11}, {5, 9, 11}, {5, 11, 14}
{5, 11, 14} → {1, 11, 14}, {11, 14}, {5, 13, 14}, {5, 14, 15}, {5, 11}, {3, 5, 11, 14}, {4, 5, 11, 14}, {5, 7, 11, 14}, {5, 9, 11, 14}
{5, 12} → {2, 12}, {12}, {5, 10}, {5, 14}, {5, 15}, {3, 5, 12}, {4, 5, 12}, {5, 7, 12}, {5, 9, 12}, {5, 12, 13}
{5, 12, 13} → {2, 12, 13}, {12, 13}, {5, 13, 14}, {5, 13, 15}, {5, 12}, {3, 5, 12, 13}, {4, 5, 12, 13}, {5, 7, 12, 13}, {5, 9, 12, 13}
{5, 13} → {1, 13}, {2, 13}, {13}, {5, 11}, {5}, {3, 5, 13}, {4, 5, 13}, {5, 7, 13}, {5, 9, 13}, {5, 13, 14}, {5, 13, 15}
{5, 13, 14} → {1, 13, 14}, {2, 13, 14}, {13, 14}, {5, 11, 14}, {5, 14}, {5, 12, 13}, {5, 13}, {3, 5, 13, 14}, {4, 5, 13, 14}, {5, 7, 13, 14}, {5, 9, 13, 14}, {5, 13, 14, 15}
{5, 13, 14, 15} → {1, 13, 14, 15}, {2, 13, 14, 15}, {13, 14, 15}, {5, 14, 15}, {5, 13, 15}, {5, 13, 14}, {3, 5, 13, 14, 15}, {4, 5, 13, 14, 15}, {5, 7, 13, 14, 15}, {5, 9, 13, 14, 15}
{5, 13, 15} → {1, 13, 15}, {2, 13, 15}, {13, 15}, {5, 15}, {5, 12, 13}, {5, 13}, {3, 5, 13, 15}, {4, 5, 13, 15}, {5, 7, 13, 15}, {5, 9, 13, 15}, {5, 13, 14, 15}
{5, 14} → {1, 14}, {2, 14}, {14}, {5, 12}, {5}, {3, 5, 14}, {4, 5, 14}, {5, 7, 14}, {5, 9, 14}, {5, 13, 14}, {5, 14, 15}
{5, 14, 15} → {1, 14, 15}, {2, 14, 15}, {14, 15}, {5, 15}, {5, 11, 14}, {5, 14}, {3, 5, 14, 15}, {4, 5, 14, 15}, {5, 7, 14, 15}, {5, 9, 14, 15}, {5, 13, 14, 15}
{5, 15} → {1, 15}, {2, 15}, {15}, {5, 11}, {5, 12}, {5}, {3, 5, 15}, {4, 5, 15}, {5, 7, 15}, {5, 9, 15}, {5, 13, 15}, {5, 14, 15}
{6, 9} → {7, 9}, {8, 9}, {6}, {6, 9, 13}, {6, 9, 14}, {6, 9, 15}, {0}
{6, 9, 12} → {7, 9, 12}, {8, 9, 12}, {6, 12}, {6, 9, 14}, {6, 9, 15}, {6, 9, 12, 13}
{6, 9, 12, 13} → {7, 9, 12, 13}, {8, 9, 12, 13}, {6, 12, 13}, {6, 9, 13, 14}, {6, 9, 13, 15}, {6, 9, 12}
{6, 9, 13} → {7, 9, 13}, {8, 9, 13}, {6, 13}, {6, 9}, {6, 9, 13, 14}, {6, 9, 13, 15}, {0, 13}
{6, 9, 13, 14} → {7, 9, 13, 14}, {8, 9, 13, 14}, {6, 13, 14}, {6, 9, 14}, {6, 9, 12, 13}, {6, 9, 13}, {6, 9, 13, 14, 15}, {0, 13, 14}
{6, 9, 13, 14, 15} → {7, 9, 13, 14, 15}, {8, 9, 13, 14, 15}, {6, 13, 14, 15}, {6, 9, 14, 15}, {6, 9, 13, 15}, {6, 9, 13, 14}, {0, 13, 14, 15}
{6, 9, 13, 15} → {7, 9, 13, 15}, {8, 9, 13, 15}, {6, 13, 15}, {6, 9, 15}, {6, 9, 12, 13}, {6, 9, 13}, {6, 9, 13, 14, 15}, {0, 13, 15}
{6, 9, 14} → {7, 9, 14}, {8, 9, 14}, {6, 14}, {6, 9, 12}, {6, 9}, {6, 9, 13, 14}, {6, 9, 14, 15}, {0, 14}
{6, 9, 14, 15} → {7, 9, 14, 15}, {8, 9, 14, 15}, {6, 14, 15}, {6, 9, 15}, {6, 9, 14}, {6, 9, 13, 14, 15}, {0, 14, 15}
{6, 9, 15} → {7, 9, 15}, {8, 9, 15}, {6, 15}, {6, 9, 12}, {6, 9}, {6, 9, 13, 15}, {6, 9, 14, 15}, {0, 15}
{6, 12} → {7, 12}, {8, 12}, {6, 14}, {6, 15}, {3, 6, 12}, {6, 9, 12}, {6, 12, 13}
{6, 12, 13} → {7, 12, 13}, {8, 12, 13}, {6, 13, 14}, {6, 13, 15}, {6, 12}, {3, 6, 12, 13}, {6, 9, 12, 13}
{6, 13} → {7, 13}, {8, 13}, {6}, {3, 6, 13}, {6, 9, 13}, {6, 13, 14}, {6, 13, 15}
{6, 13, 14} → {7, 13, 14}, {8, 13, 14}, {6, 14}, {6, 12, 13}, {6, 13}, {3, 6, 13, 14}, {6, 9, 13, 14}, {6, 13, 14, 15}
{6, 13, 14, 15} → {7, 13, 14, 15}, {8, 13, 14, 15}, {6, 14, 15}, {6, 13, 15}, {6, 13, 14}, {3, 6, 13, 14, 15}, {6, 9, 13, 14, 15}
{6, 13, 15} → {7, 13, 15}, {8, 13, 15}, {6, 15}, {6, 12, 13}, {6, 13}, {3, 6, 13, 15}, {6, 9, 13, 15}, {6, 13, 14, 15}
{6, 14} → {7, 14}, {8, 14}, {6, 12}, {6}, {3, 6, 14}, {6, 9, 14}, {6, 13, 14}, {6, 14, 15}
{6, 14, 15} → {7, 14, 15}, {8, 14, 15}, {6, 15}, {6, 14}, {3, 6, 14, 15}, {6, 9, 14, 15}, {6, 13, 14, 15}
{6, 15} → {7, 15}, {8, 15}, {6, 12}, {6}, {3, 6, 15}, {6, 9, 15}, {6, 13, 15}, {6, 14, 15}
{7, 8} → {8}, {7}, {3, 7, 8}, {7, 8, 9}, {7, 8, 13}, {7, 8, 14}, {7, 8, 15}
{7, 8, 9} → {8, 9}, {7, 9}, {7, 8}, {7, 8, 9, 13}, {7, 8, 9, 14}, {7, 8, 9, 15}
{7, 8, 9, 10} → {8, 9, 10}, {7, 9, 10}, {7, 8, 10}, {7, 8, 9, 11}, {7, 8, 9, 12}
{7, 8, 9, 11} → {8, 9, 11}, {7, 9, 11}, {7, 8, 11}, {7, 8, 9, 10}, {7, 8, 9, 13}, {7, 8, 9, 15}, {7, 8, 9, 11, 14}
{7, 8, 9, 11, 14} → {8, 9, 11, 14}, {7, 9, 11, 14}, {7, 8, 11, 14}, {7, 8, 9, 13, 14}, {7, 8, 9, 14, 15}, {7, 8, 9, 11}
{7, 8, 9, 12} → {8, 9, 12}, {7, 9, 12}, {7, 8, 12}, {7, 8, 9, 10}, {7, 8, 9, 14}, {7, 8, 9, 15}, {7, 8, 9, 12, 13}
{7, 8, 9, 12, 13} → {8, 9, 12, 13}, {7, 9, 12, 13}, {7, 8, 12, 13}, {7, 8, 9, 13, 14}, {7, 8, 9, 13, 15}, {7, 8, 9, 12}
{7, 8, 9, 13} → {8, 9, 13}, {7, 9, 13}, {7, 8, 13}, {7, 8, 9, 11}, {7, 8, 9}, {7, 8, 9, 13, 14}, {7, 8, 9, 13, 15}
{7, 8, 9, 13, 14} → {8, 9, 13, 14}, {7, 9, 13, 14}, {7, 8, 13, 14}, {7, 8, 9, 11, 14}, {7, 8, 9, 14}, {7, 8, 9, 12, 13}, {7, 8, 9, 13}, {7, 8, 9, 13, 14, 15}
{7, 8, 9, 13, 14, 15} → {8, 9, 13, 14, 15}, {7, 9, 13, 14, 15}, {7, 8, 13, 14, 15}, {7, 8, 9, 14, 15}, {7, 8, 9, 13, 15}, {7, 8, 9, 13, 14}
{7, 8, 9, 13, 15} → {8, 9, 13, 15}, {7, 9, 13, 15}, {7, 8, 13, 15}, {7, 8, 9, 15}, {7, 8, 9, 12, 13}, {7, 8, 9, 13}, {7, 8, 9, 13, 14, 15}
{7, 8, 9, 14} → {8, 9, 14}, {7, 9, 14}, {7, 8, 14}, {7, 8, 9, 12}, {7, 8, 9}, {7, 8, 9, 13, 14}, {7, 8, 9, 14, 15}
{7, 8, 9, 14, 15} → {8, 9, 14, 15}, {7, 9, 14, 15}, {7, 8, 14, 15}, {7, 8, 9, 15}, {7, 8, 9, 11, 14}, {7, 8, 9, 14}, {7, 8, 9, 13, 14, 15}
{7, 8, 9, 15} → {8, 9, 15}, {7, 9, 15}, {7, 8, 15}, {7, 8, 9, 11}, {7, 8, 9, 12}, {7, 8, 9}, {7, 8, 9, 13, 15}, {7, 8, 9, 14, 15}
{7, 8, 10} → {8, 10}, {7, 10}, {7, 8, 11}, {7, 8, 12}, {3, 7, 8, 10}, {7, 8, 9, 10}
{7, 8, 11} → {8, 11}, {7, 11}, {7, 8, 10}, {7, 8, 13}, {7, 8, 15}, {3, 7, 8, 11}, {7, 8, 9, 11}, {7, 8, 11, 14}
{7, 8, 11, 14} → {8, 11, 14}, {7, 11, 14}, {7, 8, 13, 14}, {7, 8, 14, 15}, {7, 8, 11}, {3, 7, 8, 11, 14}, {7, 8, 9, 11, 14}
{7, 8, 12} → {8, 12}, {7, 12}, {7, 8, 10}, {7, 8, 14}, {7, 8, 15}, {3, 7, 8, 12}, {7, 8, 9, 12}, {7, 8, 12, 13}
{7, 8, 12, 13} → {8, 12, 13}, {7, 12, 13}, {7, 8, 13, 14}, {7, 8, 13, 15}, {7, 8, 12}, {3, 7, 8, 12, 13}, {7, 8, 9, 12, 13}
{7, 8, 13} → {8, 13}, {7, 13}, {7, 8, 11}, {7, 8}, {3, 7, 8, 13}, {7, 8, 9, 13}, {7, 8, 13, 14}, {7, 8, 13, 15}
{7, 8, 13, 14} → {8, 13, 14}, {7, 13, 14}, {7, 8, 11, 14}, {7, 8, 14}, {7, 8, 12, 13}, {7, 8, 13}, {3, 7, 8, 13, 14}, {7, 8, 9, 13, 14}, {7, 8, 13, 14, 15}
{7, 8, 13, 14, 15} → {8, 13, 14, 15}, {7, 13, 14, 15}, {7, 8, 14, 15}, {7, 8, 13, 15}, {7, 8, 13, 14}, {3, 7, 8, 13, 14, 15}, {7, 8, 9, 13, 14, 15}
{7, 8, 13, 15} → {8, 13, 15}, {7, 13, 15}, {7, 8, 15}, {7, 8, 12, 13}, {7, 8, 13}, {3, 7, 8, 13, 15}, {7, 8, 9, 13, 15}, {7, 8, 13, 14, 15}
{7, 8, 14} → {8, 14}, {7, 14}, {7, 8, 12}, {7, 8}, {3, 7, 8, 14}, {7, 8, 9, 14}, {7, 8, 13, 14}, {7, 8, 14, 15}
{7, 8, 14, 15} → {8, 14, 15}, {7, 14, 15}, {7, 8, 15}, {7, 8, 11, 14}, {7, 8, 14}, {3, 7, 8, 14, 15}, {7, 8, 9, 14, 15}, {7, 8, 13, 14, 15}
{7, 8, 15} → {8, 15}, {7, 15}, {7, 8, 11}, {7, 8, 12}, {7, 8}, {3, 7, 8, 15}, {7, 8, 9, 15}, {7, 8, 13, 15}, {7, 8, 14, 15}
{7, 9} → {6, 9}, {9}, {7}, {5, 7, 9}, {7, 8, 9}, {7, 9, 13}, {7, 9, 14}, {7, 9, 15}

{7, 9, 10} → {9, 10}, {7, 10}, {7, 9, 11}, {7, 9, 12}, {5, 7, 9, 10}, {7, 8, 9, 10}
{7, 9, 11} → {9, 11}, {7, 11}, {7, 9, 10}, {7, 9, 13}, {7, 9, 15}, {5, 7, 9, 11}, {7, 8, 9, 11}, {7, 9, 11, 14}
{7, 9, 11, 14} → {9, 11, 14}, {7, 11, 14}, {7, 9, 13, 14}, {7, 9, 14, 15}, {7, 9, 11}, {5, 7, 9, 11, 14}, {7, 8, 9, 11, 14}
{7, 9, 12} → {6, 9, 12}, {9, 12}, {7, 12}, {7, 9, 10}, {7, 9, 14}, {7, 9, 15}, {5, 7, 9, 12}, {7, 8, 9, 12}, {7, 9, 12, 13}
{7, 9, 12, 13} → {6, 9, 12, 13}, {9, 12, 13}, {7, 12, 13}, {7, 9, 13, 14}, {7, 9, 13, 15}, {7, 9, 12}, {5, 7, 9, 12, 13}, {7, 8, 9, 12, 13}
{7, 9, 13} → {6, 9, 13}, {9, 13}, {7, 13}, {7, 9, 11}, {7, 9}, {5, 7, 9, 13}, {7, 8, 9, 13}, {7, 9, 13, 14}, {7, 9, 13, 15}
{7, 9, 13, 14} → {6, 9, 13, 14}, {9, 13, 14}, {7, 13, 14}, {7, 9, 11, 14}, {7, 9, 14}, {7, 9, 12, 13}, {7, 9, 13}, {5, 7, 9, 13, 14}, {7, 8, 9, 13, 14}, {7, 9, 13, 14, 15}
{7, 9, 13, 14, 15} → {6, 9, 13, 14, 15}, {9, 13, 14, 15}, {7, 13, 14, 15}, {7, 9, 14, 15}, {7, 9, 13, 15}, {7, 9, 13, 14}, {5, 7, 9, 13, 14, 15}, {7, 8, 9, 13, 14, 15}
{7, 9, 13, 15} → {6, 9, 13, 15}, {9, 13, 15}, {7, 13, 15}, {7, 9, 15}, {7, 9, 12, 13}, {7, 9, 13}, {5, 7, 9, 13, 15}, {7, 8, 9, 13, 15}, {7, 9, 13, 14, 15}
{7, 9, 14} → {6, 9, 14}, {9, 14}, {7, 14}, {7, 9, 12}, {7, 9}, {5, 7, 9, 14}, {7, 8, 9, 14}, {7, 9, 13, 14}, {7, 9, 14, 15}
{7, 9, 14, 15} → {6, 9, 14, 15}, {9, 14, 15}, {7, 14, 15}, {7, 9, 15}, {7, 9, 11, 14}, {7, 9, 14}, {5, 7, 9, 14, 15}, {7, 8, 9, 14, 15}, {7, 9, 13, 14, 15}
{7, 9, 15} → {6, 9, 15}, {9, 15}, {7, 15}, {7, 9, 11}, {7, 9, 12}, {7, 9}, {5, 7, 9, 15}, {7, 8, 9, 15}, {7, 9, 13, 15}, {7, 9, 14, 15}
{7, 10} → {10}, {7, 11}, {7, 12}, {3, 7, 10}, {5, 7, 10}, {7, 8, 10}, {7, 9, 10}
{7, 11} → {11}, {7, 10}, {7, 13}, {7, 15}, {3, 7, 11}, {5, 7, 11}, {7, 8, 11}, {7, 9, 11}, {7, 11, 14}
{7, 11, 14} → {11, 14}, {7, 13, 14}, {7, 14, 15}, {7, 11}, {3, 7, 11, 14}, {5, 7, 11, 14}, {7, 8, 11, 14}, {7, 9, 11, 14}
{7, 12} → {6, 12}, {12}, {7, 10}, {7, 14}, {7, 15}, {3, 7, 12}, {5, 7, 12}, {7, 8, 12}, {7, 9, 12}, {7, 12, 13}
{7, 12, 13} → {6, 12, 13}, {12, 13}, {7, 13, 14}, {7, 13, 15}, {7, 12}, {3, 7, 12, 13}, {5, 7, 12, 13}, {7, 8, 12, 13}, {7, 9, 12, 13}
{7, 13} → {6, 13}, {13}, {7, 11}, {7}, {3, 7, 13}, {5, 7, 13}, {7, 8, 13}, {7, 9, 13}, {7, 13, 14}, {7, 13, 15}
{7, 13, 14} → {6, 13, 14}, {13, 14}, {7, 11, 14}, {7, 14}, {7, 12, 13}, {7, 13}, {3, 7, 13, 14}, {5, 7, 13, 14}, {7, 8, 13, 14}, {7, 9, 13, 14}, {7, 13, 14, 15}
{7, 13, 14, 15} → {6, 13, 14, 15}, {13, 14, 15}, {7, 14, 15}, {7, 13, 15}, {7, 13, 14}, {3, 7, 13, 14, 15}, {5, 7, 13, 14, 15}, {7, 8, 13, 14, 15}, {7, 9, 13, 14, 15}
{7, 13, 15} → {6, 13, 15}, {13, 15}, {7, 15}, {7, 12, 13}, {7, 13}, {3, 7, 13, 15}, {5, 7, 13, 15}, {7, 8, 13, 15}, {7, 9, 13, 15}, {7, 13, 14, 15}
{7, 14} → {6, 14}, {14}, {7, 12}, {7}, {3, 7, 14}, {5, 7, 14}, {7, 8, 14}, {7, 9, 14}, {7, 13, 14}, {7, 14, 15}
{7, 14, 15} → {6, 14, 15}, {14, 15}, {7, 15}, {7, 11, 14}, {7, 14}, {3, 7, 14, 15}, {5, 7, 14, 15}, {7, 8, 14, 15}, {7, 9, 14, 15}, {7, 13, 14, 15}
{7, 15} → {6, 15}, {15}, {7, 11}, {7, 12}, {7}, {3, 7, 15}, {5, 7, 15}, {7, 8, 15}, {7, 9, 15}, {7, 13, 15}, {7, 14, 15}
{8, 9} → {6, 9}, {9}, {8}, {4, 8, 9}, {7, 8, 9}, {8, 9, 13}, {8, 9, 14}, {8, 9, 15}
{8, 9, 10} → {9, 10}, {8, 10}, {8, 9, 11}, {8, 9, 12}, {4, 8, 9, 10}, {7, 8, 9, 10}
{8, 9, 11} → {9, 11}, {8, 11}, {8, 9, 10}, {8, 9, 13}, {8, 9, 15}, {4, 8, 9, 11}, {7, 8, 9, 11}, {8, 9, 11, 14}
{8, 9, 11, 14} → {9, 11, 14}, {8, 11, 14}, {8, 9, 13, 14}, {8, 9, 14, 15}, {8, 9, 11}, {4, 8, 9, 11, 14}, {7, 8, 9, 11, 14}
{8, 9, 12} → {6, 9, 12}, {9, 12}, {8, 12}, {8, 9, 10}, {8, 9, 14}, {8, 9, 15}, {4, 8, 9, 12}, {7, 8, 9, 12}, {8, 9, 12, 13}
{8, 9, 12, 13} → {6, 9, 12, 13}, {9, 12, 13}, {8, 12, 13}, {8, 9, 13, 14}, {8, 9, 13, 15}, {8, 9, 12}, {4, 8, 9, 12, 13}, {7, 8, 9, 12, 13}
{8, 9, 13} → {6, 9, 13}, {9, 13}, {8, 13}, {8, 9, 11}, {8, 9}, {4, 8, 9, 13}, {7, 8, 9, 13}, {8, 9, 13, 14}, {8, 9, 13, 15}
{8, 9, 13, 14} → {6, 9, 13, 14}, {9, 13, 14}, {8, 13, 14}, {8, 9, 11, 14}, {8, 9, 14}, {8, 9, 12, 13}, {8, 9, 13}, {4, 8, 9, 13, 14}, {7, 8, 9, 13, 14}, {8, 9, 13, 14, 15}
{8, 9, 13, 14, 15} → {6, 9, 13, 14, 15}, {9, 13, 14, 15}, {8, 13, 14, 15}, {8, 9, 14, 15}, {8, 9, 13, 15}, {8, 9, 13, 14}, {4, 8, 9, 13, 14, 15}, {7, 8, 9, 13, 14, 15}
{8, 9, 13, 15} → {6, 9, 13, 15}, {9, 13, 15}, {8, 13, 15}, {8, 9, 15}, {8, 9, 12, 13}, {8, 9, 13}, {4, 8, 9, 13, 15}, {7, 8, 9, 13, 15}, {8, 9, 13, 14, 15}
{8, 9, 14} → {6, 9, 14}, {9, 14}, {8, 14}, {8, 9, 12}, {8, 9}, {4, 8, 9, 14}, {7, 8, 9, 14}, {8, 9, 13, 14}, {8, 9, 14, 15}
{8, 9, 14, 15} → {6, 9, 14, 15}, {9, 14, 15}, {8, 14, 15}, {8, 9, 15}, {8, 9, 11, 14}, {8, 9, 14}, {4, 8, 9, 14, 15}, {7, 8, 9, 14, 15}, {8, 9, 13, 14, 15}
{8, 9, 15} → {6, 9, 15}, {9, 15}, {8, 15}, {8, 9, 11}, {8, 9, 12}, {8, 9}, {4, 8, 9, 15}, {7, 8, 9, 15}, {8, 9, 13, 15}, {8, 9, 14, 15}
{8, 10} → {10}, {8, 11}, {8, 12}, {3, 8, 10}, {4, 8, 10}, {7, 8, 10}, {8, 9, 10}
{8, 11} → {11}, {8, 10}, {8, 13}, {8, 15}, {3, 8, 11}, {4, 8, 11}, {7, 8, 11}, {8, 9, 11}, {8, 11, 14}
{8, 11, 14} → {11, 14}, {8, 13, 14}, {8, 14, 15}, {8, 11}, {3, 8, 11, 14}, {4, 8, 11, 14}, {7, 8, 11, 14}, {8, 9, 11, 14}
{8, 12} → {6, 12}, {12}, {8, 10}, {8, 14}, {8, 15}, {3, 8, 12}, {4, 8, 12}, {7, 8, 12}, {8, 9, 12}, {8, 12, 13}
{8, 12, 13} → {6, 12, 13}, {12, 13}, {8, 13, 14}, {8, 13, 15}, {8, 12}, {3, 8, 12, 13}, {4, 8, 12, 13}, {7, 8, 12, 13}, {8, 9, 12, 13}
{8, 13} → {6, 13}, {13}, {8, 11}, {8}, {3, 8, 13}, {4, 8, 13}, {7, 8, 13}, {8, 9, 13}, {8, 13, 14}, {8, 13, 15}
{8, 13, 14} → {6, 13, 14}, {13, 14}, {8, 11, 14}, {8, 14}, {8, 12, 13}, {8, 13}, {3, 8, 13, 14}, {4, 8, 13, 14}, {7, 8, 13, 14}, {8, 9, 13, 14}, {8, 13, 14, 15}
{8, 13, 14, 15} → {6, 13, 14, 15}, {13, 14, 15}, {8, 14, 15}, {8, 13, 15}, {8, 13, 14}, {3, 8, 13, 14, 15}, {4, 8, 13, 14, 15}, {7, 8, 13, 14, 15}, {8, 9, 13, 14, 15}
{8, 13, 15} → {6, 13, 15}, {13, 15}, {8, 15}, {8, 12, 13}, {8, 13}, {3, 8, 13, 15}, {4, 8, 13, 15}, {7, 8, 13, 15}, {8, 9, 13, 15}, {8, 13, 14, 15}
{8, 14} → {6, 14}, {14}, {8, 12}, {8}, {3, 8, 14}, {4, 8, 14}, {7, 8, 14}, {8, 9, 14}, {8, 13, 14}, {8, 14, 15}
{8, 14, 15} → {6, 14, 15}, {14, 15}, {8, 15}, {8, 11, 14}, {8, 14}, {3, 8, 14, 15}, {4, 8, 14, 15}, {7, 8, 14, 15}, {8, 9, 14, 15}, {8, 13, 14, 15}
{8, 15} → {6, 15}, {15}, {8, 11}, {8, 12}, {8}, {3, 8, 15}, {4, 8, 15}, {7, 8, 15}, {8, 9, 15}, {8, 13, 15}, {8, 14, 15}
{9, 10} → {10}, {9, 11}, {9, 12}, {4, 9, 10}, {5, 9, 10}, {7, 9, 10}, {8, 9, 10}
{9, 11} → {11}, {9, 10}, {9, 13}, {9, 15}, {4, 9, 11}, {5, 9, 11}, {7, 9, 11}, {8, 9, 11}, {9, 11, 14}
{9, 11, 14} → {11, 14}, {9, 13, 14}, {9, 14, 15}, {9, 11}, {4, 9, 11, 14}, {5, 9, 11, 14}, {7, 9, 11, 14}, {8, 9, 11, 14}
{9, 12} → {12}, {9, 10}, {9, 14}, {9, 15}, {4, 9, 12}, {5, 9, 12}, {7, 9, 12}, {8, 9, 12}, {9, 12, 13}
{9, 12, 13} → {12, 13}, {9, 13, 14}, {9, 13, 15}, {9, 12}, {4, 9, 12, 13}, {5, 9, 12, 13}, {7, 9, 12, 13}, {8, 9, 12, 13}
{9, 13} → {13}, {9, 11}, {9}, {4, 9, 13}, {5, 9, 13}, {7, 9, 13}, {8, 9, 13}, {9, 13, 14}, {9, 13, 15}
{9, 13, 14} → {13, 14}, {9, 11, 14}, {9, 14}, {9, 12, 13}, {9, 13}, {4, 9, 13, 14}, {5, 9, 13, 14}, {7, 9, 13, 14}, {8, 9, 13, 14}, {9, 13, 14, 15}
{9, 13, 14, 15} → {13, 14, 15}, {9, 14, 15}, {9, 13, 15}, {9, 13, 14}, {4, 9, 13, 14, 15}, {5, 9, 13, 14, 15}, {7, 9, 13, 14, 15}, {8, 9, 13, 14, 15}
{9, 13, 15} → {13, 15}, {9, 15}, {9, 12, 13}, {9, 13}, {4, 9, 13, 15}, {5, 9, 13, 15}, {7, 9, 13, 15}, {8, 9, 13, 15}, {9, 13, 14, 15}
{9, 14} → {14}, {9, 12}, {9}, {4, 9, 14}, {5, 9, 14}, {7, 9, 14}, {8, 9, 14}, {9, 13, 14}, {9, 14, 15}
{9, 14, 15} → {14, 15}, {9, 15}, {9, 11, 14}, {9, 14}, {4, 9, 14, 15}, {5, 9, 14, 15}, {7, 9, 14, 15}, {8, 9, 14, 15}, {9, 13, 14, 15}
{9, 15} → {15}, {9, 11}, {9, 12}, {9}, {4, 9, 15}, {5, 9, 15}, {7, 9, 15}, {8, 9, 15}, {9, 13, 15}, {9, 14, 15}
{11, 14} → {13, 14}, {14, 15}, {11}, {3, 11, 14}, {4, 11, 14}, {5, 11, 14}, {7, 11, 14}, {8, 11, 14}, {9, 11, 14}
{12, 13} → {13, 14}, {13, 15}, {12}, {3, 12, 13}, {4, 12, 13}, {5, 12, 13}, {7, 12, 13}, {8, 12, 13}, {9, 12, 13}

{13, 14} → {11, 14}, {14}, {12, 13}, {13}, {3, 13, 14}, {4, 13, 14}, {5, 13, 14}, {7, 13, 14}, {8, 13, 14}, {9, 13, 14}, {13, 14, 15}
{13, 14, 15} → {14, 15}, {13, 15}, {13, 14}, {3, 13, 14, 15}, {4, 13, 14, 15}, {5, 13, 14, 15}, {7, 13, 14, 15}, {8, 13, 14, 15}, {9, 13, 14, 15}
{13, 15} → {15}, {12, 13}, {13}, {3, 13, 15}, {4, 13, 15}, {5, 13, 15}, {7, 13, 15}, {8, 13, 15}, {9, 13, 15}, {13, 14, 15}
{14, 15} → {15}, {11, 14}, {14}, {3, 14, 15}, {4, 14, 15}, {5, 14, 15}, {7, 14, 15}, {8, 14, 15}, {9, 14, 15}, {13, 14, 15}

To be completed...

Supplement references

[1] R. W. Lymn and E. W. Taylor. “Mechanism of adenosine triphosphate hydrolysis by actomyosin”. *Biochemistry* 10.25 (1971).

[2] E. Eisenberg and L. E. Greene. “The relation of muscle biochemistry to muscle physiology”. *Annu. Rev. Physiol.* 42.1 (1980).

[3] S. Marzen, H. G. Garcia, and R. Phillips. “Statistical Mechanics of Monod–Wyman–Changeux (MWC) Models”. *J. Mol. Biol.* 425.9 (2013).
